# Comprehensive Profiling of the Native and Modified Peptidomes of Raw Bovine Milk and Processed Milk Products

**DOI:** 10.3390/foods9121841

**Published:** 2020-12-10

**Authors:** Michele Wölk, Sanja Milkovska-Stamenova, Ralf Hoffmann

**Affiliations:** 1 Institute of Bioanalytical Chemistry, Faculty of Chemistry and Mineralogy, Universität Leipzig, 04103 Leipzig, Germany; michele.woelk@uni-leipzig.de (M.W.); Ralf.hoffmann@bbz.uni-leipzig.de (R.H.); 2 Center for Biotechnology and Biomedicine, Universität Leipzig, 04103 Leipzig, Germany

**Keywords:** infant formula, lactosylation, milk processing, peptidomics, raw milk, UHT milk

## Abstract

Bovine milk contains a variety of endogenous peptides, partially formed by milk proteases that may exert diverse bioactive functions. Milk storage allows further protease activities altering the milk peptidome, while processing, e.g., heat treatment can trigger diverse chemical reactions, such as Maillard reactions and oxidations, leading to different posttranslational modifications (PTMs). The influence of processing on the native and modified peptidome was studied by analyzing peptides extracted from raw milk (RM), ultra-high temperature (UHT) milk, and powdered infant formula (IF) by nano reversed-phase liquid chromatography coupled online to electrospray ionization (ESI) tandem mass spectrometry. Only unmodified peptides proposed by two independent software tools were considered as identified. Thus, 801 identified peptides mainly originated from α_S_- and β-caseins, but also from milk fat globular membrane proteins, such as glycosylation-dependent cell adhesion molecule 1. RM and UHT milk showed comparable unmodified peptide profiles, whereas IF differed mainly due to a higher number of β-casein peptides. When 26 non-enzymatic posttranslational modifications (PTMs) were targeted in the milk peptidomes, 175 modified peptides were identified, i.e., mostly lactosylated and a few hexosylated or oxidized peptides. Most modified peptides originated from α_S_-caseins. The numbers of lactosylated peptides increased with harsher processing.

## 1. Introduction

The bovine raw milk contains a variety of endogenous peptides. Many of them exert bioactive functions, such as immunomodulatory effects and antimicrobial or mineral binding activities [1,2]. Native peptides are cleaved from the proteins by proteases naturally present in milk [1]. Plasmin, the dominant protease in bovine milk, shows a high specificity for β-, α_S1_-, and α_S2_-casein with only low or no activity towards κ-casein, β-lactoglobulin, and α-lactalbumin [3,4,5]. Cathepsin D digests mostly β-casein and α-lactalbumin at two specific sites, whereas native β-lactoglobulin is resistant to cleavage [4]. Other important proteases in bovine milk are elastase and cathepsin B [6].

Studies on the peptidome of raw milk identified high numbers of α- and β-casein derived peptides, mostly explained by activities of plasmin, cathepsin B and D, and elastase [7,8]. Peptidome analyses of milk indicated higher activities of cathepsin D and elastase in cows suffering from mastitis than in healthy cows and thus increased numbers and abundances of endogenous peptides in milk from infected cows [9,10]. Moreover, the peptide profile of colostrum sweet whey permeate, a by-product from cheese production, contained mainly β-casein derived peptides [11]. All these studies showed that the bovine milk peptidome is dominated by α- and β-casein derived peptides, whereas peptides from whey proteins and κ-casein are present at low contents or were not even detected [7,8,9,10,11].

The peptidome changes also during processing and storage. It is well known that the activity of plasmin is higher in pasteurized than in raw milk [12], as the enzyme activity enhances up to 75 °C, but decreases at higher temperatures [13]. Importantly, the plasmin activity is restored during storage due to activation of plasminogen remaining in heated milk [13]. Similarly, cathepsin D partially survives high-temperature short time (HTST) pasteurization [14]. Furthermore, the quantities of several peptides, mainly derived from β-casein, increased upon storage and differed significantly between stored and fresh ultra-high temperature (UHT) treated milk [15]. Consequently, the endogenous peptidome of milk is influenced by many factors.

Besides differences caused by protease activities during heating and storage, the milk peptidome may be altered further by chemical reactions induced or enhanced during processing. Due to the high contents of lactose and lysine residues, milk is prone towards Maillard reactions where a reducing sugar reacts with a free amino group to form so-called Amadori products, e.g., lactulosyllysine. A major consequence of these reactions is blocked lysine residues and a reduced nutritional value [16]. Such non-enzymatic modifications formed by Maillard reactions during thermal treatments are well studied at the protein level [16,17,18,19,20,21,22]. For instance, the lactosylation degrees of lysine residues increase with the harshness of the processing conditions [21,23]. These modifications may influence proteases as indicated by a model study which showed that lactosylation affected plasmin digestion of α- and κ-casein and to a lower extent of β-casein, whereas the digestion of lactosylated proteins by cathepsin D and chymosin was not affected [24]. Amadori products can undergo further reactions yielding diverse non-enzymatic posttranslational modifications (PTMs) known as advanced glycation end-products (AGEs), which can also be formed by dicarbonyls modifying mostly lysine and arginine residues [16,25]. Furthermore, oxidation including carbonylation sites can be formed by different pathways, such as metal-catalyzed oxidation (MCO), lipid peroxidation products (LPPs), or reactive dicarbonyls formed as intermediates of Maillard reactions or as lipid oxidation products [26,27]. All mentioned PTM types have been reported in the milk proteome [23,28,29,30,31,32]. Similar studies targeting modifications in the native milk peptidome including non-enzymatic PTMs are lacking.

Therefore, this study aimed at a thorough characterization of processing related changes in the peptidomes of raw milk (RM) and processed milk products including UHT milk (produced from the same RM batch), and powdered infant formula (IF). NanoRPC-ESI-MS/MS of the peptide extracts and a sophisticated processing strategy using different software tools identified 801 unmodified peptides originating mainly from α_S1_- and β-casein. The majority of peptides were present in all samples, with a high similarity for RM and UHT milk, whereas IF contained more β-casein derived peptides. Furthermore, 26 PTMs resulting from glycation and oxidation of different residues were targeted in the free peptidome. In total, 175 modified peptides originating from seven milk proteins were identified. The number of modified peptides, mostly lactosylated peptides, increased with the harshness of processing.

## 2. Materials and Methods 

### 2.1. Chemicals

Solvents that meet the highest demand for ultra-high-performance liquid chromatography (UHPLC), i.e., ULC-MS grade including methanol (ULC-MS grade, >99.97%), acetonitrile (ULC-MS grade, >99.97%), and formic acid (ULC-MS grade, >99%) were purchased from Biosolve B.V. (Valkenswaald, The Netherlands). Chloroform (≥99.8%) was obtained from Merck KgaA (Darmstadt, Germany). Water (resistance R > 18 mΩ/cm; total organic content <10 ppb) was purified by a PureLab Ultra Analytic system (ELGA Lab Water, Celle, Germany).

### 2.2. Peptide Extraction

RM and the corresponding UHT milk (3.5% fat), which was first pasteurized at ≥72.5 °C for at least 15 s and then UHT treated (140 °C for 3 s), were obtained from a local dairy company and stored at −80 °C. IF (nutritional values: 36 g/L fat, 71 g/L lactose, and 14 g/L proteins originating from skimmed milk and sweet whey) was bought at a local supermarket and prepared according to the manufacturer’s instructions. Peptides were extracted from three aliquots of each sample (50 µL) using a Folch extraction protocol [19]. Briefly, methanol and chloroform were added and the samples incubated (1 h, 4 °C). After the addition of water and a second incubation (10 min, 4 °C), the samples were centrifuged (10 min, 4 °C; 10,000× *g*), the organic phase removed and centrifuged again using the same conditions. The aqueous phase was dried under vacuum, reconstituted in aqueous acetonitrile (3%, *v*/*v*) containing formic acid (0.1%, *v*/*v*), and desalted by solid-phase extraction (SPE, Oasis HLB 1cc, 30 mg, Waters GmbH, Eschborn, Germany) [19]. The dried eluates were dissolved in aqueous acetonitrile (3%, *v*/*v*) containing formic acid (0.1%, *v*/*v*) and peptide concentrations determined on a NanoPhotometer NP80 (IMPLEN, Munich, Germany, λ= 280 nm).

### 2.3. Tandem Mass Spectrometry

Peptides were analyzed on a nanoAcquity UPLC (Waters GmbH, Eschborn, Germany) coupled on-line to an LTQ Orbitrap XL ETD mass spectrometer equipped with a nano-ESI source (Thermo Fisher Scientific, Bremen, Germany). After trapping (nanoAcquity Symmetry C18-column) at a flow rate of 5 µL/min (1% eluent B), peptides were separated on a BEH 130 column (30 °C) using a flow rate of 0.4 µL/min. Eluent A and B were water containing formic acid (0.1%, *v*/*v*) and acetonitrile containing formic acid (0.1%, *v*/*v*), respectively. Peptides were eluted by a two-step linear gradient increasing eluent B from 1% to 40% within 89 min and further to 85% within 5 min. The transfer capillary temperature was set to 200 °C and an ion spray voltage of 1.4 kV was applied to a PicoTip^TM^ on-line nano-ESI emitter (New Objective, Berlin, Germany). Mass spectra were recorded in the Orbitrap mass analyzer (*m*/*z* range 400 to 2000) at a resolution of 60,000 at *m*/*z* 400. Tandem mass spectra were acquired using data-dependent acquisition mode (DDA) for the six most intense signals in collision-induced dissociation (CID) mode as described before (isolation width of 2 *m*/*z* units, normalized collision energy of 35%, activation time of 30 s, default charge state of 2, intensity threshold of 500 counts, and dynamic exclusion window of 60 s) [22]. The samples were reanalyzed (after pooling the replicates for each sample) using a retention time based (±1.5 min) exclusion list of proposed unmodified peptides and the conditions described above. For modified peptides, tandem mass spectra were acquired for individual samples using electron transfer dissociation (ETD, isolation width of 2 *m*/*z* units, activation time 100 ms, default charge state 2, intensity threshold of 500 counts, and dynamic exclusion window of 60 s) in DDA mode for the six most intense signals [22]. Modified peptides proposed in previous measurements and preliminary experiments were targeted as well. 

### 2.4. Data Processing

#### 2.4.1. Unmodified Peptides

Acquired data were processed with Sequest using Proteome Discoverer 2.2 (Version 2.2.0.388, Thermo Fisher Scientific, Bremen, Germany) and PEAKS Studio 10.5 (Bioinformatics Solutions Inc., Waterloo, ON, Canada) using the following database and search parameters: bovine milk database (release 2016_11) [31], no enzyme, precursor mass tolerance 10 ppm, fragment mass tolerance 0.8 Da, false discovery rate 1%, and dynamic modifications including oxidation of methionine (+15.99 Da, Ox) and phosphorylation of serine (+79.96 Da, Phospho). As data processing relied on native peptides, i.e., searches were performed with no enzyme specificity, the processing times were very long and thus it was necessary to use a smaller, in-house milk-specific database. 

Peptides identified in at least two of the three replicates by both software tools were considered for further processing (Figure S1). Their presence in all measured samples was confirmed with Skyline (20.1.0.155, MacCoss Lab, Department of Genome Sciences, University of Washington) by generating a spectral library [33] and adjusting the parameters for the instrument used. Peptides were considered present if the precursor was detected at the same retention time and the isotope dot-product value (idotp) was above 0.95 in at least two individual replicates. 

#### 2.4.2. Modified Peptides

Acquired data were processed with Sequest within Proteome Discoverer 2.2 (as described above) additionally targeting 26 modifications (lactosylation, hexosylation, 12 AGEs, and 12 oxidation/carbonylation types) listed in Table S1. As Proteome Discoverer allows only six dynamic modifications per template at the same time, modifications were split into six templates. Proposed modified peptides were combined into an inclusion list that was used to analyze the individual milk samples again by DDA in ETD mode (Figure S1). Identification of modified peptides relied on peptides proposed by Proteome Discoverer 2.2 and manual confirmation of proposed modification sites. The presence of the confirmed modified peptides within all three sample types was confirmed within a spectral library generated in Skyline as described above for unmodified peptides. 

## 3. Results

### 3.1. Native Peptidome

**Figure 1:**
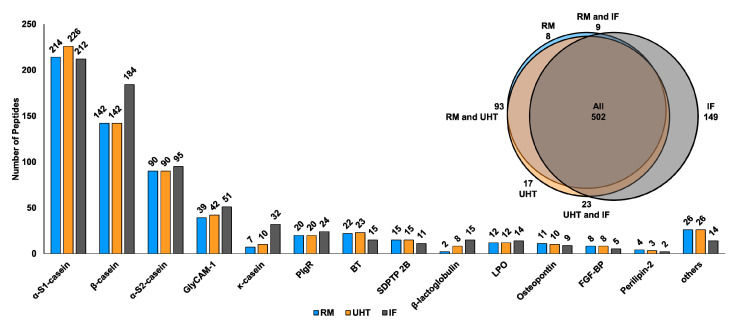
Number of unmodified peptides identified for different proteins in raw milk (RM, blue), ultra-high temperature (UHT, orange) treated milk, and infant formula (IF, grey). The insert shows a Venn diagram displaying the numbers of identified peptides among RM, UHT milk, and IF. Abbreviations: GlyCAM-1—glycosylation-dependent cell adhesion molecule 1, PigR—polymeric immunoglobulin receptor, BT—butyrophilin subfamily 1 member A1, SDPTP 2B—sodium-dependent phosphate transport protein 2B, LPO—lactoperoxidase, and FGF-BP—fibroblast growth factor-binding protein 1.

The data sets acquired for peptides in RM, UHT milk, and IF were processed by two different software packages relying on different strategies (Proteome Discoverer 2.2 and PEAKS Studio 10.5) to obtain a confident identification of unmodified peptides in at least two replicates of each milk sample. Their presence among all samples was confirmed after integrating the data into a spectral library within Skyline. This strategy identified 801 unmodified peptides originating from 36 different milk proteins (Table S2). The peptide length ranged from seven to 64 residues corresponding to peptides from 801.44 to 6783.34 Da with an average peptide length of 15.4 residues and an average peptide mass of 1766.86 Da. Although 502 peptides were present in all sample types, more peptides were detected in the processed milk products than in raw milk (Figure 1), i.e., 683 peptides from 25 proteins in IF, 635 peptides from 31 proteins in UHT milk, and 612 peptides from 30 proteins in RM. Interestingly, the peptidomes of RM and UHT milk overlapped by more than 95%, whereas 149 peptides present in IF were not detected in RM and UHT milk (Figure 1). 

About 70% of the identified peptides originated from α- and β-caseins (Figure 1 and Figure S2, and Table S2) including 246 peptides from α_S1_-casein. Around 77% of the α_S1_-casein-derived peptides were present in all sample types (Figure 1 and Figure S2). UHT milk contained slightly more peptides (226) compared to RM (214) and IF (214). Independent of the sample type, most peptides originated from three protein regions, i.e., Gly10 to Val37, His80 to Met123, and Ser180 to Trp199 (Figure S3). Interestingly, one peptide corresponding to Glu69 to Lys79 was present only in IF (Figure S3). Regions Gln59 to Ser68 and Gln155 to Tyr165 were not represented by any detected peptide (Figure S3). 

**Figure 2:**
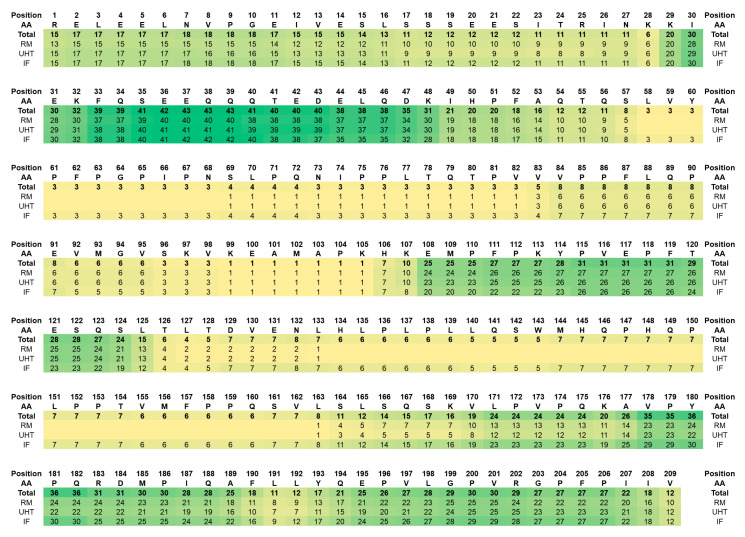
Protein sequence of β-casein without the signal peptide. The numbers below the sequence indicate how many peptides containing this specific residue were detected over all samples (total) and in RM (sequence coverage 80.9%), UHT milk (sequence coverage 80.9%), and IF (sequence coverage 100%). AA denotes amino acid.

With 202 β-casein-derived peptides, this protein was the second most dominant source of free peptides (Figure 1 and Figure S2; and Table S2) whereof 142 were present in RM and UHT milk and 184 in IF (Figure 1). The peptides from IF covered the full protein sequence except for the signal peptide (Figure 2), whereas sequences Leu58 to Asn68 and His134 to Val162 were missing in RM and UHT milk (Figure 2). However, most β-casein peptides originated from three parts of the sequence, i.e., Lys29 to Ala53, Glu108 to Ser124, and Leu171 to Ile207 (Figure 2). 

Around two-thirds of the 111 peptides derived from α_S2_-casein were present in all three milk types (Figure S2 and Table S2). Although these numbers are lower than for the other caseins, α_S2_-casein also showed four regions where most peptides originated from, i.e., Ser13 to Lys21, Gly102 to Lys113, Val139 to Lys149, and Leu153 to Phe163 (Figure S4). A relatively long sequence from Asn25 to Lys70 was not covered by an unmodified peptide (Figure S4). Peptides from Ala81 to Gln97 and Lys166 to Arg170 were found solely in IF (Figure S4). 

Peptides related to the glycosylation-dependent cell adhesion molecule 1 (GlyCAM-1) were the most common among the group of non-casein-protein-derived peptides. In total, 56 peptides corresponding mostly to regions Ile1 to Phe22 and Ser54 to Lys73 were identified, with 60.7% present in RM (39 peptides detected), UHT milk (42 peptides), and IF (51 peptides) (Figure 1, Figures S2 and S5, Table S2). Sequence Arg76 to Met108 was not represented by any peptide (Figure S5). The increase in IF peptides was mainly due to a higher number of peptides originating from the C-terminal part of GlyCAM-1 (Figure S5). 

Thirty-four peptides derived from κ-casein were detected in all (Figure 1 and Figure S2, and Table S2) with 32 present in IF, ten in UHT milk, and seven in RM. Only 12% of the identified peptides were common for all samples (Figure 1 and Figure S2). Most peptides originated from the C-terminal sequence Val152 to Val169, whereas regions Val31 to Val48 and Met106 to Glu147 were only present in IF (Figure S6). No peptides corresponding to regions Gln1 to Lys13, Tyr25 to Tyr30, and Ser80 to Phe105 of κ-casein were identified (Figure S6).

Besides peptides derived from caseins and GlyCAM-1, peptides originating from polymeric immunoglobulin receptor (PIgR, 27 peptides), butyrophilin subfamily 1 member A1 (BT, 23 peptides), β-lactoglobulin (16 peptides), lactoperoxidase (LPO, 15 peptides), sodium-dependent phosphate transport protein 2B (SDPTP 2B, 15 peptides), osteopontin (11 peptides), fibroblast growth factor-binding protein 1 (FGF-BP, 8 peptides), perilipin-2 (4 peptides), and 23 other milk proteins were identified (Figure 1 and Figure S2, and Table S2). Interestingly, β-lactoglobulin, as the major bovine whey protein, was only represented by two peptides in RM. However, eight peptides were detected in UHT milk and 15 peptides in IF (Figure 1), with only one peptide identified in all samples (Figure S2). 

### 3.2. Non-Enzymatic Modifications in the Bovine Milk Peptidome

**Figure 3:**
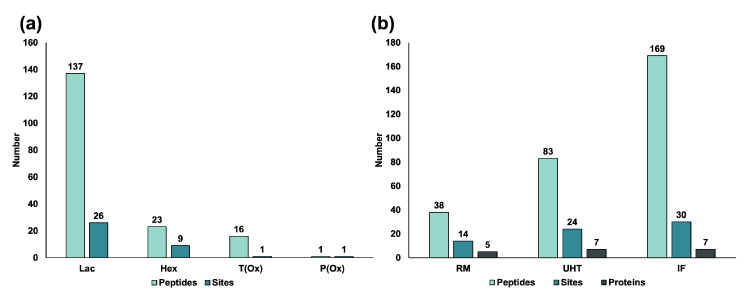
(**a**) Numbers of modified peptides identified with at least one lactosylation (Lac), hexosylation (Hex), and oxidation site in all milk samples (T(Ox) and P(Ox); (**b**) Number of modified peptides, modification sites, and corresponding proteins identified in RM, UHT milk, and IF.

**Figure 4:**
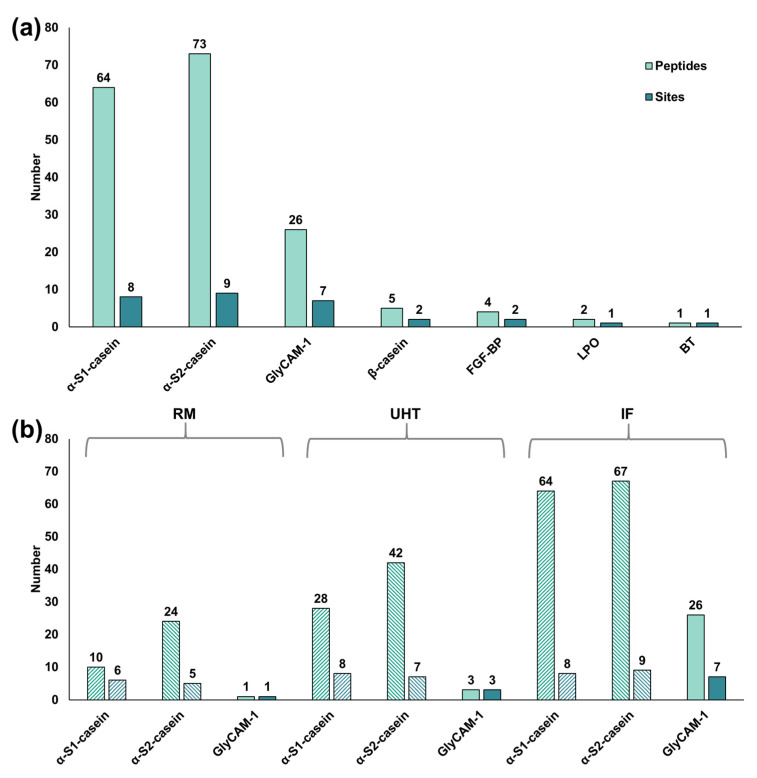
(**a**) Number of peptides and modification sites identified per modified protein in all milk samples; (**b**) Number of peptides (first bar) and modification sites (second bar) identified per protein in RM, UHT milk, and IF. Abbreviations: FGF-BP—fibroblast growth factor-binding protein 1, LPO—lactoperoxidase, and BT—butyrophilin subfamily 1 member A1.

Twenty-six glycation, AGE- and oxidation/carbonyl-related modifications (Table S1) were targeted in peptides present in RM, UHT milk, and IF. By data processing of the tandem mass spectra and manual confirmation of all proposed sequences, 175 peptides corresponding to seven milk proteins, i.e., α_S1_-, α_S2_-, and β-casein, GlyCAM-1, FGF-BP, LPO, and BT, containing 30 unique modification sites were confidently identified (Table S3). More than three-quarters of the peptides (137) carried a lactosylated lysine representing 26 unique modification sites (Figure 3a). Furthermore, 23 hexosylated peptides (nine modification sites), as well as one oxidized threonine and one oxidized proline residues (Figure 3a), were identified. The numbers of modified peptides increased from RM (38) to UHT milk (83) and further to IF (169), similar to the number of modification sites increasing from RM (14) to UHT milk (24) and to IF (30) (Figure 3b). Most modified peptides were derived from α_S1_- (64) and α_S2_-casein (73) corresponding to eight and nine modification sites, respectively (Figure 4a, Table S3). Interestingly, 26 modified peptides originated from GlyCAM-1 representing seven modification sites (Figure 4a, Table S3), whereas only a few modified peptides derived from β-casein (5) and FGF-BP (4) corresponding to two unique modification sites in each protein (Figure 4a, Table S3). LPO and BT had only one modification site per protein (Figure 4a, Table S3) in the processed milk samples identified. 

In particular, the total number of modified peptides originating from α_S1_-, α_S2_-casein, and GlyCAM-1 increased from RM to UHT milk and further to IF (Figure 4b). For example, the numbers of peptides detected for α_S1_-casein increased from ten (six modification sites) in RM to 28 (eight sites) in UHT milk, and 64 (eight sites) in IF (Figure 4b). All modification sites corresponded to lysine residues with half of them (Lys34, Lys36, Lys83, and Lys105) being located in regions where most unmodified peptides originated from (Figure S7). Additionally, modified peptides were derived from the regions Arg1 to Lys42 and His80 to Lys124 (Figure S7). Interestingly, RM was lacking peptides from Phe24 to Lys42. 

**Figure 5:**
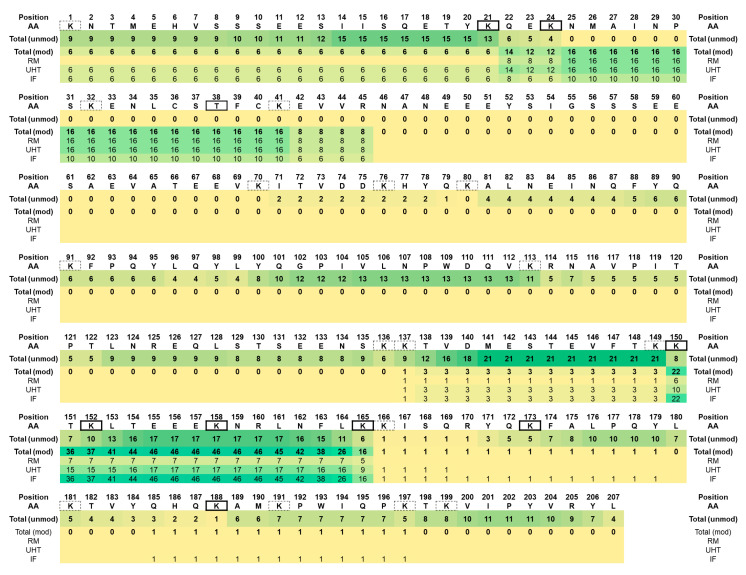
Sequence of α_S2_-casein without the signal peptide. The numbers below the sequence indicate how many peptides containing this specific residue were detected in all samples as unmodified (unmod) and/or modified (mod) in RM, UHT milk, and IF. Lysine residues of the protein are framed with a dotted line, and modification sites are fully framed.

Similarly, the numbers of modified α_S2_-casein-derived peptides increased from RM (24) to UHT milk (42), and IF (67) and the number of modification sites from five in RM, to seven in UHT milk, and nine in IF (Figure 4b, α_S2_-casein). In contrast to α_S1_-casein, only two modification sites were located in regions with a high density of unmodified peptides (Lys21 and Lys158), whereas most modified peptides corresponded to four modification sites from Lys150 to Lys165 (Figure 5). Besides eight glycated Lys residues (Lys21, 24, 150, 152, 165, 173, and 188), Thr38 was identified as oxidized and, interestingly, only modified peptides from this part of the protein sequence (Figure 5, Asn24 to Arg45) were identified. Lys173 and Lys188 were modified only in IF (Figure 5, Table S3) whereas Lys3, Lys7, Lys32, and Lys34 were not modified in RM (Figure 5). Although only 26 modified peptides originated from GlyCAM-1, they followed a similar trend (Figure 4b), i.e., the numbers increased from one peptide modified at one Lys residue identified in RM to three unique modification sites in UHT milk, and 26 peptides were identified in IF (Figure S8 and Table S3). Most of these peptides corresponded to regions dominantly represented by unmodified peptides, i.e., Ile1 to His10 and Ser54 to Lys73 (Figure S8). 

## 4. Discussion

Milk peptidomics typically relies on milk skimming by centrifugation, protein precipitation (e.g., trichloroacetic acid), and SPE [7,8], whereas milk proteomics often utilizes the Folch procedure prior to digestion and SPE [19,21,31]. In our hands, both protocols showed similar results for the extraction of endogenous peptides from a UHT milk (without skimming), but slightly more peptides were detected after the Folch procedure. Therefore, we applied this procedure to extract endogenous peptides from RM, UHT milk, and IF. Confident peptide identification was aimed for by utilizing two fragmentation techniques (i.e., CID and ETD) in DDA mode, processing the data with two software tools (i.e., Proteome Discoverer 2.2 and PEAKS Studio 10.5), and validation with a spectral library generated within Skyline as previously described by Dallas and Nielsen [33], which allowed confirming the presence of proposed peptides among all analyzed samples (Figure S1). To identify more modified peptides detected with low intensities, previously identified unmodified peptides were excluded from the second analysis of each milk sample. All modified peptides were confirmed by ETD, which is more suitable for the identification of glycated peptides due to the dominant cleavage of the backbone [19]. Finally, tandem mass spectra of proposed modification sites were confirmed by manual interpretations.

### 4.1. Native Peptidome

Most peptidomic studies focused on endogenous peptides in raw milk of healthy cows, cows with mastitis, or different species [7,8,9,10,34]. The 801 peptides identified here correspond well to the reported sequences, at the same time expanding the bovine milk peptidome (Table S2). However, some previously identified peptides were missed here, as modifications such as pyroglutamate formation of N-terminal glutamine [8,10] were not considered or peptides of different lengths were identified. Peptides identified by our approach ranged from seven to 64 residues, with an average length of 15.4. Shorter or longer peptides might also be present in bovine milk, but might have been missed due to their low abundances, poor ionization properties, ionization suppression effects, or the low efficacy of the applied LC-MS techniques to identify confidently peptides shorter than five or longer than 64 residues. However, the identification of 612 peptides derived from 30 proteins in RM (Figure 1, Table S2) is much higher than the 159 peptides reported in bovine milk of six individual healthy cows [8] and the 248 peptides in a raw milk pool [7]. In comparison to the endogenous peptides reported in raw milk from healthy and diseased (mastitis) cows, a slightly lower number of peptides was observed, possibly attributed to a higher release of peptides in diseased cows [10]. Independent of the sample type, peptides were mostly derived from α_S1_-casein, β-casein, α_S2_-casein, and GlyCAM-1 being in good agreement with the literature [7,8,10]. Similarly, peptides originating from κ-casein, PIgR, BT, β-lactoglobulin, LPO, osteopontin, and other minor milk proteins were previously identified in raw milk [8,10]. Interestingly, many of the identified proteins, such as GlyCAM-1, BT, mucin-1, mucin-15, and xanthine dehydrogenase/oxidase belong to the group of milk fat globule membrane (MFGM) proteins. 

As most studies focused on unprocessed milk, little is known about changes in the peptide profile along the processing chain of milk products. The current study analyzed samples from RM and the corresponding UHT milk collected after industrial processing (first pasteurized at min. 72.5 °C for at least 15 s and subsequently UHT treated at 140 °C for 3 s) to judge the changes between RM and UHT milk. For most proteins, the same peptides were identified (Figure 1). However, the numbers of α_S1_-casein-, κ-casein-, GlyCAM-1-, and β-lactoglobulin-derived peptides slightly increased in UHT milk. The higher number of κ-casein-derived peptides might be attributed to the higher levels of κ-casein present in the serum phase due to its depletion from the casein micelle at temperatures above 70 °C [35]. It should be noted that no peptides from α-lactalbumin and only 16 from β-lactoglobulin were identified in total, which may indicate low activity of plasmin and cathepsin D towards these proteins [3,4,36]. Alternatively, they might have been missed due to low quantities or the Folch protocol. A recent study reported that the contents of specific α_S1_- and β-casein-derived peptides increased during the storage of UHT milk [15]. Here, several of these marker peptides were also identified in RM and UHT milk (Table S2), however, quantitative analysis was beyond the scope of this study.

Most peptides were detected in IF (Figure 1), i.e., 683 peptides from 25 proteins. In particular, the number of β-casein, β-lactoglobulin, GlyCAM-1, and κ-casein peptides was higher compared to RM and UHT milk. The information about the protein sources provided on the original package indicates that this IF was produced from skimmed milk and sweet whey, which is the remaining liquid in cheese production after casein precipitation by rennet coagulation. A peptidomic study of whey permeate from colostrum found predominantly peptides from β-casein, α_S1_-, and κ-casein besides peptides corresponding to GlyCAM-1, PIgR, α_S2_-casein, and serum amyloid A [11]. As whey permeate is part of sweet whey, the increasing numbers of IF peptides might originate from the dried sweet whey powder added during IF production. Moreover, the peptidome determined from a whey protein isolate (WPI) revealed peptides originating mainly from β- and α_S1_-casein, followed by β-lactoglobulin [37]. Whey proteins are added to IF to increase the ratio of whey proteins to caseins from 20:80 in bovine milk to better resemble the human milk composition with a ratio of approximately 60:40 [38]. However, these are just assumptions from the presented data, as no further details about the added sweet whey were available. Alternatively, the increase of κ-casein-derived peptides can be explained by the cleavage of κ-casein between Phe105 and Met106 during rennet coagulation of caseins in the course of cheese making, leading to the diffusion of glycomacropeptide (GMP, also called caseinomacropeptide, CMP), i.e., the C-terminal fragment starting at Met106, into the whey phase [35,39]. It is worth mentioning that GMP has been identified with its full sequence in IF (Table S2, Peptide 697). Hence, mapping of κ-casein peptides underlined that peptides present only in IF originate mainly from the GMP region, especially between Met106 and Glu147, whereas peptides present in all samples are mainly derived from the subsequent C-terminal part (Figure S6, Ser149 to Val169). The low number of κ-casein-derived peptides, especially for RM and UHT milk, are in good agreement with previous studies [7,8,10], whereas the higher numbers detected in sweet whey permeate match the trend seen for IF [11]. Many peptides reported for whey permeate overlap with peptides identified here in IF. However, some parts of the protein were not represented by peptides, for example, the region from Ser80 to Phe105, which might be less prone to proteolysis. The majority of the peptides corresponded to the C-terminal part of the protein. Similarly, regions Leu35 to Tyr52 and Asn25 to Lys70 of α_S2_-casein were not covered by any peptide in the current study (Figure S4), although a few peptides corresponding to a part of the second region were reported for raw milk [10]. Alpha S1 casein (Figure S3) was mostly resembled by peptides identified here and a few missing parts of the sequence were identified in an earlier study [10]. 

Interestingly, the β-casein peptides identified in IF covered the complete protein sequence although this protein is longer than the other caseins (Figure 2). Peptides from a few sequence regions were absent in RM and UHT milk, but peptides covering these regions were previously identified in raw and UHT milk [10,11,15]. Similarly to our observations in IF, a recent study focusing on the analysis of in vitro digests of human milk and IF identified peptides from the caseins, β-lactoglobulin, and some minor milk proteins including the N- and C-termini of β casein where most peptides originated from [40]. 

This study focused on the processing of related changes in the milk peptidome without considering peptide bioactivities. However, several well-known bioactive peptides were identified. For example, β-casein peptides Ala177 to Arg183 and Tyr193 to Arg202, which belong to the class of β-casokinins with known ACE-inhibitory properties [1], are located in regions represented by many unmodified peptides. A similar trend was observed for a β-casein phosphopeptide (Lys29 to Thr41) with mineral binding properties [1]. Moreover, α_S1_ casokinin (Pep 40) with ACE-inhibitory properties, as well as sequences of antimicrobial peptides caseicins B (Pep 32), C (Pep 205), and A (Pep 79), were present in slightly longer sequences (Table S2) [1,41]. 

### 4.2. Non-Enzymatic Modifications

Many proteomic studies have targeted a variety of non-enzymatic PTMs, such as glycation, AGEs, and oxidations, in diverse milk samples including raw, pasteurized, UHT milk, and IF [18,19,20,23,29,31,42,43]. Generally, the modification degrees and the number of modified residues increased with harsher processing conditions depending on the modification type. Although endogenous peptides are far less abundant than proteins, they might also be a target of the same chemical reactions during milk processing. Additionally, modified peptides might be released from proteins by proteases. However, studies on the modified milk peptidome are still lacking despite their possible effect on bioactive peptides. Therefore, this study aimed to comprehensively characterize 26 diverse PTMs in endogenous milk peptides. However, only four different modifications, i.e., two glycation- (lactosylation and hexosylation) and two oxidation-products (T(Ox) and P(Ox)) were identified in 175 peptides originating from seven proteins that were also represented with unmodified peptides, i.e., α_S1_-casein, α_S2_-casein, β-casein, BT, FGF-BP, GlyCAM-1, and LPO. Despite the fact that unmodified peptides from α_S1_-casein were most common, the highest number of modified peptides originated from α_S2_-casein, followed by α_S1_-casein, and GlyCAM-1. Notably, only five modified compared to 202 unmodified β-casein peptides were identified, particularly in IF. The increasing numbers of modified peptides from RM to UHT milk and further to IF resulted from lactosylation, which is in agreement with bottom-up proteomic studies of diverse milk samples showing increasing numbers and quantities of lactosylated peptides along with harsher processing conditions [19,22,23]. Modifications of intact proteins likely reduce the protease activity, as plasmin digests lactosylated α- and κ-casein less efficiently while β-casein is still cleaved [24]. However, the presented data do not provide conclusive data on this aspect, as the numbers of unmodified α-casein peptides were similar among all samples despite increasing numbers of lactosylated peptides and the possibility of peptides being modified after proteolysis. 

The numbers of hexose-derived peptides increased in the same order as observed for lactosylation being highest in IF, which corresponds well to a previous proteomics study on hexosylation in milk and IF samples [20]. Additionally, most lactosylation and hexosylation sites were identical [18,19,20,21,23] with only three novel sites reported here for the first time, i.e., Lys21 of α_S2_-casein, Lys4 of GlyCAM-1, and Lys74 of LPO. Noteworthy, only two oxidation products were detected, i.e., reactive carbonyls at Pro160 of BT and Thr38 of α_S2_-casein, which has not been reported previously. While oxidized Pro160 was detected in one peptide present in all milk types, oxidized Thr38 was present in several peptides, but their number was the lowest in IF. Interestingly, no AGEs were identified in the peptidome, although formylated and carboxymethylated lysine residues were identified as major AGE-modifications in milk proteomic studies [22,31,32]. It remains open whether AGEs and carbonylation sites were missed due to their low abundances. This could be evaluated at least for carbonylated peptides by enriching them after derivatization using biotin-avidin affinity chromatography [29]. 

Interestingly, many α_S1_-casein- and GlyCAM-1-derived modified peptides originated from areas already covered by high numbers of unmodified peptides, e.g., Val25 to Val37 in α_S1_-casein and Ser54 to Lys73 in GlyCAM-1. However, α_S2_-casein peptides oxidized at Thr38 were not represented by any of the identified unmodified peptides (Figure 5). Noteworthy, this modification site was identified at the protein level in flavored milk drinks [32]. Most modified peptides corresponded to region Lys150 and Lys165 containing four out of eight modified Lys residues. This region was most affected during processing as the numbers of modified peptides significantly increased from RM to UHT milk and further to IF. 

In general, the identified peptides followed the same trends as reported at the protein level with increasing numbers of glycated peptides along the processing chain. Furthermore, proteins followed different trends for the location of modified peptides within the protein sequence. It remains open if peptides get modified before or after proteolytic release, but most likely the modifications will occur at both levels.

## 5. Conclusions

This study analyzed changes in the native peptidomes of raw bovine milk, UHT milk, and IF as well as non-enzymatic modifications present therein. Independent of the milk type, casein-derived peptides were most common. The native peptidomes of RM and its UHT milk appeared to be very similar, whereas IF contained significantly more β-casein-derived peptides, probably due to the addition of sweet whey during its production. To study the effects of thermal processing on the peptidome, in total 26 PTMs were targeted at the peptide level, as increasing degrees of non-enzymatic modifications are well known from proteome studies. To the best of our knowledge, this is the first study targeting so many different PTMs related to glycation, AGE-formation, and oxidation/carbonylation in the peptidomes of RM, UHT milk, and IF. Although many of these modifications were reported in milk proteins, only four types were identified, i.e., lactosylation as the most dominant, followed by hexosylation as well as proline and threonine oxidation. The numbers of lactosylated peptides increased from RM to UHT milk and further to IF as reported before for protein-bound modifications. Thus, the native milk peptidome is affected by diverse chemical reactions including Maillard reactions occurring during processing and storage. Future studies should evaluate quantitative changes in the peptidome induced along the processing chain of bovine milk, especially focusing on known bioactive peptides as well as the effects of the identified modifications on their functional properties. 

## Supplementary Materials

The following are available online at https://www.mdpi.com/2304-8158/9/12/1841/s1, Figure S1: Identification strategy; Figure S2: Overall number of peptides and peptides present in all samples by protein; Figure S3: Protein sequence coverage of α_S1_-casein; Figure S4: Protein sequence coverage of α_S2_-casein; Figure S5: Protein sequence coverage of GlyCAM-1; Figure S6: Protein sequence coverage of κ-casein; Figure S7: Protein sequence coverage containing modified peptides for α_S1_-casein; Figure S8: Protein sequence coverage containing modified peptides for GlyCAM-1; Table S1: List of targeted dynamic modifications; Table S2: List of unmodified peptides sorted by proteins; Table S3: List of modified peptides sorted by proteins.

## References

[1] Meisel H. (2005). Biochemical properties of peptides encrypted in bovine milk proteins. Curr. Med. Chem..

[2] Nagpal R., Behare P., Rana R., Kumar A., Kumar M., Arora S., Morotta F., Jain S., Yadav H. (2011). Bioactive peptides derived from milk proteins and their health beneficial potentials: An update. Food Funct..

[3] Bastian E.D., Brown R.J. (1996). Plasmin in milk and dairy products: An update. Int. Dairy J..

[4] O’Mahony J.A., Fox P.F., Kelly A.L., McSweeney P.L.H., Fox P.F. (2013). Indigenous Enzymes of Milk. Advanced Dairy Chemistry: Volume 1A: Proteins: Basic Aspects.

[5] Dallas D.C., Murray N.M., Gan J. (2015). Proteolytic Systems in Milk: Perspectives on the Evolutionary Function within the Mammary Gland and the Infant. J. Mammary Gland Biol. Neoplasia.

[6] Kelly A.L., O’Flaherty F., Fox P.F. (2006). Indigenous proteolytic enzymes in milk: A brief overview of the present state of knowledge. Int. Dairy J..

[7] Baum F., Fedorova M., Ebner J., Hoffmann R., Pischetsrieder M. (2013). Analysis of the endogenous peptide profile of milk: Identification of 248 mainly casein-derived peptides. J. Proteome Res..

[8] Dallas D.C., Guerrero A., Parker E.A., Garay L.A., Bhandari A., Lebrilla C.B., Barile D., German J.B. (2014). Peptidomic profile of milk of Holstein cows at peak lactation. J. Agric. Food Chem..

[9] Mansor R., Mullen W., Albalat A., Zerefos P., Mischak H., Barrett D.C., Biggs A., Eckersall P.D. (2013). A peptidomic approach to biomarker discovery for bovine mastitis. J. Proteom..

[10] Guerrero A., Dallas D.C., Contreras S., Bhandari A., Cánovas A., Islas-Trejo A., Medrano J.F., Parker E.A., Wang M., Hettinga K. (2015). Peptidomic analysis of healthy and subclinically mastitic bovine milk. Int. Dairy J..

[11] Dallas D.C., Weinborn V., de Moura Bell J.M.L.N., Wang M., Parker E.A., Guerrero A., Hettinga K.A., Lebrilla C.B., German J.B., Barile D. (2014). Comprehensive peptidomic and glycomic evaluation reveals that sweet whey permeate from colostrum is a source of milk protein-derived peptides and oligosaccharides. Food Res. Int..

[12] Andrews A.T. (1983). Proteinases in normal bovine milk and their action on caseins. J. Dairy Res..

[13] Lu R., Stevenson C.D., Guck S.E., Pillsbury L.A., Ismail B., Hayes K.D. (2009). Effect of various heat treatments on plasminogen activation in bovine milk during refrigerated storage. Int. J. Food Sci. Technol..

[14] Hayes M.G., Hurley M.J., Larsen L.B., Heegaard C.W., Magboul A.A., Oliveira J.C., McSweeney P.L., Kelly A.L. (2001). Thermal inactivation kinetics of bovine cathepsin D. J. Dairy Res..

[15] Dalabasmaz S., Dittrich D., Kellner I., Drewello T., Pischetsrieder M. (2019). Identification of peptides reflecting the storage of UHT milk by MALDI-TOF-MS peptide profiling. J. Proteom..

[16] Van Boekel M. (1998). Effect of heating on Maillard reactions in milk. Food Chem..

[17] Arena S., Renzone G., Novi G., Paffetti A., Bernardini G., Santucci A., Scaloni A. (2010). Modern proteomic methodologies for the characterization of lactosylation protein targets in milk. Proteomics.

[18] Siciliano R.A., Mazzeo M.F., Arena S., Renzone G., Scaloni A. (2013). Mass spectrometry for the analysis of protein lactosylation in milk products. Food Res. Int..

[19] Milkovska-Stamenova S., Hoffmann R. (2016). Identification and quantification of bovine protein lactosylation sites in different milk products. J. Proteom..

[20] Milkovska-Stamenova S., Hoffmann R. (2016). Hexose-derived glycation sites in processed bovine milk. J. Proteom..

[21] Milkovska-Stamenova S., Hoffmann R. (2017). Influence of storage and heating on protein glycation levels of processed lactose-free and regular bovine milk products. Food Chem..

[22] Wölk M., Milkovska-Stamenova S., Schröter T., Hoffmann R. (2020). Influence of seasonal variation and processing on protein glycation and oxidation in regular and hay milk. Food Chem..

[23] Renzone G., Arena S., Scaloni A. (2015). Proteomic characterization of intermediate and advanced glycation end-products in commercial milk samples. J. Proteom..

[24] Dalsgaard T.K., Nielsen J.H., Larsen L.B. (2007). Proteolysis of milk proteins lactosylated in model systems. Mol. Nutr. Food Res..

[25] Ames J.M. (1998). Applications of the Maillard reaction in the food industry. Food Chem..

[26] Guéraud F., Atalay M., Bresgen N., Cipak A., Eckl P.M., Huc L., Jouanin I., Siems W., Uchida K. (2010). Chemistry and biochemistry of lipid peroxidation products. Free Radic. Res..

[27] Stadtman E.R., Levine R.L. (2000). Protein oxidation. Ann. N. Y. Acad. Sci..

[28] Henle T. (2005). Protein-bound advanced glycation endproducts (AGEs) as bioactive amino acid derivatives in foods. Amino Acids.

[29] Milkovska-Stamenova S., Mnatsakanyan R., Hoffmann R. (2017). Protein carbonylation sites in bovine raw milk and processed milk products. Food Chem..

[30] Arena S., Renzone G., D’Ambrosio C., Salzano A.M., Scaloni A. (2017). Dairy products and the Maillard reaction: A promising future for extensive food characterization by integrated proteomics studies. Food Chem..

[31] Milkovska-Stamenova S., Hoffmann R. (2019). Diversity of advanced glycation end products in the bovine milk proteome. Amino Acids.

[32] Wölk M., Schröter T., Hoffmann R., Milkovska-Stamenova S. (2020). Profiling of Low-Molecular-Weight Carbonyls and Protein Modifications in Flavored Milk. Antioxidants.

[33] Dallas D., Nielsen S.D. (2018). Milk Peptidomics to Identify Functional Peptides and for Quality Control of Dairy Products. Methods Mol. Biol..

[34] Sassi M., Arena S., Scaloni A. (2015). MALDI-TOF-MS Platform for Integrated Proteomic and Peptidomic Profiling of Milk Samples Allows Rapid Detection of Food Adulterations. J. Agric. Food Chem..

[35] Huppertz T., Fox P.F., Kelly A.L., Yada R.Y. (2018). The caseins: Structure, stability, and functionality. Proteins in Food Processing.

[36] Hurley M., Larsen L., Kelly A., McSweeney P. (2000). The milk acid proteinase cathepsin D: A review. Int. Dairy J..

[37] Ali E., Nielsen S.D., Abd-El Aal S., El-Leboudy A., Saleh E., LaPointe G. (2019). Use of Mass Spectrometry to Profile Peptides in Whey Protein Isolate Medium Fermented by Lactobacillus helveticus LH-2 and Lactobacillus acidophilus La-5. Front. Nutr..

[38] Jost R., Maire J.-C., Maynard F., Secretin M.-C. (1999). Aspects of whey protein usage in infant nutrition, a brief review. Int. J. Food Sci Tech..

[39] Bonnaillie L.M., Tomasula P.M., Onwulata C., Huth P.J. (2008). Whey Protein Fractionation. Whey Processing, Functionality and Health Benefits.

[40] Su M.-Y., Broadhurst M., Liu C.-P., Gathercole J., Cheng W.-L., Qi X.-Y., Clerens S., Dyer J.M., Day L., Haigh B. (2017). Comparative analysis of human milk and infant formula derived peptides following in vitro digestion. Food Chem..

[41] Benkerroum N. (2010). Antimicrobial peptides generated from milk proteins: A survey and prospects for application in the food industry. A review. Int. J. Dairy Technol..

[42] Meltretter J., Wüst J., Pischetsrieder M. (2013). Comprehensive analysis of nonenzymatic post-translational β-lactoglobulin modifications in processed milk by ultrahigh-performance liquid chromatography-tandem mass spectrometry. J. Agric. Food Chem..

[43] Wada Y., Lönnerdal B. (2014). Effects of different industrial heating processes of milk on site-specific protein modifications and their relationship to in vitro and in vivo digestibility. J. Agric. Food Chem..

