# Supplementary material for: Comprehensive Profiling of the Native and Modified Peptidomes of Raw Bovine Milk and Processed Milk Products
Source: Foods. 2020 Dec 10;9(12):1841. doi: 10.3390/foods9121841 (PMC7763055; doi:10.3390/foods9121841)
Supplement: Supplementary file 1 [file foods-09-01841-s001.pdf]

**Comprehensive profiling of the native and modified peptidomes of raw bovine milk and processed milk products**

Michele Wölk[1,2], Sanja Milkovska-Stamenova[1,2*] and Ralf Hoffmann[1,2]

[1] Institute of Bioanalytical Chemistry, Faculty of Chemistry and Mineralogy, Universität Leipzig, 04103 Leipzig, Germany
[2] Center for Biotechnology and Biomedicine, Universität Leipzig, 04103 Leipzig, Germany
* Correspondence: sanja.milkovska@uni-leipzig.de

## Supporting information

**Table S1:** List of targeted dynamic modifications 2

**Table S2:**.List of unmodified peptides sorted by proteins 3

**Table S3:** List of modified peptides sorted by proteins 33

**Figure S1:** Identification strategy 40

**Figure S2:**.Overall number of peptides and peptides present in all samples by protein 41

**Figure S3:** Protein sequence coverage of $\alpha_{S1}$-casein 42

**Figure S4:** Protein sequence coverage of $\alpha_{S2}$-casein 43

**Figure S5:** Protein sequence coverage of GlyCAM-1 44

**Figure S6:** Protein sequence coverage of κ-casein 45

**Figure S7:** Protein sequence coverage containing modified peptides for $\alpha_{S1}$-casein 46

**Figure S8:** Protein sequence coverage containing modified peptides for GlyCAM-1 47

**Table S1:** List of targeted dynamic modifications corresponding to Amadori products (red), advanced glycation end-products (green), and oxidation/carbonylation (blue).

| Modification | Mass shift [Da] | Amino acid residue |
|---|---|---|
| Acetylation[c] | +42.011 | K |
| Acrolein[c] | +56.026 | C, H, K |
| Argpyrimidine[e] | +80.026 | R |
| Carboxyethyl[f] | +72.021 | K, R |
| Carboxymethyl[a] | +58.005 | K, R |
| Crotonaldehyde[c] | +70.042 | C, H, K |
| Formylation[a] | +27.995 | K |
| Glutamic semialdehyde[b] | -43.053 | R |
| Glyceraldehyde-derived pyridinium[f] | +109.029 | K |
| Glycerinyl lysine[c] | +88.016 | K |
| Glyoxal-derived hydroimidazolium[e] | +39.995 | R |
| Hexosylation[a] (Hex) | +162.053 | K |
| Lactosylation[a] (Lac) | +324.106 | K |
| Methylglyoxal-derived hydroimidazolones[e] | +54.011 | R |
| Oxalic acid monolysinylamide[f] | +71.985 | K |
| Oxidation[b] (Ox) | +15.995 | P, C, W, Y |
| Pentenal[c] | +84.060 | C, H, K |
| Pyrraline[f] | +108.021 | K |
| Tetrahydropyrimidine[e] | +144.042 | R |
| +14 Da Carbonylation [b] | +13.979 | A, E, I, L, K, Q, V |
| 2-Amino-3-ketobutyric acid[b] (T(Ox)) | -2.016 | T |
| 2-Aminoadipic semialdehyde[b] | -1.032 | K |
| 4-Hydroxy-2-hexanal[d] | +114.068 | C, H, K |
| 4-Hydroxy-2-nonenal[d] | +156.115 | C, H, K |
| 4-Oxo-2-hexanal[d] | +112.052 | C, H, K |
| 4-Oxo-2-nonenal[d] | +154.099 | C, H, K |

[a-f] denote the templates in which modifications were searched together within Proteome Discoverer. Each template additionally contained oxidation of methionine and phosphorylation of serine as dynamic modifications. As PEAKS allows targeting many PTMs, all modifications were included as dynamic modifications.

**Table S2:** Unmodified peptides identified by both Proteome Discoverer 2.2 (Xcorr) and PEAKS Studio 10.5 (-10lgP) sorted by proteins (if present signal peptides were removed). An "x" indicates that the peptides was present in RM, UHT milk or IF, whereas a "blank" indicates that it was not detected. Methionine oxidation (M(Ox)) and phosphorylation of serine* (S(Phospho)) were considered as variable modifications.

| Nr. | Start pos. | End pos. | Sequence | Modification | z | *m/z* | $t_R$ (min) | Xcorr | -10lgP | RM[a] | UHT[a] | IF[a] | Literature (milk[1-6], colostrum[7]) |
|---|---|---|---|---|---|---|---|---|---|---|---|---|---|
| **Alpha-S1-casein (P02662)** | | | | | | | | | | | | | |
| 1 | 1 | 21 | RPKHPIKHQGLPQEVLNENLL | none | 3 (4) | 820.799 | 38.94 | 3.8 | 57.7 | x | x | x | 1; 3; 5 |
| 2 | 1 | 22 | RPKHPIKHQGLPQEVLNENLLR | none | 4 (5) | 654.878 | 35.38 | 3.5 | 44.8 | x | x | x | 1; 3; 5 |
| 3 | 4 | 19 | HPIKHQGLPQEVLNEN | none | 3 | 618.325 | 27.28 | 3.3 | 36.2 | x | x | x | 1; 7; 5 |
| 4 | 4 | 21 | HPIKHQGLPQEVLNENLL | none | 3 (2,4) | 693.717 | 43.32 | 3.4 | 68.6 | x | x | x | 1; 3; 5 |
| 5 | 4 | 22 | HPIKHQGLPQEVLNENLLR | none | 3 (4) | 745.749 | 39.00 | 5.0 | 59.4 | x | x | x | 1; 3; 5 |
| 6 | 4 | 23 | HPIKHQGLPQEVLNENLLRF | none | 3 | 794.773 | 48.95 | 5.0 | 54.4 | x | x | | 5 |
| 7 | 7 | 16 | KHQGLPQEVL | none | 2 | 574.824 | 29.04 | 2.5 | 56.9 | x | x | x | |
| 8 | 7 | 23 | KHQGLPQEVLNENLLRF | none | 3 | 679.041 | 51.10 | 3.3 | 58.3 | | | x | |
| 9 | 8 | 21 | HQGLPQEVLNENLL | none | 2 | 802.425 | 49.46 | 2.9 | 58.9 | x | x | x | 1; 5 |
| 10 | 8 | 22 | HQGLPQEVLNENLLR | none | 3 (2) | 587.320 | 44.19 | 4.7 | 92.6 | x | x | x | 1; 3; 5 |
| 11 | 9 | 17 | QGLPQEVLN | none | 2 | 499.269 | 35.17 | 1.7 | 43.1 | | | x | |
| 12 | 9 | 21 | QGLPQEVLNENLL | none | 2 | 733.897 | 55.28 | 3.4 | 53.5 | | | x | 5 |
| 13 | 9 | 22 | QGLPQEVLNENLLR | none | 2 | 811.948 | 49.07 | 3.4 | 74.6 | x | x | x | 5 |
| 14 | 9 | 23 | QGLPQEVLNENLLRF | none | 2 | 885.482 | 59.94 | 3.3 | 77.2 | | | x | |
| 15 | 10 | 17 | GLPQEVLN | none | 2 | 435.239 | 34.78 | 1.9 | 46.4 | x | x | x | 1 |
| 16 | 10 | 18 | GLPQEVLNE | none | 2 | 499.761 | 38.33 | 2.2 | 46.7 | | | x | |
| 17 | 10 | 20 | GLPQEVLNENL | none | 2 | 613.325 | 47.92 | 2.0 | 38.0 | x | x | x | 5; 7 |
| 18 | 10 | 21 | GLPQEVLNENLL | none | 2 | 669.867 | 55.75 | 2.8 | 45.7 | x | x | x | 1; 3; 5 |
| 19 | 10 | 22 | GLPQEVLNENLLR | none | 2 | 747.917 | 49.28 | 4.3 | 74.3 | x | x | x | 1; 3; 5; 7 |
| 20 | 10 | 23 | GLPQEVLNENLLRF | none | 2 | 821.451 | 60.49 | 4.0 | 75.2 | x | x | x | 1; 5 |
| 21 | 10 | 24 | GLPQEVLNENLLRFF | none | 2 | 894.988 | 70.69 | 3.9 | 55.5 | | | x | |
| 22 | 11 | 20 | LPQEVLNENL | none | 2 | 584.813 | 42.74 | 2.6 | 50.3 | x | x | | 1 |
| 23 | 11 | 21 | LPQEVLNENLL | none | 2 | 641.357 | 51.48 | 2.2 | 42.9 | x | x | x | 1; 3; 5 |
| 24 | 11 | 22 | LPQEVLNENLLR | none | 2 | 719.407 | 44.47 | 3.8 | 68.1 | x | x | x | |

| Nr. | Start pos. | End pos. | Sequence | Modification | z | *m/z* | $t_R$ (min) | Xcorr | -10lgP | RM[a] | UHT[a] | IF[a] | Literature (milk[1-6], colostrum[7]) |
|---|---|---|---|---|---|---|---|---|---|---|---|---|---|
| 25 | 11 | 23 | LPQEVLNENLLRF | none | 2 (3) | 792.941 | 56.68 | 3.7 | 70.7 | x | x | x | 1; 3; 5; 7 |
| 26 | 13 | 22 | QEVLNENLLR | none | 2 | 614.337 | 37.50 | 2.6 | 48.7 | x | x | x | 1; 3; 5 |
| 27 | 13 | 23 | QEVLNENLLRF | none | 2 | 687.873 | 51.93 | 2.7 | 56.3 | | | x | 5 |
| 28 | 14 | 21 | EVLNENLL | none | 2 | 472.258 | 44.55 | 2.2 | 39.6 | | | x | 1 |
| 29 | 14 | 22 | EVLNENLLR | none | 2 | 550.309 | 36.43 | 2.4 | 50.8 | x | x | x | 1; 4 |
| 30 | 14 | 23 | EVLNENLLRF | none | 2 | 623.844 | 51.49 | 2.7 | 53.6 | x | x | x | 1; 5; 7 |
| 31 | 14 | 24 | EVLNENLLRFF | none | 2 | 697.379 | 63.20 | 2.8 | 43.5 | | | x | |
| 32 | 15 | 22 | VLNENLLR | none | 2 | 485.786 | 32.27 | 3.1 | 50.2 | x | x | x | 1 |
| 33 | 15 | 23 | VLNENLLRF | none | 2 | 559.321 | 48.86 | 2.8 | 55.9 | x | x | x | 1; 5 |
| 34 | 16 | 22 | LNENLLR | none | 2 | 436.252 | 28.15 | 2.6 | 38.8 | x | x | | 1 |
| 35 | 16 | 23 | LNENLLRF | none | 2 | 509.787 | 46.08 | 3.2 | 49.0 | x | x | x | 1; 5 |
| 36 | 16 | 24 | LNENLLRFF | none | 2 | 583.321 | 58.93 | 2.9 | 51.5 | x | x | x | 5 |
| 37 | 17 | 23 | NENLLRF | none | 2 | 453.244 | 40.95 | 2.4 | 39.8 | x | x | x | 1; 4 |
| 38 | 17 | 24 | NENLLRFF | none | 2 | 526.779 | 55.49 | 2.9 | 46.7 | x | x | x | 1; 5 |
| 39 | 23 | 33 | FFVAPFPEVFG | none | 2 | 628.821 | 70.01 | 1.9 | 57.0 | x | x | | 1; 5 |
| 40 | 23 | 34 | FFVAPFPEVFGK | none | 2 | 692.869 | 60.89 | 2.8 | 67.1 | x | x | x | 1; 3; 5 |
| 41 | 23 | 35 | FFVAPFPEVFGKE | none | 2 | 757.390 | 61.19 | 3.4 | 76.5 | x | x | | 5 |
| 42 | 23 | 36 | FFVAPFPEVFGKEK | none | 2 (3) | 821.437 | 55.07 | 3.1 | 73.0 | x | x | x | 1; 3; 5 |
| 43 | 23 | 37 | FFVAPFPEVFGKEKV | none | 2 (3) | 870.972 | 57.29 | 3.3 | 75.3 | x | x | x | 5 |
| 44 | 23 | 39 | FFVAPFPEVFGKEKVNE | none | 2 (3) | 992.515 | 55.58 | 3.9 | 85.0 | x | x | x | 1; 3; 5 |
| 45 | 23 | 41 | FFVAPFPEVFGKEKVNELS | none | 2 (3) | 1092.573 | 58.00 | 3.2 | 84.2 | x | x | x | 5 |
| 46 | 23 | 42 | FFVAPFPEVFGKEKVNELSK | none | 4 (3) | 578.813 | 53.57 | 1.8 | 40.2 | x | x | x | 5 |
| 47 | 24 | 32 | FVAPFPEVF | none | 2 | 526.775 | 63.22 | 2.4 | 51.6 | x | x | x | 1; 3; 5 |
| 48 | 24 | 33 | FVAPFPEVFG | none | 2 | 555.286 | 62.41 | 2.6 | 68.8 | x | x | x | 1; 5 |
| 49 | 24 | 34 | FVAPFPEVFGK | none | 2 | 619.334 | 53.66 | 2.5 | 56.7 | x | x | x | 1; 3; 5; 7 |
| 50 | 24 | 35 | FVAPFPEVFGKE | none | 2 | 683.854 | 54.21 | 2.7 | 72.1 | x | x | x | 5 |
| 51 | 24 | 36 | FVAPFPEVFGKEK | none | 2 (3) | 747.903 | 48.16 | 3.0 | 76.5 | x | x | x | 1; 5 |
| 52 | 24 | 37 | FVAPFPEVFGKEKV | none | 2 (3) | 797.437 | 51.04 | 3.5 | 73.2 | x | x | x | 1; 2; 5; 7 |
| 53 | 24 | 41 | FVAPFPEVFGKEKVNELS | none | 2 (3) | 1019.038 | 52.38 | 3.3 | 73.9 | x | x | x | |

| Nr. | Start pos. | End pos. | Sequence | Modification | z | *m/z* | $t_R$ (min) | Xcorr | -10lgP | RM[a] | UH T[a] | IF[a] | Literature (milk[1-6], colostrum[7]) |
|---|---|---|---|---|---|---|---|---|---|---|---|---|---|
| 54 | 25 | 32 | VAPFPEVF | none | 2 | 453.241 | 55.28 | 1.8 | 46.5 | x | x | x | 1; 5 |
| 55 | 25 | 33 | VAPFPEVFG | none | 2 | 481.751 | 54.34 | 2.5 | 61.2 | x | x | x | 1; 5 |
| 56 | 25 | 34 | VAPFPEVFGK | none | 2 | 545.799 | 45.43 | 1.4 | 50.7 | x | x | x | 1; 5 |
| 57 | 25 | 35 | VAPFPEVFGKE | none | 2 | 610.322 | 46.21 | 2.1 | 58.8 | x | x | x | 1; 5; 7 |
| 58 | 25 | 36 | VAPFPEVFGKEK | none | 2 | 674.368 | 39.75 | 2.0 | 72.9 | x | x | x | 2; 5 |
| 59 | 25 | 37 | VAPFPEVFGKEKV | none | 2 (3) | 723.903 | 43.89 | 2.9 | 73.1 | x | x | x | 1; 2; 5 |
| 60 | 25 | 41 | VAPFPEVFGKEKVNELS | none | 2 | 945.505 | 46.15 | 2.5 | 70.8 | x | x | x | |
| 61 | 26 | 33 | APFPEVFG | none | 2 | 432.218 | 51.00 | 1.5 | 40.1 | x | x | | 1; 3; 5 |
| 62 | 26 | 34 | APFPEVFGK | none | 2 | 496.265 | 42.16 | 2.7 | 66.3 | x | x | x | 1 |
| 63 | 26 | 35 | APFPEVFGKE | none | 2 | 560.786 | 42.90 | 3.2 | 77.3 | x | x | x | 1; 3; 5 |
| 64 | 26 | 36 | APFPEVFGKEK | none | 2 (3) | 624.835 | 36.41 | 2.8 | 71.3 | x | x | x | 1; 3; 5 |
| 65 | 26 | 37 | APFPEVFGKEKV | none | 2 (3) | 674.368 | 40.86 | 3.5 | 82.8 | x | x | x | 1; 5 |
| 66 | 26 | 39 | APFPEVFGKEKVNE | none | 3 (2) | 530.944 | 38.68 | 3.0 | 68.8 | x | x | x | 1; 5 |
| 67 | 26 | 40 | APFPEVFGKEKVNEL | none | 3 | 568.637 | 46.12 | 2.7 | 47.5 | x | x | x | |
| 68 | 26 | 41 | APFPEVFGKEKVNELS | none | 2 (3) | 895.971 | 43.69 | 2.5 | 80.6 | x | x | x | 1 |
| 69 | 27 | 34 | PFPEVFGK | none | 2 | 460.747 | 45.54 | 2.1 | 51.0 | x | x | x | |
| 70 | 27 | 36 | PFPEVFGKEK | none | 2 | 589.315 | 39.85 | 3.0 | 61.5 | x | x | x | |
| 71 | 27 | 37 | PFPEVFGKEKV | none | 3 (2) | 426.236 | 47.50 | 2.1 | 40.3 | x | x | x | 2; 5 |
| 72 | 28 | 34 | FPEVFGK | none | 2 | 412.221 | 36.92 | 2.4 | 55.6 | x | x | x | |
| 73 | 28 | 36 | FPEVFGKEK | none | 2 | 540.788 | 31.18 | 2.5 | 53.6 | x | x | x | 1 |
| 74 | 28 | 37 | FPEVFGKEKV | none | 2 | 590.323 | 36.71 | 2.9 | 61.2 | x | x | x | 1; 5 |
| 75 | 35 | 58 | EKVNELSKDIGSESTEDQAMEDIK | none | 3 (4) | 899.092 | 36.84 | 6.8 | 99.4 | x | x | x | |
| 76 | 35 | 58 | EKVNELSKDIGSESTEDQAmEDIK | M20(Ox) | 3 (4) | 904.427 | 31.78 | 7.2 | 83.1 | x | x | x | |
| 77 | 35 | 58 | EKVNELSKDIGSEsTEDQAMEDIK | S14(Phospho) | 3 | 925.750 | 38.67 | 6.2 | 55.2 | x | x | x | 5 |
| 78 | 37 | 58 | VNELSKDIGSESTEDQAMEDIK | none | 3 | 813.379 | 38.03 | 5.6 | 85.0 | x | x | x | 5 |
| 79 | 37 | 58 | VNELSKDIGSESTEDQAmEDIK | M18(Ox) | 3 | 818.711 | 32.25 | 6.0 | 81.1 | x | x | x | |
| 80 | 37 | 58 | VNELSKDIGSEsTEDQAMEDIK | S12(Phospho) | 3 | 840.034 | 40.51 | 6.9 | 69.5 | x | x | x | |
| 81 | 38 | 53 | NELSKDIGSESTEDQA | none | 2 | 861.887 | 26.89 | 4.6 | 80.3 | x | x | x | 5 |
| 82 | 43 | 58 | DIGSESTEDQAMEDIK | none | 2 | 884.384 | 34.89 | 4.9 | 99.3 | x | x | x | |

| Nr. | Start pos. | End pos. | Sequence | Modification | z | *m/z* | $t_R$ (min) | Xcorr | -10lgP | RM[a] | UHT[a] | IF[a] | Literature (milk[1-6], colostrum[7]) |
|---|---|---|---|---|---|---|---|---|---|---|---|---|---|
| 83 | 43 | 58 | DIGSESTEDQAmEDIK | M12(Ox) | 2 | 892.378 | 27.47 | 5.0 | 94.7 | x | x | | |
| 84 | 43 | 58 | DIGSEsTEDQAMEDIK | S6(Phospho) | 2 | 924.367 | 38.89 | 4.8 | 74.7 | x | x | x | |
| 85 | 44 | 58 | IGSESTEDQAmEDIK | M11(Ox) | 2 | 834.865 | 24.02 | 3.4 | 59.9 | x | x | | |
| 86 | 69 | 79 | EEIVPNsVEQK | S7(Phospho) | 2 | 676.311 | 26.67 | 2.5 | 39.0 | | | x | |
| 87 | 80 | 90 | HIQKEDVPSER | none | 3 (2) | 669.342 | 13.87 | 4.1 | 54.9 | x | x | x | 1; 3; 4; 5 |
| 88 | 80 | 91 | HIQKEDVPSERY | none | 2 (3) | 750.875 | 19.18 | 3.1 | 63.8 | x | x | x | 1; 3; 5 |
| 89 | 80 | 92 | HIQKEDVPSERYL | none | 3 (2) | 538.614 | 27.07 | 3.5 | 74.9 | x | x | x | 1; 5 |
| 90 | 80 | 93 | HIQKEDVPSERYLG | none | 3 (2) | 557.621 | 25.35 | 4.5 | 61.2 | x | x | x | 1; 3; 5 |
| 91 | 80 | 94 | HIQKEDVPSERYLGY | none | 3 | 611.975 | 30.72 | 4.5 | 76.5 | x | x | x | 1 |
| 92 | 80 | 95 | HIQKEDVPSERYLGYL | none | 3 | 649.672 | 40.11 | 3.6 | 51.9 | x | | x | |
| 93 | 80 | 96 | HIQKEDVPSERYLGYLE | none | 3 | 692.686 | 38.90 | 3.8 | 54.7 | x | | x | |
| 94 | 80 | 97 | HIQKEDVPSERYLGYLEQ | none | 3 | 735.371 | 38.91 | 4.1 | 62.9 | x | x | x | 1 |
| 95 | 80 | 98 | HIQKEDVPSERYLGYLEQL | none | 3 | 773.065 | 50.14 | 4.1 | 58.0 | x | x | x | 1 |
| 96 | 80 | 99 | HIQKEDVPSERYLGYLEQLL | none | 2 (3) | 1215.638 | 61.48 | 4.0 | 71.3 | x | x | x | 5 |
| 97 | 80 | 100 | HIQKEDVPSERYLGYLEQLLR | none | 4 (3,5) | 647.346 | 58.44 | 4.5 | 57.3 | x | x | x | 1; 3; 5 |
| 98 | 80 | 101 | HIQKEDVPSERYLGYLEQLLRL | none | 4 (3) | 675.618 | 67.03 | 3.9 | 48.5 | x | x | x | |
| 99 | 80 | 102 | HIQKEDVPSERYLGYLEQLLRLK | none | 5 (3,4) | 566.314 | 60.64 | 4.5 | 57.2 | x | x | x | 1; 3; 5 |
| 100 | 80 | 103 | HIQKEDVPSERYLGYLEQLLRLKK | none | 3 (4,5,6) | 985.888 | 56.22 | 3.2 | 28.2 | x | x | x | 1 |
| 101 | 80 | 104 | HIQKEDVPSERYLGYLEQLLRLKKY | none | 5 | 624.547 | 57.25 | 4.0 | 39.7 | x | x | x | |
| 102 | 81 | 89 | IQKEDVPSE | none | 2 | 522.762 | 17.04 | 2.2 | 36.3 | x | x | x | 1; 3; 5 |
| 103 | 81 | 90 | IQKEDVPSER | none | 2 (3) | 400.878 | 15.15 | 2.9 | 54.6 | x | x | x | 1; 3; 5 |
| 104 | 81 | 91 | IQKEDVPSERY | none | 2 | 682.345 | 21.29 | 2.7 | 50.6 | x | x | x | 5 |
| 105 | 81 | 92 | IQKEDVPSERYL | none | 3 | 492.927 | 29.90 | 3.1 | 50.6 | x | x | x | 1 |
| 106 | 81 | 93 | IQKEDVPSERYLG | none | 3 | 511.934 | 28.05 | 2.8 | 37.5 | x | x | x | 1 |
| 107 | 82 | 93 | QKEDVPSERYLG | none | 3 (2) | 474.240 | 25.15 | 2.6 | 27.4 | x | x | | 1 |
| 108 | 83 | 91 | KEDVPSERY | none | 2 | 561.773 | 17.22 | 3.1 | 46.1 | x | x | x | 1; 5 |
| 109 | 83 | 93 | KEDVPSERYLG | none | 2 | 646.827 | 24.93 | 2.8 | 54.4 | x | x | | 1 |
| 110 | 84 | 91 | EDVPSERY | none | 2 | 497.727 | 20.58 | 1.4 | 46.9 | x | x | x | |
| 111 | 86 | 97 | VPSERYLGYLEQ | none | 2 | 727.370 | 40.77 | 2.2 | 47.0 | x | x | x | 1 |

| Nr. | Start pos. | End pos. | Sequence | Modification | z | *m/z* | $t_R$ (min) | Xcorr | -10lgP | RM[a] | UHT[a] | IF[a] | Literature (milk[1-6], colostrum[7]) |
|---|---|---|---|---|---|---|---|---|---|---|---|---|---|
| 112 | 86 | 98 | VPSERYLGYLEQL | none | 2 | 783.911 | 53.16 | 2.7 | 58.0 | x | x | | 1 |
| 113 | 86 | 99 | VPSERYLGYLEQLL | none | 2 | 840.453 | 65.13 | 2.6 | 51.1 | x | x | x | 1 |
| 114 | 91 | 99 | YLGYLEQLL | none | 2 | 556.304 | 64.78 | 1.5 | 24.2 | x | x | | 5 |
| 115 | 91 | 100 | YLGYLEQLLR | none | 2 | 634.355 | 58.27 | 3.3 | 67.7 | x | x | x | |
| 116 | 91 | 102 | YLGYLEQLLRLK | none | 2 (3) | 754.945 | 60.51 | 3.8 | 68.5 | x | x | x | |
| 117 | 91 | 103 | YLGYLEQLLRLKK | none | 3 (4) | 546.330 | 54.07 | 2.5 | 36.5 | x | x | | |
| 118 | 92 | 100 | LGYLEQLLR | none | 2 | 552.823 | 51.92 | 3.2 | 55.6 | x | x | x | 1; 3; 5 |
| 119 | 92 | 102 | LGYLEQLLRLK | none | 3 | 449.278 | 55.65 | 3.0 | 56.8 | x | x | | |
| 120 | 93 | 99 | GYLEQLL | none | 2 | 418.231 | 53.87 | 1.8 | 34.9 | x | x | | |
| 121 | 93 | 100 | GYLEQLLR | none | 2 | 496.281 | 46.78 | 2.6 | 50.9 | x | x | x | 1 |
| 122 | 93 | 101 | GYLEQLLRL | none | 2 | 552.823 | 61.34 | 2.1 | 44.7 | x | x | | 5 |
| 123 | 93 | 102 | GYLEQLLRLK | none | 2 (3) | 616.871 | 51.98 | 3.4 | 58.7 | x | x | x | 5 |
| 124 | 94 | 100 | YLEQLLR | none | 2 | 467.771 | 39.11 | 2.6 | 39.9 | x | x | x | 1; 5 |
| 125 | 94 | 101 | YLEQLLRL | none | 2 | 524.312 | 54.57 | 2.5 | 38.1 | x | x | | 1 |
| 126 | 94 | 102 | YLEQLLRLK | none | 2 | 588.360 | 44.78 | 2.8 | 49.6 | x | x | x | 1 |
| 127 | 95 | 101 | LEQLLRL | none | 2 | 442.782 | 45.15 | 2.3 | 32.2 | x | x | x | 1 |
| 128 | 95 | 102 | LEQLLRLK | none | 2 | 506.828 | 34.31 | 3.0 | 36.8 | x | x | x | 1 |
| 129 | 101 | 123 | LKKYKVPQLEIVPNsAEERLHSM | S15(Phospho) | 3 (4,5) | 930.486 | 43.25 | 5.4 | 62.6 | x | x | x | 5 |
| 130 | 103 | 119 | KYKVPQLEIVPNSAEER | none | 3 | 667.365 | 36.59 | 3.4 | 50.4 | x | x | x | 3; 5 |
| 131 | 103 | 119 | KYKVPQLEIVPNsAEER | S13(Phospho) | 3 (2) | 694.021 | 38.01 | 4.7 | 62.6 | x | x | x | 1; 3; 5 |
| 132 | 103 | 121 | KYKVPQLEIVPNsAEERLH | S13(Phospho) | 4 | 583.304 | 40.08 | 4.4 | 40.6 | x | x | x | |
| 133 | 103 | 122 | KYKVPQLEIVPNsAEERLHS | S13(Phospho) | 4 (3) | 605.061 | 40.30 | 4.3 | 27.0 | x | x | x | |
| 134 | 103 | 123 | KYKVPQLEIVPNSAEERLHSM | none | 3 (4) | 823.437 | 42.87 | 5.0 | 56.7 | x | x | x | 5 |
| 135 | 103 | 123 | KYKVPQLEIVPNSAEERLHSm | M21(Ox) | 4 | 621.827 | 38.29 | 4.3 | 42.2 | x | x | x | |
| 136 | 103 | 123 | KYKVPQLEIVPNsAEERLHSM | S13(Phospho) | 3 (2,4) | 855.424 | 44.86 | 5.5 | 56.3 | x | x | x | 5 |
| 137 | 103 | 124 | KYKVPQLEIVPNsAEERLHSMK | S13(Phospho) | 4 (5) | 669.844 | 40.83 | 4.4 | 23.3 | x | x | x | 3; 5 |
| 138 | 104 | 114 | YKVPQLEIVPN | none | 2 | 650.369 | 43.92 | 2.6 | 69.9 | | | x | |
| 139 | 104 | 119 | YKVPQLEIVPNSAEER | none | 2 (3) | 936.497 | 40.61 | 4.1 | 78.7 | x | x | x | 5 |
| 140 | 104 | 119 | YKVPQLEIVPNsAEER | S12(Phospho) | 2 (3) | 976.481 | 42.23 | 5.6 | 77.4 | x | x | x | 1; 3; 5 |

| Nr. | Start pos. | End pos. | Sequence | Modification | z | *m/z* | $t_R$ (min) | Xcorr | -10lgP | RM[a] | UHT[a] | IF[a] | Literature (milk[1-6], colostrum[7]) |
|---|---|---|---|---|---|---|---|---|---|---|---|---|---|
| 141 | 104 | 122 | YKVPQLEIVPNsAEERLHS | S12(Phospho) | 3 | 763.714 | 43.72 | 4.7 | 48.8 | x | x | x | 5 |
| 142 | 104 | 123 | YKVPQLEIVPNSAEERLHSM | none | 3 (4) | 780.740 | 46.08 | 3.8 | 55.8 | x | x | x | 5 |
| 143 | 104 | 123 | YKVPQLEIVPNSAEERLHSm | M20(Ox) | 3 | 786.071 | 41.64 | 3.7 | 58.3 | x | x | x | 3 |
| 144 | 104 | 123 | YKVPQLEIVPNsAEERLHSM | S12(Phospho) | 3 (2,4) | 807.394 | 48.27 | 4.7 | 56.7 | x | x | x | 1; 3; 5 |
| 145 | 104 | 124 | YKVPQLEIVPNsAEERLHSMK | S12(Phospho) | 3 (4) | 850.091 | 43.88 | 5.0 | 50.6 | x | x | x | 3; 5 |
| 146 | 105 | 114 | KVPQLEIVPN | none | 2 | 568.837 | 38.05 | 1.6 | 46.1 | | | x | |
| 147 | 105 | 119 | KVPQLEIVPNSAEER | none | 3 | 570.312 | 35.01 | 4.3 | 62.0 | x | x | x | 1 |
| 148 | 105 | 119 | KVPQLEIVPNsAEER | S11(Phospho) | 2 (3) | 894.949 | 37.14 | 4.0 | 41.8 | x | x | x | 3; 5 |
| 149 | 105 | 121 | KVPQLEIVPNsAEERLH | S11(Phospho) | 3 | 680.350 | 39.51 | 4.3 | 40.3 | | | x | |
| 150 | 105 | 123 | KVPQLEIVPNSAEERLHSM | none | 3 | 726.384 | 42.75 | 5.2 | 57.1 | x | x | x | 5; 7 |
| 151 | 105 | 123 | KVPQLEIVPNSAEERLHSm | M19(Ox) | 3 | 731.715 | 37.46 | 4.2 | 59.0 | x | x | x | 7 |
| 152 | 105 | 123 | KVPQLEIVPNsAEERLHSM | S11(Phospho) | 3 (2) | 753.040 | 45.20 | 5.5 | 50.4 | x | x | x | |
| 153 | 105 | 124 | KVPQLEIVPNsAEERLHSMK | S11(Phospho) | 4 | 597.057 | 40.50 | 2.9 | 24.5 | x | x | x | |
| 154 | 106 | 119 | VPQLEIVPNSAEER | none | 2 (3) | 790.917 | 38.22 | 3.9 | 86.8 | x | x | x | 1; 3; 5 |
| 155 | 106 | 119 | VPQLEIVPNsAEER | S10(Phospho) | 2 | 830.901 | 40.32 | 4.7 | 67.8 | x | x | x | 1; 3; 5 |
| 156 | 106 | 121 | VPQLEIVPNsAEERLH | S10(Phospho) | 3 (2) | 637.652 | 42.59 | 4.5 | 47.6 | x | x | x | |
| 157 | 106 | 122 | VPQLEIVPNSAEERLHS | none | 3 | 640.007 | 40.51 | 3.5 | 40.5 | x | x | x | 1; 5 |
| 158 | 106 | 122 | VPQLEIVPNsAEERLHS | S10(Phospho) | 2 (3) | 999.488 | 42.66 | 4.7 | 59.0 | x | x | x | |
| 159 | 106 | 123 | VPQLEIVPNSAEERLHSM | none | 3 (2) | 683.685 | 45.68 | 4.8 | 89.8 | x | x | x | 5 |
| 160 | 106 | 123 | VPQLEIVPNSAEERLHSm | M18(Ox) | 3 (2) | 689.017 | 40.08 | 4.9 | 84.4 | x | x | x | |
| 161 | 106 | 123 | VPQLEIVPNsAEERLHSM | S10(Phospho) | 3 (2) | 710.342 | 48.28 | 5.4 | 73.3 | x | x | x | 3; 5 |
| 162 | 106 | 124 | VPQLEIVPNSAEERLHSMK | none | 3 | 726.383 | 41.19 | 3.3 | 37.6 | x | x | x | |
| 163 | 106 | 124 | VPQLEIVPNsAEERLHSMK | S10(Phospho) | 2 (3,4) | 1129.056 | 42.96 | 4.4 | 66.1 | x | x | x | 5 |
| 164 | 106 | 131 | VPQLEIVPNsAEERLHSMKEGIHAQQ | S10(Phospho) | 3 | 1007.494 | 43.59 | 4.8 | 34.5 | x | x | x | |
| 165 | 106 | 132 | VPQLEIVPNsAEERLHSMKEGIHAQQK | S10(Phospho) | 4 (5) | 630.518 | 41.32 | 3.8 | 23.0 | x | x | x | |
| 166 | 108 | 119 | QLEIVPNSAEER | none | 2 | 692.855 | 31.41 | 2.4 | 62.7 | x | x | x | 5 |
| 167 | 108 | 119 | QLEIVPNsAEER | S8(Phospho) | 2 | 732.840 | 33.73 | 2.9 | 54.2 | x | x | x | 1; 3; 5 |
| 168 | 108 | 123 | QLEIVPNSAEERLHSM | none | 3 | 618.312 | 41.19 | 2.9 | 41.1 | x | x | x | |
| 169 | 108 | 123 | QLEIVPNsAEERLHSM | S8(Phospho) | 3 (2) | 644.967 | 44.50 | 2.7 | 28.1 | x | x | x | |

| Nr. | Start pos. | End pos. | Sequence | Modification | z | *m/z* | $t_R$ (min) | Xcorr | -10lgP | RM[a] | UHT[a] | IF[a] | Literature (milk[1-6], colostrum[7]) |
|---|---|---|---|---|---|---|---|---|---|---|---|---|---|
| 170 | 108 | 124 | QLEIVPNsAEERLHSMK | S8(Phospho) | 3 | 687.667 | 39.02 | 2.5 | 23.3 | x | x | x | 5 |
| 171 | 109 | 119 | LEIVPNSAEER | none | 2 | 628.827 | 29.74 | 2.7 | 56.5 | x | x | x | 1; 5 |
| 172 | 109 | 119 | LEIVPNsAEER | S7(Phospho) | 2 | 668.809 | 32.06 | 2.8 | 45.2 | x | x | x | 1; 5 |
| 173 | 109 | 123 | LEIVPNsAEERLHSM | S7(Phospho) | 2 | 902.919 | 43.78 | 3.5 | 57.5 | x | x | | |
| 174 | 110 | 119 | EIVPNSAEER | none | 2 | 572.284 | 20.14 | 1.9 | 40.4 | x | x | x | 1; 5 |
| 175 | 110 | 119 | EIVPNsAEER | S6(Phospho) | 2 | 612.268 | 22.13 | 2.2 | 47.2 | x | x | x | 5 |
| 176 | 111 | 123 | IVPNsAEERLHSM | S5(Phospho) | 2 | 781.856 | 33.02 | 2.8 | 35.6 | x | x | x | |
| 177 | 112 | 123 | VPNsAEERLHSM | S4(Phospho) | 2 | 725.311 | 27.58 | 2.8 | 60.0 | x | x | x | |
| 178 | 114 | 123 | NsAEERLHSM | S2(Phospho) | 2 | 627.252 | 23.47 | 1.8 | 46.0 | x | x | | 5 |
| 179 | 115 | 123 | SAEERLHSM | none | 2 | 530.247 | 17.95 | 1.9 | 58.6 | | | x | |
| 180 | 115 | 123 | sAEERLHSM | S1(Phospho) | 2 | 570.230 | 19.99 | 2.7 | 45.3 | | | x | |
| 181 | 125 | 144 | EGIHAQQKEPMIGVNQELAY | none | 3 | 752.377 | 39.50 | 2.9 | 46.1 | x | x | x | 5 |
| 182 | 125 | 150 | EGIHAQQKEPmIGVNQELAYFYPELF | M11(Ox) | 3 | 1023.168 | 63.21 | 2.7 | 22.5 | x | x | | |
| 183 | 131 | 144 | QKEPMIGVNQELAY | none | 2 | 810.411 | 40.43 | 2.5 | 72.3 | | | x | |
| 184 | 132 | 144 | KEPMIGVNQELAY | none | 2 | 746.380 | 40.35 | 2.9 | 65.4 | | | x | |
| 185 | 145 | 154 | FYPELFRQFY | none | 2 | 705.348 | 58.74 | 2.2 | 55.3 | x | x | x | 5 |
| 186 | 166 | 199 | YVPLGTQYTDAPSFSDIPNPIGSENSEKTTMPLW | none | 3 | 1252.600 | 65.31 | 3.4 | 54.3 | x | x | x | 5 |
| 187 | 166 | 199 | YVPLGTQYTDAPSFSDIPNPIGSENSEKTTmPLW | M31(Ox) | 3 | 1257.929 | 62.01 | 4.5 | 52.1 | x | x | x | 5 |
| 188 | 171 | 199 | TQYTDAPSFSDIPNPIGSENSEKTTmPLW | M26(Ox) | 3 | 1081.500 | 57.92 | 3.5 | 42.8 | | x | | |
| 189 | 172 | 194 | QYTDAPSFSDIPNPIGSENSEKT | none | 2 | 1249.073 | 47.31 | 3.5 | 61.6 | x | x | x | |
| 190 | 172 | 199 | QYTDAPSFSDIPNPIGSENSEKTTMPLW | none | 3 (2) | 1042.486 | 61.70 | 3.4 | 66.4 | x | x | x | |
| 191 | 172 | 199 | QYTDAPSFSDIPNPIGSENSEKTTmPLW | M25(Ox) | 3 (2) | 1047.818 | 57.99 | 4.0 | 59.5 | x | x | x | |
| 192 | 174 | 197 | TDAPSFSDIPNPIGSENSEKTTMP | none | 2 | 1268.082 | 49.71 | 3.0 | 50.8 | x | x | x | 5; 7 |
| 193 | 174 | 199 | TDAPSFSDIPNPIGSENSEKTTMPLW | none | 2 (3) | 1417.666 | 60.94 | 3.2 | 67.1 | x | x | x | 5 |
| 194 | 174 | 199 | TDAPSFSDIPNPIGSENSEKTTmPLW | M23(Ox) | 3 (2) | 950.777 | 57.11 | 3.6 | 69.4 | x | x | x | 5 |
| 195 | 176 | 190 | APSFSDIPNPIGSEN | none | 2 | 772.865 | 47.97 | 3.0 | 66.6 | | x | | |
| 196 | 176 | 193 | APSFSDIPNPIGSENSEK | none | 2 | 944.951 | 43.49 | 4.7 | 78.3 | x | x | x | |
| 197 | 176 | 199 | APSFSDIPNPIGSENSEKTTMPLW | none | 2 | 1309.627 | 59.81 | 4.6 | 94.1 | x | x | x | 1; 5; 6 |
| 198 | 176 | 199 | APSFSDIPNPIGSENSEKTTmPLW | M21(Ox) | 2 | 1317.626 | 56.21 | 4.2 | 86.6 | x | x | x | 3 |

| Nr. | Start pos. | End pos. | Sequence | Modification | z | *m/z* | $t_R$ (min) | Xcorr | -10lgP | RM[a] | UHT[a] | IF[a] | Literature (milk[1-6], colostrum[7]) |
|---|---|---|---|---|---|---|---|---|---|---|---|---|---|
| 199 | 177 | 199 | PSFSDIPNPIGSENSEKTTMPLW | none | 2 | 1274.110 | 59.48 | 3.5 | 74.3 | | x | | |
| 200 | 177 | 199 | PSFSDIPNPIGSENSEKTTmPLW | M20(Ox) | 2 | 1282.107 | 55.39 | 3.2 | 57.2 | | x | | |
| 201 | 178 | 199 | SFSDIPNPIGSENSEKTTMPLW | none | 2 | 1225.582 | 58.20 | 3.8 | 82.7 | x | x | x | |
| 202 | 178 | 199 | SFSDIPNPIGSENSEKTTmPLW | M19(Ox) | 2 (3) | 1233.580 | 53.81 | 3.7 | 76.0 | x | x | x | |
| 203 | 179 | 199 | FSDIPNPIGSENSEKTTMPLW | none | 2 | 1182.068 | 56.90 | 4.0 | 78.2 | x | x | x | 5 |
| 204 | 179 | 199 | FSDIPNPIGSENSEKTTmPLW | M18(Ox) | 2 | 1190.061 | 52.31 | 3.7 | 82.9 | | x | | |
| 205 | 180 | 193 | SDIPNPIGSENSEK | none | 2 | 743.853 | 29.93 | 2.2 | 53.4 | x | x | | 5 |
| 206 | 180 | 194 | SDIPNPIGSENSEKT | none | 2 | 794.379 | 30.65 | 3.7 | 73.9 | x | x | x | |
| 207 | 180 | 197 | SDIPNPIGSENSEKTTMP | none | 2 | 958.950 | 38.11 | 2.3 | 61.5 | x | x | x | 1; 5; 7 |
| 208 | 180 | 197 | SDIPNPIGSENSEKTTmP | M17(Ox) | 2 | 966.946 | 32.71 | 4.4 | 71.7 | x | x | x | 1; 5; 7 |
| 209 | 180 | 198 | SDIPNPIGSENSEKTTMPL | none | 2 | 1015.493 | 44.62 | 3.2 | 54.4 | x | x | x | |
| 210 | 180 | 199 | SDIPNPIGSENSEKTTMPLW | none | 2 (3) | 1108.531 | 53.03 | 4.2 | 99.0 | x | x | x | 1; 2; 5 |
| 211 | 180 | 199 | SDIPNPIGSENSEKTTmPLW | M17(Ox) | 2 (3) | 1116.529 | 48.45 | 4.3 | 98.7 | x | x | x | 1; 2; 5 |
| 212 | 181 | 197 | DIPNPIGSENSEKTTMP | none | 2 | 915.434 | 39.17 | 2.7 | 52.4 | x | x | x | 1; 5 |
| 213 | 181 | 197 | DIPNPIGSENSEKTTmP | M16(Ox) | 2 | 923.429 | 33.43 | 3.0 | 50.8 | x | x | x | 1; 5 |
| 214 | 181 | 199 | DIPNPIGSENSEKTTMPLW | none | 2 | 1065.015 | 54.84 | 3.9 | 78.7 | x | x | x | 1; 5 |
| 215 | 181 | 199 | DIPNPIGSENSEKTTmPLW | M16(Ox) | 2 | 1073.012 | 50.01 | 3.8 | 88.1 | x | x | x | |
| 216 | 182 | 193 | IPNPIGSENSEK | none | 2 | 642.826 | 25.99 | 2.7 | 52.1 | | x | | |
| 217 | 182 | 197 | IPNPIGSENSEKTTMP | none | 2 | 857.920 | 35.69 | 4.5 | 56.0 | x | x | x | 1; 2; 5 |
| 218 | 182 | 197 | IPNPIGSENSEKTTmP | M15(Ox) | 2 | 865.916 | 29.42 | 4.5 | 55.1 | x | x | x | 1 |
| 219 | 182 | 199 | IPNPIGSENSEKTTMPLW | none | 2 | 1007.501 | 51.48 | 4.4 | 100.8 | x | x | x | 1; 4; 5 |
| 220 | 182 | 199 | IPNPIGSENSEKTTmPLW | M15(Ox) | 2 | 1015.499 | 46.94 | 4.9 | 98.0 | x | x | x | 1 |
| 221 | 182 | 199 | IPNPIGsENSEKTTMPLW | S7(Phospho) | 2 | 1047.486 | 55.91 | 4.6 | 52.4 | | x | | |
| 222 | 183 | 199 | PNPIGSENSEKTTMPLW | none | 2 | 950.959 | 60.00 | 2.4 | 66.2 | x | x | x | |
| 223 | 183 | 199 | PNPIGSENSEKTTmPLW | M14(Ox) | 2 | 958.955 | 56.06 | 3.3 | 65.1 | x | x | x | |
| 224 | 184 | 197 | NPIGSENSEKTTMP | none | 2 | 752.851 | 25.82 | 2.2 | 46.8 | x | | x | 1; 7 |
| 225 | 184 | 197 | NPIGSENSEKTTmP | M13(Ox) | 2 | 760.848 | 19.12 | 2.3 | 58.0 | x | x | x | 5 |
| 226 | 184 | 199 | NPIGSENSEKTTMPLW | none | 2 | 902.435 | 45.67 | 3.6 | 87.8 | x | x | x | |
| 227 | 184 | 199 | NPIGSENSEKTTmPLW | M13(Ox) | 2 | 910.431 | 38.94 | 4.0 | 89.7 | x | x | x | |

| Nr. | Start pos. | End pos. | Sequence | Modification | z | *m/z* | $t_R$ (min) | Xcorr | -10lgP | RM[a] | UHT[a] | IF[a] | Literature (milk[1-6], colostrum[7]) |
|---|---|---|---|---|---|---|---|---|---|---|---|---|---|
| 228 | 185 | 197 | PIGSENSEKTTMP | none | 2 | 695.826 | 23.71 | 3.0 | 56.9 | x | x | x | 1; 3; 5 |
| 229 | 185 | 197 | PIGSENSEKTTmP | M12(Ox) | 2 | 703.826 | 16.47 | 3.3 | 62.3 | x | x | x | 3 |
| 230 | 185 | 198 | PIGSENSEKTTmPL | M12(Ox) | 2 | 760.369 | 24.90 | 2.4 | 52.5 |  | x |  |  |
| 231 | 185 | 199 | PIGSENSEKTTMPLW | none | 2 | 845.412 | 45.26 | 4.0 | 103.5 | x | x | x | 1; 4; 6 |
| 232 | 185 | 199 | PIGSENSEKTTmPLW | M12(Ox) | 2 | 853.410 | 38.98 | 3.7 | 96.8 | x | x | x |  |
| 233 | 185 | 199 | PIGsENSEKTTMPLW | S4(Phospho) | 2 | 885.395 | 49.91 | 3.2 | 64.1 |  | x |  |  |
| 234 | 186 | 197 | IGSENSEKTTMP | none | 2 | 647.302 | 21.38 | 1.8 | 52.1 | x | x | x |  |
| 235 | 186 | 199 | IGSENSEKTTMPLW | none | 2 | 796.886 | 43.86 | 2.9 | 73.8 | x | x | x |  |
| 236 | 186 | 199 | IGSENSEKTTmPLW | M11(Ox) | 2 | 804.884 | 36.41 | 2.8 | 63.7 | x | x | x |  |
| 237 | 188 | 199 | SENSEKTTMPLW | none | 2 | 711.833 | 41.59 | 3.2 | 66.1 | x | x | x |  |
| 238 | 188 | 199 | SENSEKTTmPLW | M9(Ox) | 2 | 719.829 | 33.19 | 2.8 | 67.6 | x | x | x |  |
| 239 | 189 | 199 | ENSEKTTMPLW | none | 2 | 668.316 | 41.90 | 2.2 | 58.9 |  | x | x |  |
| 240 | 189 | 199 | ENSEKTTmPLW | M8(Ox) | 2 | 676.313 | 33.41 | 2.7 | 49.0 |  | x | x |  |
| 241 | 190 | 199 | NSEKTTMPLW | none | 2 | 603.793 | 41.04 | 2.6 | 58.7 |  | x |  |  |
| 242 | 190 | 199 | NSEKTTmPLW | M7(Ox) | 2 | 611.791 | 32.42 | 2.3 | 56.9 |  | x |  |  |
| 243 | 191 | 199 | SEKTTMPLW | none | 2 | 546.773 | 41.20 | 2.1 | 47.5 | x | x | x | 1 |
| 244 | 191 | 199 | SEKTTmPLW | M6(Ox) | 2 | 554.770 | 32.54 | 2.5 | 49.9 | x | x | x |  |
| 245 | 192 | 199 | EKTTMPLW | none | 2 | 503.257 | 41.54 | 1.0 | 44.0 |  | x |  |  |
| 246 | 193 | 199 | KTTMPLW | none | 2 | 438.735 | 40.39 | 1.7 | 40.2 |  | x |  |  |
| **Alpha-S2-casein (P02663)** |  |  |  |  |  |  |  |  |  |  |  |  |  |
| 247 | 1 | 20 | KNTMEHVSSSEESIISQETY | none | 3 | 767.017 | 35.33 | 4.1 | 36.9 | x | x | x | 5 |
| 248 | 1 | 20 | KNTmEHVSSSEESIISQETY | M4(Ox) | 3 | 772.349 | 31.91 | 4.2 | 44.4 | x | x | x |  |
| 249 | 1 | 21 | KNTMEHVSSSEESIISQETYK | none | 3 (4) | 809.715 | 32.13 | 7.3 | 84.3 | x | x | x | 5 |
| 250 | 1 | 21 | KNTmEHVSSSEESIISQETYK | M4(Ox) | 3 (4) | 815.049 | 28.41 | 5.7 | 60.9 | x | x | x |  |
| 251 | 1 | 21 | KNTMEHVSSSEESIIsQETYK | S16(Phospho) | 3 | 836.371 | 33.11 | 7.4 | 86.0 | x | x | x | 5 |
| 252 | 1 | 23 | KNTMEHVSSSEESIISQETYKQE | none | 3 | 895.417 | 32.58 | 3.9 | 50.2 | x | x | x |  |
| 253 | 1 | 24 | KNTMEHVSSSEESIISQETYKQEK | none | 3 (4) | 938.119 | 30.37 | 7.1 | 69.4 | x | x | x | 1; 3; 5 |
| 254 | 1 | 24 | KNTmEHVSSSEESIISQETYKQEK | M4(Ox) | 4 (3) | 707.836 | 26.64 | 6.1 | 59.5 | x | x | x | 5 |
| 255 | 1 | 24 | KNTMEHVSSSEESIIsQETYKQEK | S16(Phospho) | 3 (4) | 964.769 | 31.28 | 6.3 | 63.9 | x | x | x | 3; 5 |

| Nr. | Start pos. | End pos. | Sequence | Modification | z | *m/z* | $t_R$ (min) | Xcorr | -10lgP | RM[a] | UHT[a] | IF[a] | Literature (milk[1-6], colostrum[7]) |
|---|---|---|---|---|---|---|---|---|---|---|---|---|---|
| 256 | 9 | 21 | SSEESIISQETYK | none | 2 | 750.858 | 30.66 | 3.1 | 58.6 | x | x | x | |
| 257 | 11 | 21 | EESIIsQETYK | S6(Phospho) | 2 | 703.808 | 32.97 | 2.7 | 51.5 | | | x | |
| 258 | 13 | 21 | SIIsQETYK | S4(Phospho) | 2 | 574.766 | 31.64 | 2.7 | 31.0 | | | x | |
| 259 | 14 | 21 | IIsQETYK | S3(Phospho) | 2 | 531.248 | 24.15 | 3.2 | 43.2 | x | x | x | 5 |
| 260 | 14 | 22 | IIsQETYKQ | S3(Phospho) | 2 | 595.275 | 24.14 | 2.7 | 46.8 | x | x | | 5 |
| 261 | 14 | 24 | IIsQETYKQEK | S3(Phospho) | 2 | 723.847 | 20.93 | 2.3 | 45.8 | x | x | x | |
| 262 | 71 | 78 | ITVDDKHY | none | 2 | 495.747 | 19.35 | 2.2 | 51.7 | x | x | x | |
| 263 | 71 | 79 | ITVDDKHYQ | none | 2 | 559.777 | 18.20 | 2.2 | 51.1 | x | x | x | 1 |
| 264 | 81 | 88 | ALNEINQF | none | 2 | 474.743 | 38.83 | 2.4 | 45.0 | | | x | |
| 265 | 81 | 89 | ALNEINQFY | none | 2 | 556.274 | 45.06 | 2.3 | 47.6 | | | x | |
| 266 | 81 | 95 | ALNEINQFYQKFPQY | none | 2 | 951.975 | 52.32 | 2.9 | 56.6 | | | x | |
| 267 | 81 | 98 | ALNEINQFYQKFPQYLQY | none | 3 (2) | 769.721 | 59.86 | 4.4 | 59.7 | | | x | 5 |
| 268 | 88 | 98 | FYQKFPQYLQY | none | 2 | 762.880 | 46.64 | 3.0 | 50.0 | | | x | |
| 269 | 89 | 95 | YQKFPQY | none | 2 | 487.242 | 28.61 | 2.2 | 52.2 | | | x | |
| 270 | 89 | 98 | YQKFPQYLQY | none | 2 | 689.348 | 42.20 | 3.1 | 64.3 | | | x | |
| 271 | 90 | 98 | QKFPQYLQY | none | 2 | 607.814 | 39.81 | 2.6 | 53.9 | | | x | |
| 272 | 98 | 113 | YLYQGPIVLNPWDQVK | none | 2 | 967.015 | 56.97 | 4.0 | 71.2 | x | x | | 5 |
| 273 | 99 | 113 | LYQGPIVLNPWDQVK | none | 3 (2) | 590.658 | 53.44 | 4.4 | 80.9 | x | x | x | 7 |
| 274 | 99 | 114 | LYQGPIVLNPWDQVKR | none | 2 (3) | 963.535 | 48.82 | 3.8 | 95.6 | x | x | x | |
| 275 | 99 | 115 | LYQGPIVLNPWDQVKRN | none | 2 (3) | 680.707 | 48.62 | 3.3 | 46.4 | | | x | |
| 276 | 100 | 112 | YQGPIVLNPWDQV | none | 2 | 764.895 | 58.72 | 3.0 | 41.4 | | | x | 7 |
| 277 | 100 | 113 | YQGPIVLNPWDQVK | none | 2 | 828.941 | 50.57 | 3.8 | 75.3 | x | x | x | 7 |
| 278 | 100 | 114 | YQGPIVLNPWDQVKR | none | 3 | 604.996 | 45.97 | 3.5 | 79.5 | x | x | x | |
| 279 | 100 | 115 | YQGPIVLNPWDQVKRN | none | 3 | 643.011 | 45.69 | 1.8 | 42.7 | | | x | |
| 280 | 101 | 113 | QGPIVLNPWDQVK | none | 2 | 747.409 | 47.66 | 2.8 | 63.9 | x | x | x | 5 |
| 281 | 101 | 114 | QGPIVLNPWDQVKR | none | 3 | 550.643 | 42.99 | 2.7 | 42.9 | x | x | x | |
| 282 | 102 | 112 | GPIVLNPWDQV | none | 2 | 619.333 | 55.93 | 2.1 | 36.4 | x | | | 5 |
| 283 | 102 | 113 | GPIVLNPWDQVK | none | 2 | 683.379 | 47.32 | 3.4 | 68.6 | x | x | x | 3; 5 |
| 284 | 105 | 113 | VLNPWDQVK | none | 2 | 549.799 | 37.70 | 2.2 | 51.1 | x | x | x | 1 |

| Nr. | Start pos. | End pos. | Sequence | Modification | z | *m/z* | $t_R$ (min) | Xcorr | -10lgP | RM[a] | UHT[a] | IF[a] | Literature (milk[1-6], colostrum[7]) |
|---|---|---|---|---|---|---|---|---|---|---|---|---|---|
| 285 | 115 | 128 | NAVPITPTLNREQL | none | 2 | 783.437 | 40.03 | 2.4 | 59.5 | | x | x | |
| 286 | 115 | 135 | NAVPITPTLNREQLSTSEENS | none | 2 | 1150.573 | 36.88 | 3.9 | 85.9 | x | x | x | 1 |
| 287 | 115 | 135 | NAVPITPTLNREQLSTsEENS | S17(Phospho) | 2 | 1190.556 | 38.62 | 4.1 | 58.5 | x | x | x | |
| 288 | 115 | 136 | NAVPITPTLNREQLSTsEENSK | S17(Phospho) | 2 | 1254.605 | 35.25 | 5.0 | 52.0 | x | x | x | |
| 289 | 115 | 149 | NAVPITPTLNREQLSTsEENSKKTVDMESTEVFTK | S17(Phospho) | 3 | 1335.314 | 42.34 | 4.1 | 32.3 | x | x | x | |
| 290 | 123 | 135 | LNREQLSTSEENS | none | 2 | 753.854 | 19.61 | 2.8 | 53.8 | x | x | x | |
| 291 | 123 | 136 | LNREQLSTsEENSK | S9(Phospho) | 2 (3) | 857.885 | 18.43 | 4.0 | 51.8 | x | x | x | |
| 292 | 123 | 137 | LNREQLSTsEENSKK | S9(Phospho) | 3 | 614.957 | 16.25 | 3.2 | 18.9 | x | x | x | |
| 293 | 123 | 149 | LNREQLSTsEENSKKTVDMESTEVFTK | S9(Phospho) | 4 | 803.377 | 34.35 | 4.9 | 56.4 | x | x | x | 5 |
| 294 | 135 | 149 | SKKTVDMEsTEVFTK | S9(Phospho) | 3 | 603.948 | 28.30 | 5.7 | 71.5 | x | x | x | |
| 295 | 137 | 149 | KTVDMESTEVFTK | none | 2 (3) | 757.873 | 30.56 | 4.4 | 85.5 | x | x | x | 1; 5 |
| 296 | 137 | 149 | KTVDmESTEVFTK | M5(Ox) | 2 | 510.916 | 23.93 | 2.4 | 42.9 | x | x | x | |
| 297 | 137 | 149 | KTVDMEsTEVFTK | S7(Phospho) | 2 (3) | 797.857 | 31.88 | 5.7 | 86.5 | x | x | x | 1; 3; 4; 5 |
| 298 | 137 | 150 | KTVDMESTEVFTKK | none | 3 | 548.281 | 27.36 | 5.7 | 74.0 | x | x | x | 3; 5 |
| 299 | 137 | 150 | KTVDMEsTEVFTKK | S7(Phospho) | 2 (3) | 861.905 | 28.46 | 5.1 | 65.3 | x | x | x | 1; 5 |
| 300 | 138 | 149 | TVDMESTEVFTK | none | 2 | 693.827 | 35.18 | 3.8 | 91.1 | x | x | x | 1; 3; 5 |
| 301 | 138 | 149 | TVDmESTEVFTK | M4(Ox) | 2 | 701.822 | 27.51 | 3.5 | 76.4 | x | x | x | 1; 3; 5 |
| 302 | 138 | 149 | TVDMEsTEVFTK | S6(Phospho) | 2 | 733.810 | 36.40 | 4.9 | 75.1 | x | x | x | 1; 3; 5 |
| 303 | 138 | 150 | TVDmESTEVFTKK | M4(Ox) | 2 (3) | 765.871 | 24.32 | 3.1 | 53.5 | x | x | | 3 |
| 304 | 139 | 149 | VDMESTEVFTK | none | 2 | 643.301 | 34.10 | 3.4 | 84.6 | x | x | x | 5 |
| 305 | 139 | 149 | VDMEsTEVFTK | S5(Phospho) | 2 | 683.286 | 36.36 | 4.6 | 70.1 | x | x | x | 5 |
| 306 | 139 | 150 | VDMESTEVFTKK | none | 3 | 471.901 | 30.15 | 2.1 | 43.3 | x | x | | |
| 307 | 139 | 150 | VDmESTEVFTKK | M3(Ox) | 3 | 477.232 | 22.39 | 3.1 | 28.7 | x | x | | |
| 308 | 140 | 149 | DmESTEVFTK | M2(Ox) | 2 | 601.764 | 24.01 | 2.7 | 61.1 | x | x | | |
| 309 | 140 | 149 | DMEsTEVFTK | S4(Phospho) | 2 | 633.751 | 33.55 | 3.4 | 56.9 | x | x | x | |
| 310 | 141 | 149 | MESTEVFTK | none | 2 | 536.254 | 25.53 | 2.2 | 51.7 | x | x | x | |
| 311 | 141 | 149 | MEsTEVFTK | S3(Phospho) | 2 | 576.238 | 28.02 | 3.7 | 56.6 | | | x | |
| 312 | 141 | 150 | MEsTEVFTKK | S3(Phospho) | 2 | 640.285 | 23.57 | 2.8 | 42.9 | x | x | x | |
| 313 | 150 | 163 | KTKLTEEEKNRLNF | none | 3 (4) | 583.987 | 25.16 | 3.7 | 48.0 | x | x | x | 1 |

| Nr. | Start pos. | End pos. | Sequence | Modification | z | *m/z* | $t_R$ (min) | Xcorr | -10lgP | RM[a] | UHT[a] | IF[a] | Literature (milk[1-6], colostrum[7]) |
|---|---|---|---|---|---|---|---|---|---|---|---|---|---|
| 314 | 150 | 165 | KTKLTEEEKNRLNFLK | none | 3 (2,4) | 664.379 | 28.24 | 3.6 | 48.2 | x | x | x | |
| 315 | 151 | 161 | TKLTEEEKNRL | none | 3 | 454.251 | 19.23 | 2.9 | 48.3 | x | x | x | |
| 316 | 151 | 162 | TKLTEEEKNRLN | none | 3 (2) | 492.265 | 17.03 | 3.2 | 50.5 | x | x | x | 1 |
| 317 | 151 | 163 | TKLTEEEKNRLNF | none | 3 | 541.289 | 29.00 | 3.5 | 67.3 | x | x | x | 1 |
| 318 | 151 | 164 | TKLTEEEKNRLNFL | none | 2 (3) | 867.971 | 36.78 | 4.1 | 90.9 | x | x | x | 1 |
| 319 | 151 | 165 | TKLTEEEKNRLNFLK | none | 2 (3,4) | 932.020 | 31.80 | 4.7 | 87.9 | x | x | x | 1; 5 |
| 320 | 152 | 163 | KLTEEEKNRLNF | none | 3 | 507.605 | 26.59 | 3.1 | 28.4 | x | x | x | 1; 5 |
| 321 | 152 | 164 | KLTEEEKNRLNFL | none | 3 (2) | 545.301 | 35.24 | 3.7 | 78.6 | x | x | x | 5 |
| 322 | 152 | 165 | KLTEEEKNRLNFLK | none | 4 (3) | 441.250 | 29.77 | 2.8 | 31.8 | x | x | | 5 |
| 323 | 153 | 161 | LTEEEKNRL | none | 2 | 566.302 | 16.94 | 1.9 | 39.3 | x | x | x | 1; 5 |
| 324 | 153 | 164 | LTEEEKNRLNFL | none | 2 (3) | 753.403 | 38.44 | 3.5 | 74.6 | x | x | x | 1 |
| 325 | 153 | 165 | LTEEEKNRLNFLK | none | 3 (2) | 545.301 | 32.01 | 3.4 | 44.1 | x | x | x | 1 |
| 326 | 154 | 163 | TEEEKNRLNF | none | 2 (3) | 640.317 | 24.36 | 3.0 | 56.3 | x | x | x | 1 |
| 327 | 154 | 164 | TEEEKNRLNFL | none | 2 (3) | 696.860 | 35.53 | 3.8 | 69.5 | x | x | x | 5 |
| 328 | 154 | 165 | TEEEKNRLNFLK | none | 3 | 507.604 | 27.91 | 3.1 | 40.3 | x | x | x | 5 |
| 329 | 155 | 164 | EEEKNRLNFL | none | 2 | 646.336 | 35.55 | 2.2 | 56.7 | x | x | x | 1 |
| 330 | 162 | 169 | NFLKKISQ | none | 2 | 489.291 | 24.74 | 2.4 | 44.6 | | | x | |
| 331 | 170 | 179 | RYQKFALPQY | none | 2 (3) | 657.354 | 35.84 | 2.1 | 55.8 | | | x | |
| 332 | 171 | 179 | YQKFALPQY | none | 2 | 579.304 | 40.61 | 2.2 | 56.2 | | | x | |
| 333 | 171 | 180 | YQKFALPQYL | none | 2 | 635.847 | 50.00 | 2.2 | 49.6 | x | x | x | 1; 5 |
| 334 | 172 | 179 | QKFALPQY | none | 2 | 497.771 | 37.54 | 1.5 | 43.3 | | | x | |
| 335 | 172 | 180 | QKFALPQYL | none | 2 | 554.313 | 47.59 | 1.8 | 38.7 | x | x | x | 5 |
| 336 | 174 | 181 | FALPQYLK | none | 2 | 490.284 | 44.25 | 2.3 | 38.6 | x | x | x | 1 |
| 337 | 174 | 187 | FALPQYLKTVYQHQ | none | 3 (2) | 579.310 | 48.81 | 3.7 | 66.7 | x | x | | 1 |
| 338 | 175 | 188 | ALPQYLKTVYQHQK | none | 3 | 572.985 | 34.66 | 2.8 | 40.6 | x | x | | 5 |
| 339 | 176 | 183 | LPQYLKTV | none | 2 | 481.288 | 35.91 | 2.7 | 43.1 | x | x | | 1 |
| 340 | 176 | 185 | LPQYLKTVYQ | none | 2 | 626.849 | 38.96 | 2.4 | 62.6 | x | x | x | |
| 341 | 189 | 196 | AMKPWIQP | none | 2 | 485.761 | 36.69 | 2.4 | 38.2 | x | x | x | 1 |
| 342 | 189 | 196 | AmKPWIQP | M2(Ox) | 2 | 493.759 | 30.92 | 2.0 | 30.9 | x | x | | 1 |

| Nr. | Start pos. | End pos. | Sequence | Modification | z | *m/z* | $t_R$ (min) | Xcorr | -10lgP | RM[a] | UHT[a] | IF[a] | Literature (milk[1-6], colostrum[7]) |
|---|---|---|---|---|---|---|---|---|---|---|---|---|---|
| 343 | 189 | 197 | AMKPWIQPK | none | 2 | 549.808 | 27.15 | 2.2 | 43.6 | | | x | 5 |
| 344 | 189 | 198 | AMKPWIQPKT | none | 3 (2) | 400.557 | 28.88 | 3.1 | 45.9 | x | x | x | 1 |
| 345 | 189 | 198 | AmKPWIQPKT | M2(Ox) | 3 (2) | 405.889 | 23.78 | 2.6 | 35.3 | x | x | | 1 |
| 346 | 189 | 203 | AMKPWIQPKTKVIPY | none | 4 (3) | 450.761 | 39.31 | 2.5 | 29.0 | | | x | |
| 347 | 191 | 198 | KPWIQPKT | none | 2 | 499.294 | 21.54 | 2.1 | 41.7 | x | x | | |
| 348 | 198 | 204 | TKVIPYV | none | 2 | 410.252 | 34.16 | 1.9 | 36.6 | x | x | x | 1 |
| 349 | 198 | 205 | TKVIPYVR | none | 2 | 488.301 | 24.73 | 2.5 | 37.9 | x | x | x | 1 |
| 350 | 198 | 206 | TKVIPYVRY | none | 2 | 569.833 | 32.02 | 2.4 | 46.1 | x | x | x | 1; 5 |
| 351 | 198 | 207 | TKVIPYVRYL | none | 3 (2) | 417.919 | 41.48 | 2.9 | 52.3 | x | x | x | 1; 3; 4; 5 |
| 352 | 199 | 205 | KVIPYVR | none | 2 | 437.778 | 22.91 | 2.2 | 21.9 | x | x | | |
| 353 | 199 | 206 | KVIPYVRY | none | 2 | 519.310 | 30.12 | 1.9 | 23.4 | x | x | | |
| 354 | 199 | 207 | KVIPYVRYL | none | 2 | 575.852 | 39.86 | 2.4 | 39.9 | x | x | x | 3; |
| 355 | 200 | 206 | VIPYVRY | none | 2 | 455.263 | 35.45 | 1.5 | 29.7 | x | x | x | |
| 356 | 200 | 207 | VIPYVRYL | none | 2 | 511.804 | 46.76 | 1.6 | 40.6 | x | x | x | 1; 3; 5 |
| 357 | 201 | 207 | IPYVRYL | none | 2 | 462.271 | 42.31 | 1.8 | 41.4 | x | x | x | 1; 4 |
| **Apolipoprotein A-I (P15497)** | | | | | | | | | | | | | |
| 358 | 235 | 247 | AAIDEASKKLNAQ | none | 2 | 679.868 | 22.31 | 3.6 | 74.7 | | | x | |
| **Beta-1,4-galactosyltransferase 1 (P08037)** | | | | | | | | | | | | | |
| 359 | 69 | 79 | AIGQPSGELRL | none | 2 | 570.822 | 37.19 | 2.2 | 37.5 | | | x | |
| 360 | 54 | 69 | VGVHPPLQGSSHGAAA | none | 3 | 495.590 | 22.64 | 2.8 | 31.5 | x | x | | |
| **Beta-2-microglobulin (P01888)** | | | | | | | | | | | | | |
| 361 | 1 | 10 | IQRPPKIQVY | none | 2 | 621.371 | 29.68 | 2.1 | 49.7 | | | x | |
| **Beta-casein (P02666)** | | | | | | | | | | | | | |
| 362 | 1 | 7 | RELEELN | none | 2 | 451.731 | 20.15 | 1.7 | 30.3 | | x | | |
| 363 | 1 | 10 | RELEELNVPG | none | 2 | 578.302 | 33.29 | 1.7 | 43.5 | x | x | x | |
| 364 | 1 | 11 | RELEELNVPGE | none | 2 | 642.824 | 34.48 | 2.5 | 54.6 | x | x | x | 1; 4; 6 |
| 365 | 1 | 14 | RELEELNVPGEIVE | none | 2 | 813.425 | 43.62 | 2.4 | 69.3 | | | x | 5 |
| 366 | 1 | 15 | RELEELNVPGEIVES | none | 2 | 856.939 | 43.36 | 2.6 | 70.3 | x | x | x | 1; 5 |
| 367 | 1 | 16 | RELEELNVPGEIVESL | none | 2 | 913.483 | 58.41 | 2.5 | 55.3 | x | x | x | |

| Nr. | Start pos. | End pos. | Sequence | Modification | z | *m/z* | $t_R$ (min) | Xcorr | -10lgP | RM[a] | UHT[a] | IF[a] | Literature (milk[1-6], colostrum[7]) |
|---|---|---|---|---|---|---|---|---|---|---|---|---|---|
| 368 | 1 | 16 | RELEELNVPGEIVEsL | S15(Phospho) | 2 | 953.466 | 54.67 | 3.5 | 57.2 | | x | x | 3 |
| 369 | 1 | 22 | RELEELNVPGEIVESLSSSEES | none | 2 | 1216.588 | 58.86 | 4.3 | 82.0 | x | x | x | 1; 5 |
| 370 | 1 | 25 | RELEELNVPGEIVESLSSSEESITR | none | 3 | 934.805 | 61.58 | 5.3 | 76.1 | x | x | x | 1; 2; 3; 5 |
| 371 | 1 | 27 | RELEELNVPGEIVESLSSSEESITRIN | none | 3 | 1010.515 | 64.91 | 4.4 | 49.8 | x | x | x | 1; 3; 5 |
| 372 | 1 | 27 | RELEELNVPGEIVESLSSsEESITRIN | S19(Phospho) | 3 | 1037.170 | 59.61 | 4.7 | 66.5 | x | x | x | 5 |
| 373 | 1 | 27 | RELEELNVPGEIVESLSsSEESITRIN | S18(Phospho) | 3 | 1037.173 | 62.51 | 4.7 | 71.9 | x | x | x | 5 |
| 374 | 1 | 28 | RELEELNVPGEIVESLSSSEESITRINK | none | 3 (4) | 1053.213 | 62.25 | 7.5 | 108.7 | x | x | x | 5 |
| 375 | 1 | 28 | RELEELNVPGEIVESLSSsEESITRINK | S19(Phospho) | 4 | 810.153 | 56.41 | 3.0 | 61.1 | x | x | x | 5 |
| 376 | 1 | 28 | RELEELNVPGEIVESLsSSEESITRINK | S17(Phospho) | 4 | 810.156 | 56.89 | 6.0 | 83.9 | x | x | x | 5 |
| 377 | 1 | 28 | RELEELNVPGEIVESLSsSEESITRINK | S18(Phospho) | 3 (4) | 1079.871 | 60.17 | 6.3 | 23.6 | x | x | x | 5 |
| 378 | 2 | 11 | ELEELNVPGE | none | 2 | 564.775 | 39.27 | 2.1 | 44.9 | x | x | x | 5; 7 |
| 379 | 2 | 15 | ELEELNVPGEIVES | none | 2 | 778.888 | 48.81 | 2.5 | 52.2 | x | x | x | 5 |
| 380 | 7 | 16 | NVPGEIVESL | none | 2 | 528.782 | 47.86 | 2.1 | 56.8 | | | x | |
| 381 | 16 | 25 | LSSsEESITR | S4(Phospho) | 2 | 594.760 | 21.90 | 2.3 | 29.1 | x | x | x | |
| 382 | 17 | 27 | SSSEESITRIN | none | 2 | 611.797 | 22.75 | 1.8 | 45.8 | | | x | |
| 383 | 18 | 27 | SSEESITRIN | none | 2 | 568.283 | 22.23 | 2.6 | 48.4 | | | x | |
| 384 | 26 | 48 | INKKIEKFQSEEQQQTEDELQDK | none | 4 | 709.605 | 29.21 | 4.1 | 24.2 | x | x | x | |
| 385 | 26 | 48 | INKKIEKFQsEEQQQTEDELQDK | S10(Phospho) | 3 (4) | 972.461 | 30.94 | 5.4 | 54.7 | x | x | x | |
| 386 | 29 | 39 | KIEKFQsEEQQ | S7(Phospho) | 2 | 737.334 | 17.73 | 3.1 | 45.3 | x | x | x | |
| 387 | 29 | 40 | KIEKFQsEEQQQ | S7(Phospho) | 2 | 801.364 | 18.30 | 3.7 | 47.9 | x | x | x | |
| 388 | 29 | 42 | KIEKFQsEEQQQTE | S7(Phospho) | 2 | 916.410 | 20.81 | 3.3 | 43.6 | x | x | x | |
| 389 | 29 | 43 | KIEKFQsEEQQQTED | S7(Phospho) | 2 (3) | 973.922 | 21.13 | 3.9 | 53.5 | x | x | x | |
| 390 | 29 | 46 | KIEKFQsEEQQQTEDELQ | S7(Phospho) | 3 (2) | 773.011 | 30.02 | 4.6 | 38.4 | x | x | x | |
| 391 | 29 | 47 | KIEKFQSEEQQQTEDELQD | none | 3 (2) | 784.699 | 27.70 | 5.0 | 67.4 | x | x | x | |
| 392 | 29 | 47 | KIEKFQsEEQQQTEDELQD | S7(Phospho) | 3 (2) | 811.356 | 30.43 | 5.8 | 67.3 | x | x | x | |
| 393 | 29 | 48 | KIEKFQSEEQQQTEDELQDK | none | 3 (2,4) | 827.398 | 26.72 | 7.9 | 98.6 | x | x | x | 1; 3; 5 |
| 394 | 29 | 48 | KIEKFQsEEQQQTEDELQDK | S7(Phospho) | 3 (2,4) | 854.054 | 29.03 | 7.9 | 85.7 | x | x | x | 1; 3; 5 |
| 395 | 29 | 52 | KIEKFQSEEQQQTEDELQDKIHPF | none | 4 | 744.365 | 40.63 | 5.4 | 57.0 | x | x | x | 5 |
| 396 | 29 | 52 | KIEKFQsEEQQQTEDELQDKIHPF | S7(Phospho) | 4 (3) | 764.357 | 42.50 | 5.8 | 46.8 | x | x | x | |

| Nr. | Start pos. | End pos. | Sequence | Modification | z | *m/z* | $t_R$ (min) | Xcorr | -10lgP | RM[a] | UHT[a] | IF[a] | Literature (milk[1-6], colostrum[7]) |
|---|---|---|---|---|---|---|---|---|---|---|---|---|---|
| 397 | 29 | 53 | KIEKFQSEEQQQTEDELQDKIHPFA | none | 4 | 762.125 | 40.51 | 6.2 | 65.4 | x | x | x | 2 |
| 398 | 29 | 53 | KIEKFQsEEQQQTEDELQDKIHPFA | S7(Phospho) | 4 (3) | 782.117 | 42.22 | 5.4 | 54.7 | x | x | x | |
| 399 | 29 | 55 | KIEKFQsEEQQQTEDELQDKIHPFAQT | S7(Phospho) | 4 (3) | 839.395 | 41.94 | 6.8 | 40.4 | x | x | x | |
| 400 | 29 | 56 | KIEKFQSEEQQQTEDELQDKIHPFAQTQ | none | 4 | 851.417 | 39.67 | 6.0 | 43.3 | x | x | x | |
| 401 | 29 | 56 | KIEKFQsEEQQQTEDELQDKIHPFAQTQ | S7(Phospho) | 4 (3) | 871.409 | 41.33 | 6.6 | 43.8 | x | x | x | |
| 402 | 29 | 57 | KIEKFQSEEQQQTEDELQDKIHPFAQTQS | none | 3 (4,5) | 1163.900 | 39.49 | 6.0 | 78.7 | x | x | x | |
| 403 | 29 | 57 | KIEKFQsEEQQQTEDELQDKIHPFAQTQS | S7(Phospho) | 4 (3) | 893.167 | 41.22 | 6.0 | 48.4 | x | x | x | |
| 404 | 30 | 39 | IEKFQsEEQQ | S6(Phospho) | 2 | 673.287 | 20.70 | 3.1 | 39.2 | x | x | x | |
| 405 | 30 | 40 | IEKFQsEEQQQ | S6(Phospho) | 2 | 737.317 | 21.03 | 3.7 | 53.8 | | | x | |
| 406 | 30 | 43 | IEKFQsEEQQQTED | S6(Phospho) | 2 | 909.874 | 23.92 | 3.4 | 45.0 | | x | x | 7 |
| 407 | 30 | 47 | IEKFQSEEQQQTEDELQD | none | 2 | 1112.498 | 30.61 | 4.8 | 64.7 | x | x | x | |
| 408 | 30 | 47 | IEKFQsEEQQQTEDELQD | S6(Phospho) | 2 | 1152.483 | 33.60 | 6.4 | 74.1 | x | x | x | |
| 409 | 30 | 48 | IEKFQSEEQQQTEDELQDK | none | 3 (2) | 784.701 | 28.96 | 7.4 | 82.0 | x | x | x | 3; 5 |
| 410 | 30 | 48 | IEKFQsEEQQQTEDELQDK | S6(Phospho) | 3 (2) | 811.356 | 31.54 | 7.0 | 71.9 | x | x | x | 3; 5 |
| 411 | 30 | 53 | IEKFQsEEQQQTEDELQDKIHPFA | S6(Phospho) | 3 | 999.791 | 44.74 | 5.9 | 56.4 | x | x | x | |
| 412 | 30 | 56 | IEKFQsEEQQQTEDELQDKIHPFAQTQ | S6(Phospho) | 3 | 1118.845 | 43.65 | 6.1 | 56.4 | x | x | x | |
| 413 | 30 | 57 | IEKFQsEEQQQTEDELQDKIHPFAQTQS | S6(Phospho) | 3 | 1147.857 | 43.52 | 6.2 | 43.8 | x | x | x | |
| 414 | 32 | 48 | KFQSEEQQQTEDELQDK | none | 3 (2) | 703.989 | 24.49 | 5.6 | 61.2 | x | x | x | 1; 3; 5 |
| 415 | 32 | 48 | KFQsEEQQQTEDELQDK | S4(Phospho) | 2 (3) | 1095.465 | 28.30 | 6.8 | 77.9 | x | x | x | 3; 5 |
| 416 | 33 | 48 | FQSEEQQQTEDELQDK | none | 2 (3) | 991.432 | 26.81 | 5.6 | 100.3 | x | x | x | 3; 5 |
| 417 | 33 | 48 | FQsEEQQQTEDELQDK | S3(Phospho) | 2 (3) | 1031.416 | 30.17 | 7.1 | 92.6 | x | x | x | 3; 5 |
| 418 | 33 | 51 | FQSEEQQQTEDELQDKIHP | none | 3 | 777.024 | 35.99 | 3.2 | 35.8 | x | x | x | |
| 419 | 33 | 53 | FQsEEQQQTEDELQDKIHPFA | S3(Phospho) | 3 | 876.382 | 45.96 | 4.7 | 59.7 | x | x | x | |
| 420 | 33 | 56 | FQSEEQQQTEDELQDKIHPFAQTQ | none | 3 | 968.781 | 41.90 | 3.8 | 55.2 | x | x | | |
| 421 | 33 | 57 | FQSEEQQQTEDELQDKIHPFAQTQS | none | 3 | 997.794 | 41.74 | 4.6 | 57.4 | x | x | x | |
| 422 | 33 | 57 | FQsEEQQQTEDELQDKIHPFAQTQS | S3(Phospho) | 3 | 1024.448 | 44.37 | 4.2 | 56.3 | x | x | x | |
| 423 | 35 | 48 | SEEQQQTEDELQDK | none | 2 | 853.870 | 20.82 | 4.8 | 98.9 | x | x | x | 3; 5 |
| 424 | 35 | 48 | sEEQQQTEDELQDK | S1(Phospho) | 2 | 893.854 | 23.02 | 5.3 | 66.0 | x | x | x | 5 |
| 425 | 36 | 48 | EEQQQTEDELQDK | none | 2 | 810.352 | 18.76 | 3.2 | 72.5 | x | x | x | |

| Nr. | Start pos. | End pos. | Sequence | Modification | z | *m/z* | $t_R$ (min) | Xcorr | -10lgP | RM[a] | UHT[a] | IF[a] | Literature (milk[1-6], colostrum[7]) |
|---|---|---|---|---|---|---|---|---|---|---|---|---|---|
| 426 | 37 | 48 | EQQQTEDELQDK | none | 2 | 745.834 | 16.48 | 2.1 | 37.3 | | | x | |
| 427 | 41 | 49 | TEDELQDKI | none | 2 | 545.765 | 28.47 | 3.2 | 52.6 | x | x | | 3; 5 |
| 428 | 43 | 51 | DELQDKIHP | none | 2 | 547.777 | 25.81 | 2.2 | 37.7 | x | x | | |
| 429 | 49 | 56 | IHPFAQTQ | none | 2 | 471.244 | 21.27 | 2.0 | 39.8 | | | x | 1 |
| 430 | 49 | 57 | IHPFAQTQS | none | 2 | 514.761 | 21.63 | 2.0 | 52.0 | | | x | |
| 431 | 57 | 68 | SLVYPFPGPIPN | none | 2 | 650.852 | 55.36 | 2.1 | 54.5 | | | x | 5; 7 |
| 432 | 57 | 72 | SLVYPFPGPIPNSLPQ | none | 2 | 863.465 | 59.58 | 2.2 | 62.8 | | | x | |
| 433 | 58 | 72 | LVYPFPGPIPNSLPQ | none | 2 | 819.948 | 58.52 | 2.4 | 62.8 | | | x | |
| 434 | 69 | 91 | SLPQNIPPLTQTPVVVPPFLQPE | none | 2 (3) | 1256.203 | 67.12 | 2.7 | 60.7 | | | x | |
| 435 | 69 | 105 | SLPQNIPPLTQTPVVVPPFLQPEVMGVSKVKEAMAPK | none | 4 | 992.555 | 65.99 | 2.0 | 22.5 | x | x | x | |
| 436 | 73 | 91 | NIPPLTQTPVVVPPFLQPE | none | 3 (2) | 696.058 | 65.52 | 3.1 | 58.1 | | | x | |
| 437 | 83 | 95 | VVPPFLQPEVmGV | M11(Ox) | 2 | 714.384 | 55.39 | 1.6 | 33.8 | x | x | | 5 |
| 438 | 83 | 98 | VVPPFLQPEVMGVSKV | none | 2 | 863.484 | 56.12 | 1.9 | 57.3 | x | x | x | 5 |
| 439 | 84 | 95 | VPPFLQPEVMGV | none | 2 | 656.852 | 59.21 | 1.9 | 43.6 | x | x | x | 1; 3; 5; 7 |
| 440 | 84 | 95 | VPPFLQPEVmGV | M10(Ox) | 2 | 664.849 | 51.70 | 2.2 | 48.6 | x | x | x | 1; 5 |
| 441 | 84 | 98 | VPPFLQPEVMGVSKV | none | 2 | 813.949 | 52.95 | 3.3 | 83.5 | x | x | x | 5 |
| 442 | 106 | 123 | HKEMPFPKYPVEPFTESQ | none | 3 | 731.024 | 41.93 | 3.6 | 50.7 | x | x | x | 2; 5 |
| 443 | 106 | 124 | HKEMPFPKYPVEPFTESQS | none | 3 | 760.033 | 41.43 | 3.7 | 59.6 | x | x | x | 1; 5 |
| 444 | 106 | 124 | HKEmPFPKYPVEPFTESQS | M4(Ox) | 3 | 765.364 | 37.95 | 3.6 | 42.2 | x | x | x | 5 |
| 445 | 106 | 125 | HKEMPFPKYPVEPFTESQSL | none | 3 (4,2) | 797.727 | 46.12 | 4.2 | 77.8 | x | x | x | 1; 2; 3; 5 |
| 446 | 106 | 125 | HKEmPFPKYPVEPFTESQSL | M4(Ox) | 3 | 803.061 | 43.36 | 3.4 | 46.5 | x | x | x | 5 |
| 447 | 106 | 132 | HKEMPFPKYPVEPFTESQSLTLTDVEN | none | 3 | 1055.181 | 50.91 | 3.9 | 49.4 | x | x | x | |
| 448 | 106 | 133 | HKEMPFPKYPVEPFTESQSLTLTDVENL | none | 3 (4) | 1092.878 | 57.53 | 4.3 | 37.4 | x | x | x | 5 |
| 449 | 107 | 124 | KEMPFPKYPVEPFTESQS | none | 3 | 714.347 | 44.61 | 3.4 | 39.8 | x | x | | |
| 450 | 107 | 125 | KEMPFPKYPVEPFTESQSL | none | 3 (2) | 752.040 | 49.39 | 4.1 | 66.8 | x | x | x | 5 |
| 451 | 107 | 125 | KEmPFPKYPVEPFTESQSL | M3(Ox) | 3 | 757.373 | 46.60 | 3.7 | 38.3 | x | x | | |
| 452 | 108 | 119 | EMPFPKYPVEPF | none | 2 | 740.864 | 53.51 | 2.9 | 44.8 | | | x | 7 |
| 453 | 108 | 122 | EMPFPKYPVEPFTES | none | 2 | 899.424 | 51.40 | 2.4 | 48.9 | x | x | x | 7 |
| 454 | 108 | 123 | EMPFPKYPVEPFTESQ | none | 2 | 963.454 | 50.75 | 3.8 | 70.5 | x | x | x | 7 |

| Nr. | Start pos. | End pos. | Sequence | Modification | z | *m/z* | $t_R$ (min) | Xcorr | -10lgP | RM[a] | UHT[a] | IF[a] | Literature (milk[1-6], colostrum[7]) |
|---|---|---|---|---|---|---|---|---|---|---|---|---|---|
| 455 | 108 | 124 | EMPFPKYPVEPFTESQS | none | 2 (3) | 1006.970 | 50.30 | 4.2 | 89.8 | x | x | x | 1; 5; 7 |
| 456 | 108 | 124 | EmPFPKYPVEPFTESQS | M2(Ox) | 2 | 1014.968 | 45.27 | 3.9 | 78.9 | x | x | x | 7; 5 |
| 457 | 108 | 125 | EmPFPKYPVEPFTESQSL | M2(Ox) | 2 | 1071.509 | 50.77 | 4.8 | 84.8 | x | x | x | 1; 5 |
| 458 | 108 | 126 | EMPFPKYPVEPFTESQSLT | none | 2 | 1114.036 | 53.94 | 3.4 | 56.0 | x | x | | |
| 459 | 109 | 119 | MPFPKYPVEPF | none | 2 | 676.342 | 52.07 | 3.1 | 51.9 | x | | x | 7; 5 |
| 460 | 109 | 120 | MPFPKYPVEPFT | none | 2 | 726.864 | 50.70 | 3.4 | 63.0 | x | x | x | 7 |
| 461 | 109 | 123 | MPFPKYPVEPFTESQ | none | 2 | 898.932 | 49.28 | 4.3 | 77.2 | x | x | x | 7 |
| 462 | 109 | 124 | MPFPKYPVEPFTESQS | none | 2 | 942.447 | 48.83 | 4.3 | 92.9 | x | x | x | 1; 5 |
| 463 | 109 | 124 | mPFPKYPVEPFTESQS | M1(Ox) | 2 | 950.446 | 44.33 | 3.7 | 79.9 | x | x | | |
| 464 | 109 | 125 | MPFPKYPVEPFTESQSL | none | 2 | 998.991 | 53.76 | 4.8 | 81.9 | x | x | x | 1; 5 |
| 465 | 109 | 125 | mPFPKYPVEPFTESQSL | M1(Ox) | 2 (3) | 1006.988 | 49.86 | 4.4 | 76.1 | x | x | x | |
| 466 | 109 | 126 | MPFPKYPVEPFTESQSLT | none | 2 | 1049.514 | 52.68 | 3.8 | 53.4 | x | x | | |
| 467 | 111 | 124 | FPKYPVEPFTESQS | none | 2 | 828.402 | 42.85 | 3.5 | 65.3 | x | x | x | |
| 468 | 111 | 125 | FPKYPVEPFTESQSL | none | 2 | 884.943 | 48.88 | 4.5 | 97.3 | x | x | x | 1; 5; 7 |
| 469 | 114 | 125 | YPVEPFTESQSL | none | 2 | 698.835 | 46.59 | 2.3 | 49.7 | x | x | x | 1; 5; 7 |
| 470 | 115 | 123 | PVEPFTESQ | none | 2 | 517.245 | 30.39 | 1.9 | 48.8 | | | x | 7 |
| 471 | 115 | 124 | PVEPFTESQS | none | 2 | 560.762 | 30.03 | 2.3 | 45.6 | | | x | 7 |
| 472 | 115 | 125 | PVEPFTESQSL | none | 2 | 617.305 | 41.63 | 2.1 | 56.8 | | | x | 7 |
| 473 | 124 | 133 | SLTLTDVENL | none | 2 | 552.793 | 51.64 | 2.4 | 62.9 | | | x | 5 |
| 474 | 126 | 143 | TLTDVENLHLPLPLLQSW | none | 2 (3) | 1045.073 | 73.46 | 3.2 | 58.7 | | | x | |
| 475 | 128 | 143 | TDVENLHLPLPLLQSW | none | 2 | 938.005 | 70.48 | 3.6 | 57.9 | | | x | |
| 476 | 129 | 139 | DVENLHLPLPL | none | 2 | 630.354 | 59.63 | 2.3 | 34.8 | | | x | |
| 477 | 129 | 143 | DVENLHLPLPLLQSW | none | 2 | 887.482 | 72.06 | 3.2 | 57.8 | | | x | |
| 478 | 132 | 143 | NLHLPLPLLQSW | none | 2 | 715.912 | 68.77 | 2.5 | 52.1 | | | x | |
| 479 | 134 | 143 | HLPLPLLQSW | none | 2 | 602.349 | 61.21 | 2.6 | 46.2 | | | x | |
| 480 | 144 | 154 | MHQPHQPLPPT | none | 2 | 641.821 | 20.36 | 2.5 | 52.3 | | | x | |
| 481 | 144 | 162 | MHQPHQPLPPTVMFPPQSV | none | 3 | 723.365 | 45.71 | 3.4 | 45.7 | | | x | 7 |
| 482 | 144 | 162 | mHQPHQPLPPTVMFPPQSV | M1(Ox) | 3 | 728.696 | 44.15 | 2.6 | 28.9 | | | x | |
| 483 | 144 | 163 | MHQPHQPLPPTVMFPPQSVL | none | 3 | 761.061 | 51.23 | 2.8 | 46.5 | | | x | 7 |

| Nr. | Start pos. | End pos. | Sequence | Modification | z | *m/z* | $t_R$ (min) | Xcorr | -10lgP | RM[a] | UHT[a] | IF[a] | Literature (milk[1-6], colostrum[7]) |
|---|---|---|---|---|---|---|---|---|---|---|---|---|---|
| 484 | 144 | 165 | MHQPHQPLPPTVMFPPQSVLSL | none | 3 | 827.764 | 57.91 | 3.5 | 45.2 | | | x | |
| 485 | 144 | 165 | mHQPHQPLPPTVMFPPQSVLSL | M1(Ox) | 3 | 833.097 | 57.74 | 2.7 | 20.5 | | | x | |
| 486 | 144 | 168 | MHQPHQPLPPTVMFPPQSVLSLSQS | none | 3 | 928.475 | 54.60 | 2.9 | 38.3 | | | x | |
| 487 | 161 | 169 | SVLSLSQSK | none | 2 | 474.770 | 26.09 | 2.3 | 53.0 | | | x | |
| 488 | 163 | 175 | LSLSQSKVLPVPQ | none | 2 | 698.414 | 40.81 | 3.0 | 51.2 | | | x | |
| 489 | 163 | 182 | LSLSQSKVLPVPQKAVPYPQ | none | 3 | 727.089 | 42.38 | 3.7 | 48.2 | | | x | |
| 490 | 163 | 190 | LSLSQSKVLPVPQKAVPYPQRDMPIQAF | none | 3 (4) | 1046.580 | 50.43 | 5.0 | 73.9 | x | x | x | |
| 491 | 164 | 175 | SLSQSKVLPVPQ | none | 2 | 641.872 | 33.16 | 2.6 | 63.0 | x | | x | |
| 492 | 164 | 176 | SLSQSKVLPVPQK | none | 2 (3) | 705.920 | 27.20 | 3.8 | 71.4 | x | x | x | 1 |
| 493 | 164 | 182 | SLSQSKVLPVPQKAVPYPQ | none | 3 (2) | 689.394 | 37.37 | 3.7 | 79.8 | | | x | |
| 494 | 164 | 190 | SLSQSKVLPVPQKAVPYPQRDMPIQAF | none | 3 (4) | 1008.884 | 47.49 | 5.0 | 70.9 | x | x | x | 5 |
| 495 | 165 | 175 | LSQSKVLPVPQ | none | 2 | 598.358 | 31.14 | 2.5 | 35.7 | x | x | x | |
| 496 | 166 | 175 | SQSKVLPVPQ | none | 2 | 541.812 | 26.71 | 2.5 | 56.1 | | | x | |
| 497 | 166 | 176 | SQSKVLPVPQK | none | 2 (3) | 605.860 | 20.63 | 3.3 | 68.0 | x | x | x | |
| 498 | 166 | 182 | SQSKVLPVPQKAVPYPQ | none | 2 (3) | 933.533 | 33.87 | 2.6 | 65.6 | | | x | |
| 499 | 166 | 190 | SQSKVLPVPQKAVPYPQRDMPIQAF | none | 3 | 942.179 | 45.43 | 4.6 | 58.7 | x | | x | |
| 500 | 167 | 175 | QSKVLPVPQ | none | 2 | 498.297 | 26.45 | 1.7 | 23.7 | | | x | |
| 501 | 168 | 175 | SKVLPVPQ | none | 2 | 434.267 | 26.11 | 2.3 | 30.6 | | | x | |
| 502 | 168 | 182 | SKVLPVPQKAVPYPQ | none | 3 | 550.991 | 33.37 | 1.9 | 56.4 | | | x | |
| 503 | 170 | 182 | VLPVPQKAVPYPQ | none | 2 | 718.420 | 36.52 | 2.2 | 74.9 | | | x | |
| 504 | 170 | 189 | VLPVPQKAVPYPQRDMPIQA | none | 3 | 749.748 | 41.93 | 3.2 | 54.9 | x | x | x | 5 |
| 505 | 170 | 190 | VLPVPQKAVPYPQRDMPIQAF | none | 3 | 798.771 | 48.74 | 3.6 | 66.4 | x | x | x | 5 |
| 506 | 170 | 190 | VLPVPQKAVPYPQRDmPIQAF | M16(Ox) | 3 | 804.105 | 44.20 | 3.3 | 67.4 | x | x | x | |
| 507 | 171 | 179 | LPVPQKAVP | none | 2 | 474.797 | 28.99 | 1.9 | 39.6 | | x | | |
| 508 | 171 | 182 | LPVPQKAVPYPQ | none | 2 | 668.884 | 33.86 | 1.9 | 57.1 | | | x | |
| 509 | 171 | 186 | LPVPQKAVPYPQRDMP | none | 3 | 612.665 | 36.41 | 3.3 | 61.9 | x | x | x | 5 |
| 510 | 171 | 189 | LPVPQKAVPYPQRDMPIQA | none | 3 | 716.725 | 40.01 | 3.4 | 60.3 | x | x | x | |
| 511 | 171 | 190 | LPVPQKAVPYPQRDMPIQAF | none | 3 | 765.748 | 47.14 | 2.5 | 24.2 | x | x | x | 5 |
| 512 | 176 | 182 | KAVPYPQ | none | 2 | 401.726 | 17.58 | 2.3 | 32.7 | | | x | |

| Nr. | Start pos. | End pos. | Sequence | Modification | z | *m/z* | $t_R$ (min) | Xcorr | -10lgP | RM[a] | UHT[a] | IF[a] | Literature (milk[1-6], colostrum[7]) |
|---|---|---|---|---|---|---|---|---|---|---|---|---|---|
| 513 | 176 | 190 | KAVPYPQRDMPIQAF | none | 2 (3) | 880.961 | 40.78 | 3.3 | 78.0 | | | x | |
| 514 | 177 | 188 | AVPYPQRDMPIQ | none | 2 | 707.863 | 34.03 | 2.3 | 53.9 | x | x | x | 7 |
| 515 | 177 | 189 | AVPYPQRDMPIQA | none | 2 | 743.380 | 36.24 | 2.7 | 60.2 | x | x | x | |
| 516 | 177 | 189 | AVPYPQRDmPIQA | M9(Ox) | 2 | 751.378 | 29.73 | 2.2 | 50.8 | x | x | x | |
| 517 | 177 | 190 | AVPYPQRDMPIQAF | none | 2 | 816.914 | 46.08 | 2.3 | 69.2 | | | x | 5; 7 |
| 518 | 177 | 190 | AVPYPQRDmPIQAF | M9(Ox) | 2 | 824.913 | 39.44 | 2.7 | 65.3 | | | x | |
| 519 | 177 | 191 | AVPYPQRDMPIQAFL | none | 2 | 873.458 | 56.17 | 2.0 | 51.9 | | | x | |
| 520 | 177 | 209 | AVPYPQRDMPIQAFLLYQEPVLGPVRGPFPIIV | none | 3 | 1241.015 | 76.08 | 4.9 | 56.7 | x | x | x | 5 |
| 521 | 177 | 209 | AVPYPQRDmPIQAFLLYQEPVLGPVRGPFPIIV | M9(Ox) | 3 | 1246.351 | 73.24 | 4.2 | 43.1 | x | x | x | |
| 522 | 178 | 184 | VPYPQRD | none | 2 | 437.723 | 16.09 | 1.4 | 32.5 | x | x | | |
| 523 | 178 | 186 | VPYPQRDmP | M8(Ox) | 2 | 559.768 | 21.20 | 2.3 | 42.8 | x | x | | 7 |
| 524 | 178 | 188 | VPYPQRDMPIQ | none | 2 | 672.341 | 32.40 | 3.4 | 60.2 | x | x | x | 1; 7 |
| 525 | 178 | 188 | VPYPQRDmPIQ | M8(Ox) | 2 | 680.338 | 25.25 | 2.9 | 47.6 | x | x | | 1; 7 |
| 526 | 178 | 189 | VPYPQRDMPIQA | none | 2 | 707.860 | 34.71 | 2.8 | 68.9 | x | x | x | 1; 4; 5 |
| 527 | 178 | 189 | VPYPQRDmPIQA | M8(Ox) | 2 | 715.858 | 28.04 | 2.7 | 60.1 | x | x | x | 1 |
| 528 | 178 | 190 | VPYPQRDMPIQAF | none | 2 | 781.395 | 45.14 | 3.2 | 76.2 | x | x | x | |
| 529 | 178 | 191 | VPYPQRDMPIQAFL | none | 2 | 837.938 | 55.53 | 3.2 | 61.7 | x | x | | 1; 2; 5 |
| 530 | 178 | 191 | VPYPQRDmPIQAFL | M8(Ox) | 2 | 845.934 | 48.70 | 2.6 | 35.7 | x | x | | |
| 531 | 180 | 189 | YPQRDmPIQA | M6(Ox) | 2 | 617.797 | 22.13 | 2.0 | 38.9 | x | | | |
| 532 | 180 | 190 | YPQRDMPIQAF | none | 2 | 683.336 | 41.11 | 2.4 | 43.1 | | | x | |
| 533 | 183 | 190 | RDMPIQAF | none | 2 | 489.247 | 36.95 | 1.7 | 40.9 | | | x | |
| 534 | 183 | 190 | RDmPIQAF | M3(Ox) | 2 | 497.245 | 29.32 | 2.0 | 36.6 | | | x | |
| 535 | 191 | 198 | LLYQEPVL | none | 2 | 487.781 | 47.22 | 1.6 | 33.2 | | | x | |
| 536 | 191 | 201 | LLYQEPVLGPV | none | 2 | 614.354 | 51.48 | 2.4 | 48.9 | x | | x | 5 |
| 537 | 191 | 206 | LLYQEPVLGPVRGPFP | none | 2 (3) | 891.506 | 55.05 | 4.2 | 89.3 | | | x | 6; 7 |
| 538 | 191 | 207 | LLYQEPVLGPVRGPFPI | none | 2 (3) | 948.046 | 61.12 | 3.5 | 78.7 | x | x | x | |
| 539 | 191 | 208 | LLYQEPVLGPVRGPFPII | none | 2 (3) | 1004.586 | 65.35 | 4.0 | 80.7 | x | x | x | 2 |
| 540 | 191 | 209 | LLYQEPVLGPVRGPFPIIV | none | 2 (3) | 1054.120 | 67.06 | 4.2 | 89.0 | x | x | x | 1; 5; 6 |
| 541 | 192 | 206 | LYQEPVLGPVRGPFP | none | 2 | 834.960 | 50.72 | 4.0 | 84.4 | | | x | 2; 6 |

| Nr. | Start pos. | End pos. | Sequence | Modification | z | *m/z* | $t_R$ (min) | Xcorr | -10lgP | RM[a] | UHT[a] | IF[a] | Literature (milk[1-6], colostrum[7]) |
|---|---|---|---|---|---|---|---|---|---|---|---|---|---|
| 542 | 192 | 207 | LYQEPVLGPVRGPFPI | none | 2 | 891.503 | 57.40 | 3.2 | 81.7 | x | | x | 2 |
| 543 | 192 | 208 | LYQEPVLGPVRGPFPII | none | 2 | 948.043 | 62.13 | 4.2 | 88.2 | x | x | x | 2; 5; 6 |
| 544 | 192 | 209 | LYQEPVLGPVRGPFPIIV | none | 2 (3) | 997.578 | 63.95 | 3.8 | 82.3 | x | x | x | 1; 4; 5; 6 |
| 545 | 193 | 202 | YQEPVLGPVR | none | 2 | 579.318 | 32.25 | 2.7 | 57.7 | x | x | x | 1; 5; 7 |
| 546 | 193 | 206 | YQEPVLGPVRGPFP | none | 2 | 778.417 | 48.25 | 3.1 | 87.7 | | | x | 1; 6; 7 |
| 547 | 193 | 207 | YQEPVLGPVRGPFPI | none | 2 | 834.961 | 55.25 | 3.8 | 83.1 | x | x | x | 7 |
| 548 | 193 | 208 | YQEPVLGPVRGPFPII | none | 2 | 891.501 | 60.09 | 4.1 | 85.1 | x | x | x | 1; 5 |
| 549 | 193 | 209 | YQEPVLGPVRGPFPIIV | none | 2 (3) | 941.035 | 62.19 | 3.8 | 91.8 | x | x | x | 1; 5; 6 |
| 550 | 194 | 202 | QEPVLGPVR | none | 2 | 497.787 | 26.58 | 2.0 | 41.2 | x | x | | 1 |
| 551 | 194 | 206 | QEPVLGPVRGPFP | none | 2 | 696.884 | 45.81 | 2.8 | 66.0 | x | x | x | 1; 5; 7 |
| 552 | 194 | 208 | QEPVLGPVRGPFPII | none | 2 | 809.969 | 58.45 | 2.9 | 72.1 | x | x | x | 1; 5; 7 |
| 553 | 194 | 209 | QEPVLGPVRGPFPIIV | none | 2 | 859.503 | 60.57 | 3.1 | 77.1 | x | x | x | 1; 5 |
| 554 | 195 | 206 | EPVLGPVRGPFP | none | 2 | 632.855 | 45.94 | 2.5 | 61.7 | x | x | x | 1; 4; 5; 6; 7 |
| 555 | 195 | 207 | EPVLGPVRGPFPI | none | 2 | 689.398 | 53.30 | 2.1 | 47.3 | x | x | x | 1; 5; 7 |
| 556 | 195 | 208 | EPVLGPVRGPFPII | none | 2 | 745.940 | 58.53 | 2.6 | 62.8 | x | x | x | 1; 5 |
| 557 | 195 | 209 | EPVLGPVRGPFPIIV | none | 2 | 795.474 | 60.82 | 2.7 | 66.9 | x | x | x | 1; 2; 4; 5 |
| 558 | 196 | 209 | PVLGPVRGPFPIIV | none | 2 | 730.953 | 60.32 | 3.1 | 65.3 | x | x | x | 1; 3 |
| 559 | 197 | 209 | VLGPVRGPFPIIV | none | 2 | 682.427 | 58.12 | 1.9 | 46.5 | | x | x | |
| 560 | 198 | 209 | LGPVRGPFPIIV | none | 2 | 632.892 | 56.47 | 1.8 | 46.8 | x | x | x | 5 |
| 561 | 199 | 208 | GPVRGPFPII | none | 2 | 526.815 | 48.58 | 2.1 | 46.6 | x | x | x | 1 |
| 562 | 199 | 209 | GPVRGPFPIIV | none | 2 | 576.350 | 51.83 | 1.7 | 43.6 | x | x | x | 1; 4; 5 |
| 563 | 200 | 209 | PVRGPFPIIV | none | 2 | 547.837 | 51.65 | 2.1 | 36.0 | | x | x | 1; 2; 6 |
| **Beta-lactoglobulin (P02754)** | | | | | | | | | | | | | |
| 564 | 1 | 8 | LIVTQTMK | none | 2 | 467.274 | 25.46 | 2.4 | 48.2 | x | x | x | 1; 2 |
| 565 | 1 | 10 | LIVTQTMKGL | none | 2 | 552.328 | 40.43 | 2.7 | 55.7 | | x | x | |
| 566 | 1 | 10 | LIVTQTmKGL | M7(Ox) | 2 | 560.326 | 30.39 | 2.3 | 56.8 | | | x | |
| 567 | 1 | 11 | LIVTQTMKGLD | none | 2 | 609.843 | 37.95 | 3.3 | 71.7 | | x | x | |
| 568 | 1 | 11 | LIVTQTmKGLD | M7(Ox) | 2 | 617.840 | 28.49 | 2.7 | 69.5 | | x | x | |
| 569 | 1 | 13 | LIVTQTMKGLDIQ | none | 2 | 730.415 | 45.62 | 3.6 | 80.9 | | x | x | |

| Nr. | Start pos. | End pos. | Sequence | Modification | z | *m/z* | $t_R$ (min) | Xcorr | -10lgP | RM[a] | UHT[a] | IF[a] | Literature (milk[1-6], colostrum[7]) |
|---|---|---|---|---|---|---|---|---|---|---|---|---|---|
| 570 | 1 | 13 | LIVTQTmKGLDIQ | M7(Ox) | 2 | 738.412 | 37.26 | 3.3 | 72.2 | | | x | |
| 571 | 1 | 17 | LIVTQTMKGLDIQKVAG | none | 2 | 908.025 | 44.70 | 3.4 | 51.7 | | x | x | |
| 572 | 2 | 13 | IVTQTMKGLDIQ | none | 2 | 673.872 | 36.76 | 2.6 | 51.5 | | | x | |
| 573 | 9 | 16 | GLDIQKVA | none | 2 | 422.250 | 29.83 | 2.0 | 37.7 | | | x | |
| 574 | 9 | 22 | GLDIQKVAGTWYSL | none | 2 | 775.914 | 58.01 | 3.2 | 68.3 | | | x | |
| 575 | 11 | 22 | DIQKVAGTWYSL | none | 2 | 690.863 | 49.95 | 3.6 | 60.5 | | | x | |
| 576 | 14 | 22 | KVAGTWYSL | none | 2 | 512.776 | 41.72 | 2.3 | 61.5 | | | x | |
| 577 | 33 | 42 | DAQSAPLRVY | none | 2 | 560.292 | 30.72 | 2.6 | 76.1 | | | x | |
| 578 | 127 | 136 | EVDDEALEKF | none | 2 | 597.779 | 37.48 | 2.0 | 36.0 | x | x | | |
| 579 | 153 | 162 | PTQLEEQCHI | none | 2 | 599.281 | 29.13 | 3.0 | 63.5 | | x | x | |
| **Butyrophilin subfamily 1 member A1 (P18892)** | | | | | | | | | | | | | |
| 580 | 50 | 66 | VSREGQEQEGEEMAEYR | none | 3 (2) | 676.297 | 24.37 | 5.4 | 103.8 | x | x | x | |
| 581 | 50 | 66 | VSREGQEQEGEEmAEYR | M13(Ox) | 3 (2) | 681.629 | 18.02 | 5.3 | 69.8 | x | x | x | |
| 582 | 53 | 66 | EGQEQEGEEMAEYR | none | 2 | 842.840 | 26.18 | 3.7 | 70.2 | x | x | x | |
| 583 | 53 | 66 | EGQEQEGEEmAEYR | M10(Ox) | 2 | 850.838 | 18.52 | 2.7 | 48.9 | x | x | | |
| 584 | 67 | 84 | GRVSLVEDHIAEGSVAVR | none | 3 (4) | 632.009 | 33.19 | 4.0 | 67.8 | x | x | x | |
| 585 | 68 | 84 | RVSLVEDHIAEGSVAVR | none | 3 | 613.001 | 32.41 | 3.7 | 55.1 | x | x | x | |
| 586 | 69 | 84 | VSLVEDHIAEGSVAVR | none | 3 | 560.969 | 35.23 | 4.0 | 65.0 | x | x | x | |
| 587 | 70 | 84 | SLVEDHIAEGSVAVR | none | 3 (2) | 527.945 | 32.36 | 3.9 | 77.3 | x | x | | |
| 588 | 72 | 82 | VEDHIAEGSVA | none | 2 | 563.770 | 22.24 | 2.1 | 47.6 | x | x | x | |
| 589 | 72 | 84 | VEDHIAEGSVAVR | none | 3 (2) | 461.240 | 23.63 | 4.3 | 66.3 | x | x | x | |
| 590 | 85 | 97 | IQEVKASDDGEYR | none | 2 | 755.361 | 19.00 | 3.6 | 63.5 | x | x | | |
| 591 | 102 | 114 | QDENYEEAIVHLK | none | 3 | 529.924 | 37.79 | 3.3 | 35.8 | x | x | | |
| 592 | 153 | 177 | THRGEEFPSMSESRNPDEEGLFTVR | none | 4 (3,5) | 727.589 | 39.91 | 5.9 | 89.2 | x | x | x | 3; 5 |
| 593 | 153 | 177 | THRGEEFPSmSESRNPDEEGLFTVR | M10(Ox) | 4 (3,5) | 731.586 | 34.95 | 5.4 | 54.7 | x | x | x | 3 |
| 594 | 156 | 177 | GEEFPSMSESRNPDEEGLFTVR | none | 3 | 838.380 | 45.95 | 4.8 | 65.2 | x | x | x | |
| 595 | 169 | 177 | DEEGLFTVR | none | 2 | 533.263 | 38.33 | 2.6 | 54.1 | | x | | |
| 596 | 196 | 203 | NLLLGQEK | none | 2 | 457.767 | 27.71 | 2.2 | 31.5 | x | x | | |
| 597 | 287 | 297 | HPHLFLYEDSK | none | 3 | 462.564 | 31.26 | 3.4 | 60.7 | x | x | | |

| Nr. | Start pos. | End pos. | Sequence | Modification | z | *m/z* | $t_R$ (min) | Xcorr | -10lgP | RM[a] | UHT[a] | IF[a] | Literature (milk[1-6], colostrum[7]) |
|---|---|---|---|---|---|---|---|---|---|---|---|---|---|
| 598 | 467 | 500 | KEIPLSPMGEDSASGDIETLHSKLIPLQPSQGVP | none | 3 | 1190.953 | 53.65 | 4.3 | 48.0 | x | x | | |
| 599 | 469 | 487 | IPLSPMGEDSASGDIETLH | none | 2 | 984.967 | 43.78 | 5.5 | 123.4 | x | x | x | 5 |
| 600 | 469 | 487 | IPLSPmGEDSASGDIETLH | M6(Ox) | 2 | 992.963 | 38.58 | 5.0 | 99.5 | x | x | x | |
| 601 | 472 | 487 | SPmGEDSASGDIETLH | M3(Ox) | 2 | 831.350 | 27.44 | 3.3 | 68.5 | x | x | x | |
| 602 | 475 | 487 | GEDSASGDIETLH | none | 2 | 665.789 | 25.58 | 3.7 | 84.0 | x | x | x | |
| **CCAAT/enhancer-binding protein beta (O02755)** | | | | | | | | | | | | | |
| 603 | 161 | 169 | HPPPPPPPP | none | 2 | 466.751 | 18.19 | 2.2 | 48.0 | x | x | | |
| **DNA-directed RNA polymerases I, II, and III subunit RPABC1 (Q2T9T3)** | | | | | | | | | | | | | |
| 604 | 77 | 87 | PEEPKVGIKTI | none | 3 (2) | 404.239 | 29.20 | 3.2 | 53.8 | x | x | | |
| **DnaJ homolog subfamily C member 12 (Q9N287)** | | | | | | | | | | | | | |
| 605 | 185 | 193 | APSELLRKF | none | 2 | 530.811 | 34.61 | 2.8 | 45.6 | x | x | | |
| **Fatty acid synthase (Q71SP7)** | | | | | | | | | | | | | |
| 606 | 379 | 389 | RPLPVLGGNVG | none | 2 | 539.821 | 36.20 | 2.6 | 53.8 | x | x | | |
| 607 | 379 | 393 | RPLPVLGGNVGINSF | none | 2 | 770.435 | 51.61 | 3.1 | 58.6 | x | x | | |
| 608 | 1483 | 1493 | SPIPETDPKSL | none | 2 | 592.313 | 31.30 | 2.4 | 61.1 | x | x | x | |
| 609 | 20 | 38 | EDGSDPPSGDFLTEGGGVR | none | 2 | 946.420 | 37.27 | 3.3 | 87.9 | x | x | x | |
| **Fibroblast growth factor-binding protein 1 (Q9MZ06)** | | | | | | | | | | | | | |
| 610 | 8 | 27 | RGSKASADESLALGKPGKEP | none | 4 | 500.269 | 22.06 | 2.7 | 32.2 | x | x | x | |
| 611 | 8 | 28 | RGSKASADESLALGKPGKEPR | none | 4 (5) | 539.295 | 19.29 | 5.0 | 70.4 | x | x | x | 2; 5 |
| 612 | 9 | 27 | GSKASADESLALGKPGKEP | none | 3 | 614.655 | 24.30 | 4.5 | 59.0 | x | x | x | |
| 613 | 9 | 28 | GSKASADESLALGKPGKEPR | none | 3 (2,4,5) | 666.691 | 20.88 | 3.8 | 55.0 | x | x | x | 2; 3; 5 |
| 614 | 9 | 28 | GSKAsADESLALGKPGKEPR | S5(Phospho) | 4 | 520.261 | 22.73 | 4.1 | 31.4 | x | x | | 3 |
| 615 | 12 | 28 | ASADESLALGKPGKEPR | none | 3 (4) | 575.976 | 22.88 | 3.7 | 55.9 | x | x | x | 5 |
| 616 | 13 | 28 | SADESLALGKPGKEPR | none | 3 | 552.295 | 22.11 | 3.6 | 36.6 | x | x | | |
| 617 | 14 | 28 | ADESLALGKPGKEPR | none | 3 | 523.285 | 21.58 | 3.1 | 29.9 | x | x | | |
| **FXYD domain-containing ion transport regulator 6 (Q3MHZ5)** | | | | | | | | | | | | | |
| 618 | 1 | 20 | SATEQEKEKDPFHYDYQTLR | none | 4 (3) | 622.046 | 32.90 | 3.4 | 42.2 | x | x | x | |
| 619 | 1 | 25 | SATEQEKEKDPFHYDYQTLRIGGLV | none | 3 (4) | 975.486 | 49.06 | 4.6 | 76.6 | x | x | x | |
| **Glycosylation-dependent cell adhesion molecule 1 (P80195)** | | | | | | | | | | | | | |

| Nr. | Start pos. | End pos. | Sequence | Modification | z | *m/z* | $t_R$ (min) | Xcorr | -10lgP | RM[a] | UHT[a] | IF[a] | Literature (milk[1-6], colostrum[7]) |
|---|---|---|---|---|---|---|---|---|---|---|---|---|---|
| 620 | 1 | 11 | ILNKPEDETHL | none | 2 (3) | 654.843 | 25.37 | 3.4 | 73.6 | | x | x | |
| 621 | 1 | 12 | ILNKPEDETHLE | none | 2 (3) | 719.364 | 24.28 | 3.7 | 71.4 | x | x | x | |
| 622 | 1 | 16 | ILNKPEDETHLEAQPT | none | 2 | 917.964 | 26.34 | 3.3 | 71.8 | x | x | x | |
| 623 | 1 | 17 | ILNKPEDETHLEAQPTD | none | 2 | 975.475 | 26.66 | 4.4 | 73.7 | x | x | x | |
| 624 | 1 | 18 | ILNKPEDETHLEAQPTDA | none | 3 (2) | 674.332 | 27.78 | 2.3 | 47.4 | x | x | x | |
| 625 | 1 | 20 | ILNKPEDETHLEAQPTDASA | none | 2 | 1090.030 | 28.01 | 3.7 | 58.1 | x | x | x | 3 |
| 626 | 1 | 21 | ILNKPEDETHLEAQPTDASAQ | none | 2 (3) | 1154.065 | 27.25 | 3.3 | 71.3 | x | x | x | 7 |
| 627 | 1 | 22 | ILNKPEDETHLEAQPTDASAQF | none | 2 (3) | 1227.595 | 35.72 | 4.8 | 105.5 | x | x | x | 3; 5; 7 |
| 628 | 1 | 23 | ILNKPEDETHLEAQPTDASAQFI | none | 2 (3) | 1284.141 | 41.00 | 3.6 | 60.9 | x | x | x | 5 |
| 629 | 1 | 24 | ILNKPEDETHLEAQPTDASAQFIR | none | 3 (2,4) | 908.460 | 36.52 | 6.1 | 99.0 | x | x | x | 3; 5 |
| 630 | 1 | 24 | ILNKPEDETHLEAQPTDAsAQFIR | S19(Phospho) | 3 | 935.114 | 36.44 | 4.9 | 51.7 | x | x | x | |
| 631 | 1 | 25 | ILNKPEDETHLEAQPTDASAQFIRN | none | 3 | 946.474 | 35.84 | 6.2 | 73.4 | x | x | x | 3; 4; 5 |
| 632 | 1 | 26 | ILNKPEDETHLEAQPTDASAQFIRNL | none | 3 (4) | 984.169 | 47.12 | 7.4 | 94.4 | x | x | x | |
| 633 | 1 | 27 | ILNKPEDETHLEAQPTDASAQFIRNLQ | none | 3 (4) | 1026.855 | 44.88 | 6.2 | 86.5 | x | x | x | 5 |
| 634 | 1 | 53 | ILNKPEDETHLEAQPTDASAQFIRNLQISNEDLSKEPSISREDLI SKEQIVIR | none | 6 (5) | 1010.530 | 54.54 | 5.3 | 30.0 | x | x | x | |
| 635 | 2 | 12 | LNKPEDETHLE | none | 3 (2) | 442.217 | 20.37 | 3.2 | 65.4 | x | x | x | |
| 636 | 2 | 22 | LNKPEDETHLEAQPTDASAQF | none | 3 (2) | 781.038 | 33.47 | 4.4 | 71.4 | x | x | x | 7 |
| 637 | 2 | 24 | LNKPEDETHLEAQPTDASAQFIR | none | 4 | 653.324 | 34.72 | 3.8 | 44.2 | x | x | | 5 |
| 638 | 12 | 22 | EAQPTDASAQF | none | 2 | 582.761 | 27.79 | 2.8 | 73.9 | | | x | |
| 639 | 13 | 22 | AQPTDASAQF | none | 2 | 518.240 | 26.40 | 2.6 | 52.4 | | | x | |
| 640 | 13 | 26 | AQPTDASAQFIRNL | none | 2 | 766.397 | 46.18 | 3.1 | 75.8 | | | x | |
| 641 | 44 | 53 | LIsKEQIVIR | S3(Phospho) | 2 | 639.862 | 35.70 | 3.2 | 44.0 | x | x | x | |
| 642 | 54 | 66 | SSRQPQSQNPKLP | none | 3 | 489.594 | 19.71 | 2.9 | 45.8 | x | x | x | |
| 643 | 54 | 67 | SSRQPQSQNPKLPL | none | 3 (2) | 527.289 | 27.77 | 3.5 | 60.7 | x | x | x | |
| 644 | 54 | 68 | SSRQPQSQNPKLPLS | none | 3 (2) | 556.300 | 25.69 | 2.7 | 49.7 | x | x | x | 3; 5; 7 |
| 645 | 54 | 70 | SSRQPQSQNPKLPLSIL | none | 2 (3) | 947.033 | 45.70 | 3.2 | 77.8 | x | x | x | 5; 7 |
| 646 | 54 | 71 | SSRQPQSQNPKLPLSILK | none | 3 (2,4) | 674.387 | 37.70 | 5.4 | 71.1 | x | x | x | 5 |
| 647 | 54 | 72 | SSRQPQSQNPKLPLSILKE | none | 4 (3) | 538.303 | 39.06 | 4.8 | 61.0 | x | x | x | 3; 5 |

| Nr. | Start pos. | End pos. | Sequence | Modification | z | *m/z* | $t_R$ (min) | Xcorr | -10lgP | RM[a] | UHT[a] | IF[a] | Literature (milk[1-6], colostrum[7]) |
|---|---|---|---|---|---|---|---|---|---|---|---|---|---|
| 648 | 54 | 73 | SSRQPQSQNPKLPLSILKEK | none | 4 (3,5) | 570.327 | 34.40 | 5.8 | 59.2 | x | x | x | 2; 3; 5 |
| 649 | 54 | 75 | SSRQPQSQNPKLPLSILKEKHL | none | 4 (3,5) | 632.862 | 36.82 | 5.2 | 55.9 | x | x | x | 2; 3; 5 |
| 650 | 55 | 68 | SRQPQSQNPKLPLS | none | 3 (2) | 527.289 | 25.48 | 2.8 | 38.5 | x | x | x | 3; 5 |
| 651 | 55 | 70 | SRQPQSQNPKLPLSIL | none | 3 | 602.680 | 45.70 | 3.0 | 24.8 | x | x | x | |
| 652 | 55 | 71 | SRQPQSQNPKLPLSILK | none | 4 (3) | 484.284 | 37.75 | 4.1 | 60.2 | x | x | x | |
| 653 | 57 | 68 | QPQSQNPKLPLS | none | 2 | 668.864 | 28.63 | 2.1 | 55.3 | x | x | x | 5 |
| 654 | 57 | 70 | QPQSQNPKLPLSIL | none | 2 | 781.948 | 50.34 | 1.9 | 52.9 | x | x | x | 5 |
| 655 | 57 | 71 | QPQSQNPKLPLSILK | none | 3 | 564.333 | 41.21 | 1.8 | 30.6 | x | x | | |
| 656 | 57 | 73 | QPQSQNPKLPLSILKEK | none | 3 (4) | 650.046 | 37.29 | 3.1 | 26.5 | x | x | x | 5 |
| 657 | 57 | 75 | QPQSQNPKLPLSILKEKHL | none | 3 (5) | 733.426 | 39.24 | 2.3 | 37.1 | x | x | | 5 |
| 658 | 60 | 67 | SQNPKLPL | none | 2 | 448.763 | 30.54 | 2.1 | 42.0 | | | x | |
| 659 | 60 | 68 | SQNPKLPLS | none | 2 | 492.279 | 27.81 | 2.0 | 45.6 | | | x | |
| 660 | 60 | 70 | SQNPKLPLSIL | none | 2 | 605.364 | 50.30 | 2.4 | 51.9 | | | x | 7 |
| 661 | 62 | 70 | NPKLPLSIL | none | 2 | 497.818 | 51.03 | 2.2 | 35.4 | | | x | |
| 662 | 63 | 70 | PKLPLSIL | none | 2 | 440.797 | 50.94 | 1.9 | 25.4 | | | x | |
| 663 | 65 | 73 | LPLSILKEK | none | 2 | 520.839 | 35.61 | 1.8 | 43.7 | x | x | | 3 |
| 664 | 65 | 74 | LPLSILKEKH | none | 2 | 589.368 | 31.08 | 2.2 | 47.5 | x | x | | |
| 665 | 65 | 75 | LPLSILKEKHL | none | 2 (3) | 645.910 | 37.83 | 3.5 | 55.1 | x | x | x | |
| 666 | 109 | 121 | RNLENTVKETIKY | none | 3 (2) | 536.629 | 32.85 | 4.0 | 61.3 | | x | x | |
| 667 | 111 | 121 | LENTVKETIKY | none | 2 (3) | 669.369 | 27.69 | 3.2 | 66.8 | | | x | |
| 668 | 112 | 121 | ENTVKETIKY | none | 2 (3) | 612.827 | 23.24 | 2.9 | 47.2 | | | x | |
| 669 | 113 | 121 | NTVKETIKY | none | 2 | 548.305 | 21.05 | 2.3 | 46.5 | | | x | |
| 670 | 122 | 135 | LKSLFSHAFEVVKT | none | 3 (4) | 535.972 | 43.57 | 3.2 | 70.5 | | | x | |
| 671 | 123 | 135 | KSLFSHAFEVVKT | none | 3 | 498.276 | 39.18 | 3.5 | 50.7 | | | x | |
| 672 | 124 | 135 | SLFSHAFEVVKT | none | 2 | 682.864 | 46.16 | 3.2 | 79.8 | | x | x | |
| 673 | 126 | 135 | FSHAFEVVKT | none | 2 | 582.805 | 30.31 | 2.6 | 73.2 | | | x | |
| 674 | 127 | 135 | SHAFEVVKT | none | 2 | 509.270 | 22.60 | 2.9 | 73.2 | x | x | x | 3; 5 |
| 675 | 128 | 135 | HAFEVVKT | none | 2 | 465.755 | 21.45 | 2.2 | 55.9 | x | x | x | |
| **Kappa-casein (P02668)** | | | | | | | | | | | | | |

| Nr. | Start pos. | End pos. | Sequence | Modification | z | *m/z* | $t_R$ (min) | Xcorr | -10lgP | RM[a] | UHT[a] | IF[a] | Literature (milk[1-6], colostrum[7]) |
|---|---|---|---|---|---|---|---|---|---|---|---|---|---|
| 676 | 14 | 24 | DERFFSDKIAK | none | 3 | 452.569 | 29.31 | 3.4 | 41.0 | x | x | x | |
| 677 | 31 | 38 | VLSRYPSY | none | 2 | 492.761 | 26.80 | 2.4 | 58.3 | | | x | |
| 678 | 31 | 40 | VLSRYPSYGL | none | 2 | 577.813 | 36.76 | 2.7 | 65.7 | | | x | |
| 679 | 31 | 41 | VLSRYPSYGLN | none | 2 | 634.836 | 32.74 | 3.1 | 73.5 | | | x | |
| 680 | 31 | 43 | VLSRYPSYGLNYY | none | 2 | 797.901 | 42.57 | 2.8 | 65.6 | | | x | |
| 681 | 33 | 40 | SRYPSYGL | none | 2 | 471.736 | 30.91 | 2.2 | 47.8 | | | x | |
| 682 | 33 | 41 | SRYPSYGLN | none | 2 | 528.759 | 26.99 | 2.5 | 62.1 | | | x | |
| 683 | 33 | 43 | SRYPSYGLNYY | none | 2 | 691.824 | 38.76 | 3.4 | 63.3 | | | x | |
| 684 | 41 | 50 | NYYQQKPVAL | none | 2 | 612.325 | 31.29 | 2.8 | 67.2 | | | x | |
| 685 | 42 | 50 | YYQQKPVAL | none | 2 | 555.303 | 28.59 | 2.7 | 58.1 | | | x | 7 |
| 686 | 42 | 53 | YYQQKPVALINN | none | 2 | 725.889 | 31.23 | 3.4 | 50.9 | | | x | |
| 687 | 43 | 50 | YQQKPVAL | none | 2 | 473.770 | 24.17 | 2.4 | 47.3 | | | x | |
| 688 | 43 | 53 | YQQKPVALINN | none | 2 | 644.356 | 27.79 | 3.2 | 45.1 | | | x | |
| 689 | 49 | 65 | ALINNQFLPYPYYAKPA | none | 2 | 992.023 | 53.06 | 3.4 | 55.7 | x | x | | 5 |
| 690 | 51 | 65 | INNQFLPYPYYAKPA | none | 2 | 899.963 | 46.66 | 4.2 | 65.6 | | | x | |
| 691 | 52 | 65 | NNQFLPYPYYAKPA | none | 2 | 843.421 | 44.60 | 2.9 | 59.9 | | | x | |
| 692 | 66 | 74 | AVRSPAQIL | none | 2 | 477.790 | 33.35 | 2.2 | 36.9 | | | x | |
| 693 | 66 | 76 | AVRSPAQILQW | none | 2 | 634.860 | 49.07 | 2.1 | 49.7 | | | x | |
| 694 | 69 | 79 | SPAQILQWQVL | none | 2 | 641.862 | 61.49 | 1.6 | 54.8 | x | | | 5 |
| 695 | 106 | 137 | MAIPPKKNQDKTEIPTINTIASGEPTSTPTTE | none | 4 | 853.441 | 38.57 | 2.9 | 31.8 | | | x | |
| 696 | 106 | 137 | mAIPPKKNQDKTEIPTINTIASGEPTSTPTTE | M1(Ox) | 4 | 857.439 | 37.24 | 4.1 | 25.9 | | | x | |
| 697 | 106 | 169 | MAIPPKKNQDKTEIPTINTIASGEPTSTPTTEAVESTVATLEDsP EVIESPPEINTVQVTSTAV | S44(Phospho) | 4 | 1696.848 | 62.56 | 3.8 | 26.3 | | | x | |
| 698 | 114 | 123 | QDKTEIPTIN | none | 2 | 579.803 | 29.01 | 2.1 | 41.2 | | | x | |
| 699 | 148 | 169 | DsPEVIESPPEINTVQVTSTAV | S2(Phospho) | 2 | 1196.565 | 53.71 | 3.8 | 59.2 | | x | x | |
| 700 | 149 | 169 | SPEVIESPPEINTVQVTSTAV | none | 2 | 1099.067 | 48.66 | 4.2 | 98.1 | x | x | x | |
| 701 | 149 | 169 | sPEVIESPPEINTVQVTSTAV | S1(Phospho) | 2 | 1139.050 | 52.01 | 4.3 | 82.7 | x | x | x | |
| 702 | 150 | 169 | PEVIESPPEINTVQVTSTAV | none | 2 | 1055.551 | 48.60 | 4.1 | 90.8 | x | x | x | |
| 703 | 151 | 169 | EVIESPPEINTVQVTSTAV | none | 2 | 1007.024 | 47.36 | 3.1 | 68.6 | | x | x | |

| Nr. | Start pos. | End pos. | Sequence | Modification | z | *m/z* | $t_R$ (min) | Xcorr | -10lgP | RM[a] | UHT[a] | IF[a] | Literature (milk[1-6], colostrum[7]) |
|---|---|---|---|---|---|---|---|---|---|---|---|---|---|
| 704 | 152 | 160 | VIESPPEIN | none | 2 | 499.263 | 30.82 | 2.5 | 62.2 | | | x | |
| 705 | 152 | 161 | VIESPPEINT | none | 2 | 549.787 | 33.18 | 2.4 | 56.9 | x | | x | 7 |
| 706 | 152 | 169 | VIESPPEINTVQVTSTAV | none | 2 | 942.502 | 45.84 | 2.9 | 64.9 | | x | x | |
| 707 | 155 | 169 | SPPEINTVQVTSTAV | none | 2 | 771.905 | 40.01 | 2.3 | 65.2 | | x | x | |
| 708 | 159 | 169 | INTVQVTSTAV | none | 2 | 566.813 | 33.08 | 2.4 | 57.2 | | | x | 1; 7 |
| 709 | 161 | 169 | TVQVTSTAV | none | 2 | 453.249 | 25.67 | 2.6 | 51.5 | | x | x | 3 |
| **Lactoperoxidase (P80025)** | | | | | | | | | | | | | |
| 710 | 1 | 20 | DTIAQAASTTTISDAVSKVK | none | 2 (3) | 1004.034 | 42.63 | 4.3 | 73.2 | x | x | x | |
| 711 | 35 | 46 | KTTLSSEAPTTQ | none | 2 | 632.325 | 17.99 | 2.4 | 63.4 | | | x | |
| 712 | 37 | 53 | TLSSEAPTTQQLSEYFK | none | 2 | 965.476 | 47.25 | 5.0 | 93.2 | x | x | | |
| 713 | 39 | 51 | SSEAPTTQQLSEY | none | 2 | 720.829 | 32.42 | 2.6 | 75.0 | | | x | |
| 714 | 40 | 51 | SEAPTTQQLSEY | none | 2 | 677.312 | 32.40 | 2.1 | 47.2 | | | x | |
| 715 | 61 | 74 | TAIRNGQVWEESLK | none | 3 | 544.289 | 32.63 | 3.4 | 55.2 | x | x | x | 5 |
| 716 | 61 | 76 | TAIRNGQVWEESLKRL | none | 4 (3) | 475.765 | 41.47 | 5.2 | 61.8 | x | x | x | |
| 717 | 62 | 74 | AIRNGQVWEESLK | none | 3 | 510.606 | 31.57 | 3.9 | 40.9 | x | x | x | 3 |
| 718 | 62 | 76 | AIRNGQVWEESLKRL | none | 4 | 450.502 | 40.52 | 3.3 | 24.9 | x | x | x | |
| 719 | 65 | 74 | NGQVWEESLK | none | 2 | 595.296 | 32.68 | 2.1 | 53.4 | x | x | x | 3 |
| 720 | 66 | 74 | GQVWEESLK | none | 2 | 538.274 | 32.27 | 2.8 | 53.1 | x | x | x | 5 |
| 721 | 66 | 76 | GQVWEESLKRL | none | 2 (3) | 672.866 | 41.64 | 3.2 | 57.6 | x | x | x | |
| 722 | 67 | 74 | QVWEESLK | none | 2 | 509.763 | 30.34 | 1.6 | 38.3 | x | x | x | |
| 723 | 67 | 76 | QVWEESLKRL | none | 2 (3) | 429.906 | 39.35 | 1.8 | 54.1 | x | x | x | 2 |
| 724 | 68 | 76 | VWEESLKRL | none | 2 | 580.327 | 35.46 | 2.4 | 53.4 | x | x | x | |
| **Wiskott-Aldrich syndrome protein family member 2 (A2VDK6)** | | | | | | | | | | | | | |
| 725 | 384 | 393 | APPPPPPPPP | none | 2 | 482.267 | 24.04 | 2.8 | 45.9 | x | x | | |
| 726 | 385 | 393 | PPPPPPPPP | none | 2 | 446.748 | 23.87 | 2.1 | 41.1 | x | x | x | |
| 727 | 385 | 393 | PPPPPPPPPP | none | 2 | 495.274 | 25.65 | 2.4 | 41.2 | x | x | x | |
| **Mucin-1 (Q8WML4)** | | | | | | | | | | | | | |
| 728 | 532 | 545 | SPYEEVSAGNGGSN | none | 2 | 684.288 | 22.52 | 2.5 | 41.8 | x | x | | |
| **Mucin-15 (Q8MI01)** | | | | | | | | | | | | | |

| Nr. | Start pos. | End pos. | Sequence | Modification | z | *m/z* | $t_R$ (min) | Xcorr | -10lgP | RM[a] | UHT[a] | IF[a] | Literature (milk[1-6], colostrum[7]) |
|---|---|---|---|---|---|---|---|---|---|---|---|---|---|
| 729 | 246 | 257 | RLYDDRNEPVLR | none | 3 | 515.942 | 25.09 | 2.6 | 36.3 | x | x | | |
| 730 | 247 | 257 | LYDDRNEPVLR | none | 2 (3) | 695.359 | 26.02 | 2.3 | 47.0 | x | x | | |
| 731 | 248 | 257 | YDDRNEPVLR | none | 3 | 426.213 | 21.04 | 2.7 | 36.8 | x | x | x | |
| **Myosin regulatory light polypeptide 9 (Q5E9E2)** | | | | | | | | | | | | | |
| 732 | 97 | 105 | TDPEDVIRN | none | 2 | 529.758 | 24.72 | 1.8 | 45.4 | x | x | | |
| 733 | 98 | 105 | DPEDVIRN | none | 2 | 479.234 | 22.25 | 2.5 | 47.1 | x | x | | |
| **Osteopontin (P31096)** | | | | | | | | | | | | | |
| 734 | 20 | 34 | YPDAVATWLKPDPSQ | none | 2 | 844.419 | 47.19 | 3.6 | 69.4 | x | | | |
| 735 | 249 | 262 | RISHELDSASSEVN | none | 2 | 772.372 | 21.43 | 2.7 | 77.0 | x | x | x | 3; 5 |
| 736 | 249 | 262 | RISHELDSAsSEVN | S10(Phospho) | 2 | 812.352 | 21.84 | 2.8 | 45.4 | x | x | x | |
| 737 | 249 | 262 | RISHELDsASSEVN | S8(Phospho) | 2 | 812.354 | 23.41 | 3.3 | 50.7 | x | x | x | |
| 738 | 250 | 262 | ISHELDSASSEVN | none | 2 | 694.319 | 22.59 | 3.2 | 64.1 | x | x | x | 3 |
| 739 | 250 | 262 | ISHELDsASSEVN | S7(Phospho) | 2 | 734.304 | 25.27 | 3.3 | 54.3 | x | x | x | |
| 740 | 250 | 262 | IRIsHELDSASSEVN | S4(Phospho) | 2 | 868.896 | 34.04 | 3.8 | 57.5 | x | x | x | |
| 741 | 250 | 262 | IRISHELDsASSEVN | S9(Phospho) | 2 | 868.896 | 30.96 | 3.7 | 54.7 | x | x | x | |
| 742 | 248 | 262 | IRISHELDSAsSEVN | S11(Phospho) | 2 | 868.896 | 29.61 | 3.2 | 52.5 | x | x | x | |
| 743 | 251 | 262 | SHELDSASSEVN | none | 2 | 637.777 | 18.34 | 2.9 | 56.8 | x | x | | |
| 744 | 252 | 262 | HELDSASSEVN | none | 2 | 594.261 | 16.57 | 2.0 | 45.0 | x | x | x | |
| **Parathyroid hormone-related protein (P58073)** | | | | | | | | | | | | | |
| 745 | 128 | 153 | VAGTGLEEDYLSDISATsLELNSRRH | S18(Phospho) | 3 (4) | 971.795 | 49.90 | 5.1 | 30.3 | | | x | |
| **Perilipin-2 (Q9TUM6)** | | | | | | | | | | | | | |
| 746 | 7 | 15 | EPQLSVVTR | none | 2 | 514.789 | 27.53 | 1.7 | 35.8 | x | x | | 5 |
| 747 | 66 | 76 | LPIIQKLEPQIA | none | 2 | 681.920 | 47.82 | 3.4 | 61.0 | x | x | x | 5 |
| 748 | 191 | 200 | LPLTKDELEK | none | 2 | 593.338 | 26.60 | 2.5 | 56.2 | x | | | |
| 749 | 431 | 459 | HPKPVPVSNAEGSQPDDSSS | none | 3 (2) | 678.982 | 17.44 | 3.8 | 49.3 | x | x | x | |
| **Poly(rC)-binding protein 1 (Q5E9A3)** | | | | | | | | | | | | | |
| 750 | 317 | 327 | NPVEGSSGRQV | none | 2 | 565.281 | 15.67 | 2.5 | 34.4 | x | x | | |
| **Polymeric immunoglobulin receptor (P81265)** | | | | | | | | | | | | | |
| 751 | 555 | 567 | QVKAAPAGAAIQS | none | 2 | 606.339 | 19.41 | 2.3 | 53.7 | x | x | x | |

| Nr. | Start pos. | End pos. | Sequence | Modification | z | *m/z* | $t_R$ (min) | Xcorr | -10lgP | RM[a] | UHT[a] | IF[a] | Literature (milk[1-6], colostrum[7]) |
|---|---|---|---|---|---|---|---|---|---|---|---|---|---|
| 752 | 556 | 567 | VKAAPAGAAIQS | none | 2 | 542.310 | 18.18 | 2.1 | 45.5 | x | | | |
| 753 | 558 | 568 | AAPAGAAIQSR | none | 2 | 506.778 | 15.50 | 3.0 | 53.9 | x | x | | |
| 754 | 558 | 574 | AAPAGAAIQSRAGEIQN | none | 3 (2) | 542.283 | 25.58 | 3.5 | 84.6 | x | x | x | 7 |
| 755 | 558 | 575 | AAPAGAAIQSRAGEIQNK | none | 2 (3) | 876.970 | 23.89 | 4.5 | 90.0 | x | x | x | 5 |
| 756 | 559 | 573 | APAGAAIQSRAGEIQ | none | 2 | 720.382 | 25.55 | 4.5 | 78.3 | x | x | x | |
| 757 | 559 | 574 | APAGAAIQSRAGEIQN | none | 2 | 777.405 | 24.43 | 4.4 | 82.8 | x | x | x | |
| 758 | 559 | 575 | APAGAAIQSRAGEIQNK | none | 3 | 561.303 | 22.72 | 2.8 | 45.7 | x | x | | |
| 759 | 569 | 579 | AGEIQNKALLD | none | 2 | 586.319 | 27.50 | 2.7 | 58.2 | x | x | x | |
| 760 | 569 | 583 | AGEIQNKALLDPSFF | none | 2 | 825.432 | 53.46 | 2.5 | 49.1 | | | x | |
| 761 | 569 | 613 | AGEIQNKALLDPSFFAKESVKDAAGGPGAPADPGRPTGYSGS SKA | none | 4 (5,6) | 1105.058 | 44.24 | 4.2 | 38.5 | x | x | x | |
| 762 | 569 | 617 | AGEIQNKALLDPSFFAKESVKDAAGGPGAPADPGRPTGYSGS SKALVST | none | 5 | 964.290 | 46.64 | 3.6 | 47.3 | x | x | x | |
| 763 | 576 | 600 | ALLDPSFFAKESVKDAAGGPGAPAD | none | 3 | 811.077 | 50.07 | 3.3 | 58.5 | x | x | x | |
| 764 | 576 | 605 | ALLDPSFFAKESVKDAAGGPGAPADPGRPT | none | 4 | 735.630 | 47.84 | 4.7 | 64.3 | | | x | |
| 765 | 576 | 613 | ALLDPSFFAKESVKDAAGGPGAPADPGRPTGYSGSSKA | none | 3 (4) | 1226.281 | 45.69 | 5.3 | 54.9 | x | x | x | 5 |
| 766 | 576 | 617 | ALLDPSFFAKESVKDAAGGPGAPADPGRPTGYSGSSKALVST | none | 4 | 1020.021 | 48.33 | 4.1 | 31.9 | x | x | x | |
| 767 | 576 | 622 | ALLDPSFFAKESVKDAAGGPGAPADPGRPTGYSGSSKALVST LVPLA | none | 4 (3) | 1143.353 | 59.10 | 3.9 | 29.9 | x | x | x | |
| 768 | 582 | 613 | FFAKESVKDAAGGPGAPADPGRPTGYSGSSKA | none | 4 (3,5) | 770.881 | 28.49 | 3.9 | 62.4 | x | x | x | 3; 5 |
| 769 | 583 | 613 | FAKESVKDAAGGPGAPADPGRPTGYSGSSKA | none | 4 (3) | 734.112 | 24.09 | 4.6 | 59.5 | x | x | x | 3 |
| 770 | 587 | 605 | SVKDAAGGPGAPADPGRPT | none | 3 | 574.291 | 19.15 | 3.8 | 64.9 | | | x | |
| 771 | 587 | 610 | SVKDAAGGPGAPADPGRPTGYSGS | none | 3 | 724.682 | 22.81 | 4.1 | 59.2 | | | x | |
| 772 | 590 | 605 | DAAGGPGAPADPGRPT | none | 2 | 703.836 | 19.04 | 3.0 | 70.2 | | | x | |
| 773 | 590 | 622 | DAAGGPGAPADPGRPTGYSGSSKALVSTLVPLA | none | 3 | 1013.193 | 50.88 | 3.0 | 44.0 | x | x | x | |
| 774 | 591 | 605 | AAGGPGAPADPGRPT | none | 2 | 646.322 | 17.50 | 2.6 | 60.8 | | | x | |
| 775 | 591 | 613 | AAGGPGAPADPGRPTGYSGSSKA | none | 3 | 676.995 | 20.62 | 1.8 | 32.4 | x | x | x | |
| 776 | 601 | 614 | PGRPTGYSGSSKAL | none | 3 | 459.908 | 19.31 | 4.2 | 71.3 | | x | x | |
| 777 | 601 | 622 | PGRPTGYSGSSKALVSTLVPLA | none | 3 | 720.067 | 47.96 | 1.5 | 39.5 | x | x | x | |
| **Protein canopy homolog 3 (Q0P5N1)** | | | | | | | | | | | | | |

| Nr. | Start pos. | End pos. | Sequence | Modification | z | *m/z* | $t_R$ (min) | Xcorr | -10lgP | RM[a] | UHT[a] | IF[a] | Literature (milk[1-6], colostrum[7]) |
|---|---|---|---|---|---|---|---|---|---|---|---|---|---|
| 778 | 31 | 41 | LPAPELGPRQA | none | 2 | 574.823 | 30.01 | 2.6 | 55.2 | x | x | x | |
| **Protein OS-9 (Q3MHX6)** | | | | | | | | | | | | | |
| 779 | 3 | 16 | VGSLNLEELSEMRY | none | 2 | 820.406 | 53.49 | 3.0 | 58.0 | | | x | |
| **Ribonuclease pancreatic (P61823)** | | | | | | | | | | | | | |
| 780 | 114 | 124 | PYVPVHFDASV | none | 2 | 615.811 | 43.25 | 2.7 | 71.7 | | x | x | |
| **Secretoglobin family 1D member (A0JNP2)** | | | | | | | | | | | | | |
| 781 | 72 | 81 | VKAVLNPSSA | none | 2 | 493.287 | 22.60 | 2.3 | 42.5 | | x | | |
| **Serum albumin (P02769)** | | | | | | | | | | | | | |
| 782 | 408 | 415 | FQNALIVR | none | 2 | 480.785 | 33.47 | 2.3 | 43.3 | x | | | |
| 783 | 419 | 433 | KVPQVSTPTLVEVSR | none | 3 (2) | 547.317 | 35.69 | 4.3 | 72.5 | x | | | |
| 784 | 99 | 112 | DPNHFRPAGLPDKY | none | 3 | 542.938 | 31.70 | 3.1 | 45.9 | x | x | | |
| **Sodium-dependent phosphate transport protein 2B (Q27960)** | | | | | | | | | | | | | |
| 785 | 3 | 26 | PWPELENSQPTSEKYTVKADGEQS | none | 3 | 907.428 | 37.29 | 3.0 | 55.0 | x | x | | |
| 786 | 4 | 16 | WPELENSQPTSEK | none | 2 | 772.866 | 33.22 | 3.4 | 63.6 | x | x | x | |
| 787 | 4 | 25 | WPELENSQPTSEKYTVKADGEQ | none | 3 | 846.067 | 36.08 | 3.9 | 56.8 | x | x | x | |
| 788 | 4 | 26 | WPELENSQPTSEKYTVKADGEQS | none | 3 | 875.076 | 35.82 | 5.2 | 68.4 | x | x | x | |
| 789 | 6 | 26 | ELENSQPTSEKYTVKADGEQS | none | 3 | 780.700 | 23.64 | 3.1 | 34.6 | x | x | x | |
| 790 | 26 | 53 | SAKPEKAKETEKDDTGTPITKIELVPSH | none | 4 (5) | 763.158 | 30.78 | 5.2 | 52.7 | x | x | x | |
| 791 | 27 | 53 | AKPEKAKETEKDDTGTPITKIELVPSH | none | 3 (4,5) | 988.201 | 30.74 | 4.8 | 60.1 | x | x | x | |
| 792 | 31 | 53 | KAKETEKDDTGTPITKIELVPSH | none | 4 | 635.090 | 32.21 | 5.3 | 57.7 | x | x | x | |
| 793 | 39 | 47 | DTGTPITKI | none | 2 | 473.265 | 29.26 | 2.2 | 50.3 | x | x | x | |
| 794 | 42 | 53 | TPITKIELVPSH | none | 3 | 445.594 | 35.58 | 3.6 | 62.2 | x | x | x | |
| 795 | 666 | 693 | MDKEAQDGVTKSEVDASGTKIVSSVTAL | none | 3 | 956.151 | 44.54 | 5.0 | 51.1 | x | x | x | |
| 796 | 666 | 693 | mDKEAQDGVTKSEVDASGTKIVSSVTAL | M1(Ox) | 3 | 961.483 | 44.03 | 4.0 | 54.0 | x | x | | |
| 797 | 667 | 683 | DKEAQDGVTKSEVDASG | none | 2 | 868.401 | 17.60 | 2.5 | 52.0 | x | x | | |
| 798 | 667 | 685 | DKEAQDGVTKSEVDASGTK | none | 3 | 655.651 | 16.50 | 2.3 | 36.5 | x | x | | |
| 799 | 667 | 693 | DKEAQDGVTKSEVDASGTKIVSSVTAL | none | 3 | 912.470 | 44.67 | 4.9 | 50.8 | x | x | x | |
| **Syndecan-2 (Q58DD4)** | | | | | | | | | | | | | |
| 800 | 99 | 120 | KTDPAEEDTNVYTEKHSDNLFK | none | 4 | 646.055 | 29.65 | 4.5 | 33.4 | x | x | | |

| Nr. | Start pos. | End pos. | Sequence | Modification | z | *m/z* | $t_R$ (min) | Xcorr | -10lgP | RM[a] | UHT[a] | IF[a] | Literature (milk[1-6], colostrum[7]) |
|---|---|---|---|---|---|---|---|---|---|---|---|---|---|
| **Xanthine dehydrogenase/oxidase (P80457)** | | | | | | | | | | | | | |
| 801 | 563 | 575 | PNGQsKEDTVGRP | S5(Phospho) | 2 | 732.827 | 15.44 | 2.5 | 32.1 | x | x | | |

*Phosphorylation sites rely on sequences suggested by Proteome Discoverer 2.2 and PEAKS.

[a] It should be noted that the acquired LC-MS/MS data sets for the different milk types were not statistically different in terms of number of tandem mass spectra acquired per replicate (ranging from 2359 to 2735, with an average of 2541). Additionally, the overall peak area sum (sum of peak areas of all peptides in a sample replicate) was not significantly different between the samples ($p > 0.09$).


(1) Baum, F., Fedorova, M., Ebner, J., Hoffmann, R., Pischetsrieder, M. (2013): Analysis of the endogenous peptide profile of milk: identification of 248 mainly casein-derived peptides. Journal of proteome research, 12, 5447–5462. doi:10.1021/pr4003273.

(2) Mansor, R.; Mullen, W.; Albalat, A.; Zerefos, P.; Mischak, H.; Barrett, D.C. et al. (2013): A peptidomic approach to biomarker discovery for bovine mastitis. In: J. Proteomics 85, S. 89–98. DOI: 10.1016/j.jprot.2013.04.027.

(3) Dallas, D.C.; Guerrero, A.; Parker, E. A.; Garay, L.A.; Bhandari, A.; Lebrilla, C.B. et al. (2014): Peptidomic profile of milk of Holstein cows at peak lactation. In: J. Agric. Food Chem. 62 (1), S. 58–65. DOI: 10.1021/jf4040964.

(4) Sassi, M.; Arena, S.; Scaloni, A. (2015): MALDI-TOF-MS Platform for Integrated Proteomic and Peptidomic Profiling of Milk Samples Allows Rapid Detection of Food Adulterations. In: J. Agric. Food Chem. 63 (27), S. 6157–6171. DOI: 10.1021/acs.jafc.5b02384.

(5) Guerrero, A.; Dallas, D.C.; Contreras, S.; Bhandari, A.; Cánovas, A.; Islas-Trejo, A. et al. (2015): Peptidomic analysis of healthy and subclinically mastitic bovine milk. In: Int. Dairy J. 46, S. 46–52. DOI: 10.1016/j.idairyj.2014.09.006.

(6) Dalabasmaz, S.; Dittrich, D.; Kellner, I.; Drewello, T.; Pischetsrieder, M. (2019): Identification of peptides reflecting the storage of UHT milk by MALDI-TOF-MS peptide profiling. In: J. Proteomics 207, S. 103444. DOI: 10.1016/j.jprot.2019.103444.

(7) Dallas, D.C.; Weinborn, V.; Moura Bell, J.M.L.N. de; Wang, M.; Parker, E.A.; Guerrero, A. et al. (2014): Comprehensive peptidomic and glycomic evaluation reveals that sweet whey permeate from colostrum is a source of milk protein-derived peptides and oligosaccharides. In: Food Res. Int. 63 (Pt B), S. 203–209. DOI: 10.1016/j.foodres.2014.03.021.

**Table S3:** Modified peptides identified by Proteome Discoverer 2.2 and manual confirmation of the modification sites. If the modified peptide was additionally proposed by PEAKS Studio 10.5, the "-10lgP" value is listed. An "x" indicates that the peptides was present in RM, UHT milk or IF, whereas a "blank" indicates that it was not detected.

| Nr. | *Start pos.* | *End pos.* | *Mod. Site* | Annotated Sequence | Modifications | z | *m/z* | $t_R$ (min) | XCorr | -10lgP | RM | UHT | IF |
|---|---|---|---|---|---|---|---|---|---|---|---|---|---|
| **Alpha-S1-casein (P02662)** | | | | | | | | | | | | | |
| 1 | *1* | *13* | *K7* | RPKHPIkHQGLPQ | K7(Lac) | 4 | 465.754 | 13.13 | 1.8 | | | | x |
| 2 | *1* | *16* | *K7* | RPKHPIkHQGLPQEVL | K7(Lac) | 4 | 551.053 | 22.65 | 3.0 | 18.8 | | | x |
| 3 | *1* | *20* | *K7* | RPKHPIkHQGLPQEVLNENL | K7(Lac) | 5 | 535.087 | 31.77 | 2.8 | | | x | x |
| 4 | *1* | *21* | *K3* | RPkHPIKHQGLPQEVLNENLL | K3(Lac) | 5 | 557.704 | 37.90 | 2.3 | 27.1 | x | x | x |
| 5 | *1* | *22* | *K3* | RPkHPIKHQGLPQEVLNENLLR | K3(Lac) | 4 | 735.904 | 34.36 | 2.2 | 29.1 | x | x | x |
| 6 | *1* | *22* | *K3, K7* | RPkHPIkHQGLPQEVLNENLLR | K3(Lac); K7(Lac) | 5 | 653.747 | 34.04 | 4.8 | 28.0 | | | x |
| 7 | *1* | *22* | *K7* | RPKHPIkHQGLPQEVLNENLLR | K7(Lac) | 5 | 588.926 | 34.43 | 5.4 | 13.9 | x | x | x |
| 8 | *1* | *23* | *K3* | RPkHPIKHQGLPQEVLNENLLRF | K3(Lac) | 5 (4) | 618.339 | 44.04 | 5.0 | 28.0 | x | x | x |
| 9 | *1* | *23* | *K3, K7* | RPkHPIkHQGLPQEVLNENLLRF | K3(Lac); K7(Lac) | 5 | 683.161 | 43.40 | 4.1 | | | | x |
| 10 | *1* | *23* | *K7* | RPKHPIkHQGLPQEVLNENLLRF | K7(Lac) | 5 | 618.339 | 44.17 | 4.4 | | | | x |
| 11 | *23* | *36* | *K36* | FFVAPFPEVFGKEk | K14(Lac) | 3 | 655.999 | 53.52 | 2.5 | 28.2 | | x | x |
| 12 | *23* | *37* | *K36* | FFVAPFPEVFGKEkV | K14(Lac) | 3 | 689.020 | 55.98 | 3.0 | 37.2 | | | x |
| 13 | *23* | *42* | *K36* | FFVAPFPEVFGKEkVNELSK | K14(Lac) | 4 | 659.842 | 52.29 | 4.0 | 37.7 | | | x |
| 14 | *24* | *36* | *K36* | FVAPFPEVFGKEk | K13(Hex) | 3 | 552.956 | 47.39 | 2.8 | 28.0 | | | x |
| 15 | *24* | *38* | *K34* | FVAPFPEVFGkEKVN | K11(Lac) | 3 | 678.012 | 47.61 | 3.2 | | | | x |
| 16 | *24* | *39* | *K34* | FVAPFPEVFGkEKVNE | K11(Lac) | 3 | 721.026 | 47.67 | 2.5 | 21.6 | | | x |
| 17 | *24* | *41* | *K36* | FVAPFPEVFGKEkVNELS | K13(Hex) | 3 | 733.715 | 51.70 | 2.6 | | | | x |
| 18 | *24* | *42* | *K34, K36* | FVAPFPEVFGkEkVNELSK | K11(Hex); K13(Lac) | 4 | 663.588 | 46.51 | 3.6 | 24.3 | | | x |
| 19 | *24* | *42* | *K34, K36* | FVAPFPEVFGkEkVNELSK | K11(Lac); K13(Lac) | 4 | 704.101 | 46.31 | 3.5 | 39.6 | | | x |
| 20 | *24* | *42* | *K36* | FVAPFPEVFGKEkVNELSK | K13(Lac) | 4 | 623.073 | 46.72 | 4.6 | 63.4 | | | x |
| 21 | *24* | *42* | *K36* | FVAPFPEVFGKEkVNELSK | K13(Hex) | 4 | 582.561 | 47.19 | 5.6 | 53.5 | | | x |
| 22 | *25* | *36* | *K34* | VAPFPEVFGkEK | K10(Lac) | 3 | 557.952 | 38.25 | 1.6 | 32.2 | | x | x |
| 23 | *25* | *36* | *K34* | VAPFPEVFGkEK | K10(Hex) | 3 | 503.932 | 39.07 | 1.8 | 9.0 | | | x |
| 24 | *25* | *37* | *K34, K36* | VAPFPEVFGkEkV | K10(Hex); K12(Lac) | 3 | 644.993 | 42.19 | 2.8 | | | | x |
| 25 | *25* | *40* | *K34* | VAPFPEVFGkEKVNEL | K10(Lac) | 3 | 709.699 | 47.30 | 2.7 | | | | x |

| Nr. | *Start pos.* | *End pos.* | *Mod. Site* | Annotated Sequence | Modifications | z | *m/z* | $t_R$ (min) | XCorr | -10lgP | RM | UHT | IF |
|---|---|---|---|---|---|---|---|---|---|---|---|---|---|
| 26 | *25* | *42* | *K34* | VAPFPEVFGkEKVNELSK | K10(Lac) | 4 | 586.308 | 40.86 | 4.1 | 49.4 | | | x |
| 27 | *25* | *42* | *K36* | VAPFPEVFGKEkVNELSK | K12(Lac) | 4 | 586.307 | 41.01 | 3.5 | 63.0 | | | x |
| 28 | *26* | *36* | *K34* | APFPEVFGkEK | K9(Lac) | 3 | 524.926 | 35.37 | 2.0 | 26.2 | | x | x |
| 29 | *26* | *37* | *K34* | APFPEVFGkEKV | K9(Hex) | 3 | 503.933 | 40.21 | 2.4 | 29.6 | | | x |
| 30 | *26* | *37* | *K34, K36* | APFPEVFGkEkV | K9(Lac); K11(Lac) | 3 | 665.987 | 39.25 | 2.3 | 33.0 | | | x |
| 31 | *28* | *37* | *K34* | FPEVFGkEKV | K7(Lac) | 3 | 501.920 | 35.84 | 2.5 | 27.1 | | | x |
| 32 | *80* | *90* | *K83* | HIQkEDVPSER | K4(Lac) | 3 | 554.600 | 13.76 | 2.8 | 12.5 | x | x | x |
| 33 | *80* | *91* | *K83* | HIQkEDVPSERY | K4(Lac) | 3 | 608.955 | 18.73 | 2.7 | 29.5 | | x | x |
| 34 | *80* | *92* | *K83* | HIQkEDVPSERYL | K4(Lac) | 3 | 646.650 | 26.26 | 3.0 | 30.7 | | | x |
| 35 | *80* | *93* | *K83* | HIQkEDVPSERYLG | K4(Lac) | 3 | 665.658 | 24.37 | 3.5 | 32.8 | | x | x |
| 36 | *80* | *94* | *K83* | HIQkEDVPSERYLGY | K4(Lac) | 3 | 720.013 | 29.80 | 2.6 | 23.3 | | | x |
| 37 | *80* | *96* | *K83* | HIQkEDVPSERYLGYLE | K4(Lac) | 3 | 800.722 | 37.83 | 1.9 | 10.9 | | | x |
| 38 | *80* | *99* | *K83* | HIQkEDVPSERYLGYLEQLL | K4(Lac) | 4 | 689.349 | 60.43 | 2.5 | 17.4 | | x | x |
| 39 | *80* | *100* | *K83* | HIQkEDVPSERYLGYLEQLLR | K4(Lac) | 4 | 728.376 | 57.22 | 5.1 | 51.1 | | x | x |
| 40 | *80* | *100* | *K83* | HIQkEDVPSERYLGYLEQLLR | K4(Hex) | 4 | 687.862 | 57.64 | 5.2 | 51.7 | | | x |
| 41 | *80* | *101* | *K83* | HIQkEDVPSERYLGYLEQLLRL | K4(Lac) | 4 | 756.649 | 66.16 | 5.1 | 36.8 | | x | x |
| 42 | *80* | *102* | *K102* | HIQKEDVPSERYLGYLEQLLRLk | K23(Lac) | 5 (4) | 631.136 | 59.33 | 6.7 | 42.8 | x | x | x |
| 43 | *80* | *102* | *K83* | HIQkEDVPSERYLGYLEQLLRLK | K4(Lac) | 5 | 631.138 | 60.64 | 5.0 | 26.9 | x | x | x |
| 44 | *80* | *102* | *K83* | HIQkEDVPSERYLGYLEQLLRLK | K4(Hex) | 5 | 598.726 | 60.17 | 6.5 | 36.4 | | | x |
| 45 | *80* | *102* | *K83, K102* | HIQkEDVPSERYLGYLEQLLRLk | K4(Lac); K23(Lac) | 5 | 695.959 | 58.72 | 5.8 | | | | x |
| 46 | *80* | *103* | *K102* | HIQKEDVPSERYLGYLEQLLRLkK | K23(Lac) | 5 | 656.758 | 55.15 | 4.7 | 29.5 | | x | x |
| 47 | *80* | *104* | *K102* | HIQKEDVPSERYLGYLEQLLRLkKY | K23(Lac) | 5 | 689.369 | 55.84 | 4.5 | 25.9 | | x | x |
| 48 | *80* | *104* | *K103* | HIQKEDVPSERYLGYLEQLLRLKkY | K24(Lac) | 5 | 689.370 | 55.89 | 4.8 | 30.6 | | | x |
| 49 | *81* | *90* | *K83* | IQkEDVPSER | K3(Lac) | 3 | 508.914 | 14.69 | 2.6 | 15.1 | | x | x |
| 50 | *81* | *90* | *K83* | IQkEDVPSER | K3(Hex) | 3 | 454.896 | 15.28 | 2.0 | | | | x |
| 51 | *81* | *91* | *K83* | QkEDVPSERY | K2(Lac) | 3 | 525.573 | 17.67 | 1.6 | 12.2 | | x | x |
| 52* | *103* | *123* | *K103* | kYKVPQLEIVPNsAEERLHSM | K1(Lac); S13(Phospho) | 4 | 718.849 | 44.17 | 4.0 | | x | x | x |
| 53* | *103* | *123* | *K103* | kYKVPQLEIVPNsAEERLHSm | K1(Lac); S13(Phospho); M21(Ox) | 4 | 722.849 | 39.10 | 3.5 | | x | x | x |
| 54* | *103* | *123* | *K103, K105* | kYkVPQLEIVPNsAEERLHSM | K1(Hex); K3(Hex); S13(Phospho) | 4 | 718.850 | 44.18 | 3.9 | | x | x | x |

| Nr. | *Start pos.* | *End pos.* | *Mod. Site* | Annotated Sequence | Modifications | z | *m/z* | $t_R$ (min) | XCorr | -10lgP | RM | UHT | IF |
|---|---|---|---|---|---|---|---|---|---|---|---|---|---|
| 55* | *103* | *123* | *K103, K105* | kYkVPQLEIVPNsAEERLHSm | K1(Hex); K3(Hex); S13(Phospho); M21(Ox) | 4 | 722.849 | 38.99 | 3.7 | | | x | x |
| 56* | *103* | *123* | *K103, K105* | kYkVPQLEIVPNsAEERLHSM | K1(Lac); K3(Lac); S13(Phospho) | 4 | 799.878 | 43.93 | 2.8 | | | | x |
| 57* | *103* | *123* | *K105* | KYkVPQLEIVPNsAEERLHSm | K3(Lac); S13(Phospho); M21(Ox) | 4 | 722.849 | 39.06 | 3.5 | 45.4 | | x | x |
| 58* | *103* | *123* | *K105* | KYkVPQLEIVPNsAEERLHSM | K3(Hex); S13(Phospho) | 4 | 678.337 | 44.31 | 4.2 | 39.3 | | | x |
| *59** | *103* | *124* | *K103, K105* | kYkVPQLEIVPNsAEERLHSMK | K1(Hex); K3(Hex); S13(Phospho) | 4 | 750.873 | 40.28 | 3.0 | | | x | x |
| *60** | *103* | *124* | *K105* | KYkVPQLEIVPNsAEERLHSMK | K3(Lac); S13(Phospho) | 4 | 750.872 | 40.47 | 2.4 | | | x | x |
| 61 | *104* | *123* | *K105* | YkVPQLEIVPNSAEERLHSM | K2(Lac) | 4 | 666.834 | 45.56 | 3.3 | 48.8 | | x | x |
| 62* | *104* | *123* | *K105* | YkVPQLEIVPNsAEERLHSM | K2(Lac); S12(Phospho) | 4 | 686.826 | 47.74 | 3.2 | 36.2 | | | x |
| 63* | *105* | *123* | *K105* | kVPQLEIVPNsAEERLHSM | K1(Lac); S11(Phospho) | 4 | 646.060 | 44.37 | 4.4 | 39.7 | | | x |
| 64* | *105* | *124* | *K105* | kVPQLEIVPNsAEERLHSMK | K1(Lac); S11(Phospho) | 4 | 678.084 | 39.97 | 2.9 | | | | x |
| **Alpha-S2-casein (P02663)** | | | | | | | | | | | | | |
| *65* | *1* | *24* | *K21* | KNTMEHVSSSEESIISQETYkQEK | K21(Lac) | 4 | 784.863 | 29.54 | 4.4 | 44.2 | | x | x |
| 66* | *1* | *24* | *K21* | KNTMEHVSSSEESIIsQETYkQEK | S16(Phospho); K21(Lac) | 4 | 804.860 | 30.25 | 3.4 | 35.8 | | x | x |
| *67* | *1* | *24* | *K24* | KNTMEHVSSSEESIISQETYKQEk | K24(Lac) | 4 | 784.867 | 29.46 | 5.3 | 40.8 | | x | x |
| *68* | *1* | *24* | *K24* | KNTmEHVSSSEESIISQETYKQEk | M4(Ox); K24(Lac) | 4 | 788.865 | 26.08 | 3.2 | | | x | x |
| 69* | *1* | *24* | *K24* | KNTmEHVSSSEESIIsQETYKQEk | M4(Ox); S16(Phospho); K24(Lac) | 4 | 808.858 | 26.96 | 3.2 | | | x | x |
| 70* | *1* | *24* | *K24* | KNTMEHVSSSEEsIISQETYKQEk | S13(Phospho); K24(Lac) | 4 | 804.861 | 30.38 | 3.2 | 19.6 | | x | x |
| 71* | *137* | *150* | *K150* | KTVDMEsTEVFTKk | S7(Phospho); K14(Lac) | 3 | 682.976 | 26.97 | 4.2 | 44.0 | x | x | x |
| 72* | *138* | *150* | *K150* | TVDMEsTEVFTKk | S6(Phospho); K13(Lac) | 3 | 640.278 | 30.53 | 2.0 | 21.6 | | x | x |
| 73 | *138* | *150* | *K150* | TVDMESTEVFTKk | K13(Lac) | 3 | 613.620 | 30.00 | 2.2 | 21.3 | | x | x |
| 74 | *150* | *162* | *K150, K152* | kTkLTEEEKNRLN | K1(Lac); K3(Lac) | 4 | 563.528 | 14.36 | 2.9 | 22.2 | | | x |
| 75 | *150* | *163* | *K150* | kTKLTEEEKNRLNF | K1(Lac) | 4 | 519.269 | 24.23 | 5.5 | 57.5 | | | x |
| 76 | *150* | *163* | *K150, K152* | kTkLTEEEKNRLNF | K1(Lac); K3(Lac) | 4 | 600.297 | 23.94 | 3.2 | | | | x |
| 77 | *150* | *163* | *K152* | KTkLTEEEKNRLNF | K3(Hex) | 4 | 478.756 | 24.41 | 3.9 | | | | x |
| 78 | *150* | *163* | *K152* | KTkLTEEEKNRLNF | K3(Lac) | 4 | 519.272 | 24.18 | 4.9 | | | | x |
| 79 | *150* | *163* | *K152, K158* | KTkLTEEEkNRLNF | K3(Lac); K9(Lac) | 4 | 600.296 | 24.25 | 3.4 | | | | x |
| 80 | *150* | *163* | *K158* | KTKLTEEEkNRLNF | K9(Lac) | 3 | 692.026 | 24.31 | 3.0 | | | | x |
| 81 | *150* | *164* | *K150* | kTKLTEEEKNRLNFL | K1(Lac) | 3 | 729.720 | 31.77 | 2.1 | 9.4 | | x | x |
| 82 | *150* | *164* | *K150, K152* | kTkLTEEEKNRLNFL | K1(Lac); K3(Lac) | 4 | 628.569 | 31.48 | 3.4 | | | | x |

| Nr. | *Start pos.* | *End pos.* | *Mod. Site* | Annotated Sequence | Modifications | z | *m/z* | $t_R$ (min) | XCorr | -10lgP | RM | UHT | IF |
|---|---|---|---|---|---|---|---|---|---|---|---|---|---|
| 83 | *150* | *164* | *K152* | KTkLTEEEKNRLNFL | K3(Hex) | 4 | 507.027 | 32.14 | 4.1 | 43.7 | | | x |
| 84 | *150* | *164* | *K152* | KTkLTEEEKNRLNFL | K3(Lac) | 4 | 547.542 | 31.69 | 4.1 | 48.6 | x | x | x |
| 85 | *150* | *164* | *K158* | KTKLTEEEkNRLNFL | K9(Lac) | 4 | 547.540 | 31.96 | 4.8 | 36.4 | x | x | x |
| 86 | *150* | *165* | *K150* | kTKLTEEEKNRLNFLK | K1(Lac) | 4 (5) | 579.566 | 27.33 | 5.6 | 42.7 | x | x | x |
| 87 | *150* | *165* | *K150, K152* | kTkLTEEEKNRLNFLK | K1(Lac); K3(Lac) | 5 | 528.674 | 27.07 | 5.2 | | | | x |
| 88 | *150* | *165* | *K152* | KTkLTEEEKNRLNFLK | K3(Lac) | 5 | 463.852 | 27.56 | 5.9 | 34.9 | | x | x |
| 89 | *150* | *165* | *K152, K158* | KTkLTEEEkNRLNFLK | K3(Lac); K9(Lac) | 5 (4) | 528.674 | 27.19 | 5.1 | 43.5 | | | x |
| 90 | *150* | *165* | *K158* | KTKLTEEEkNRLNFLK | K9(Lac) | 5 | 463.853 | 27.36 | 5.6 | 31.8 | x | x | x |
| 91 | *150* | *165* | *K165* | KTKLTEEEKNRLNFLk | K16(Lac) | 4 | 579.565 | 27.38 | 4.7 | 42.7 | x | x | x |
| 92 | *151* | *161* | *K152* | TkLTEEEKNRL | K2(Hex) | 3 | 508.269 | 19.07 | 2.5 | 18.1 | | | x |
| 93 | *151* | *161* | *K152* | TkLTEEEKNRL | K2(Lac) | 3 | 562.287 | 18.95 | 2.5 | 31.7 | | | x |
| 94 | *151* | *161* | *K152, K158* | TkLTEEEkNRL | K2(Lac); K8(Lac) | 3 | 670.324 | 18.65 | 3.2 | 31.7 | | | x |
| 95 | *151* | *161* | *K158* | TKLTEEEkNRL | K8(Lac) | 3 | 562.288 | 18.63 | 3.1 | 32.6 | | | x |
| 96 | *151* | *162* | *K152, K158* | TkLTEEEkNRLN | K2(Lac); K8(Lac) | 4 | 531.505 | 16.72 | 4.1 | 32.8 | | | x |
| 97 | *151* | *162* | *K158* | TKLTEEEkNRLN | K8(Lac) | 4 (3) | 450.478 | 16.55 | 4.6 | 40.7 | | x | x |
| 98 | *151* | *162* | *K158* | TKLTEEEkNRLN | K8(Hex) | 3 | 546.284 | 17.00 | 2.8 | 17.9 | | | x |
| 99 | *151* | *163* | *K152, K158* | TkLTEEEkNRLNF | K2(Lac); K8(Lac) | 3 | 757.362 | 27.99 | 2.6 | 28.0 | | | x |
| 100 | *151* | *163* | *K158* | TKLTEEEkNRLNF | K8(Lac) | 3 | 649.325 | 28.38 | 3.7 | 26.2 | | | x |
| 101 | *151* | *164* | *K152* | TkLTEEEKNRLNFL | K2(Lac) | 3 | 687.022 | 35.78 | 3.3 | 35.9 | | x | x |
| 102 | *151* | *164* | *K158* | TKLTEEEkNRLNFL | K8(Lac) | 4 (3) | 515.517 | 36.11 | 5.0 | 33.6 | | x | x |
| 103 | *151* | *164* | *K158* | TKLTEEEkNRLNFL | K8(Hex) | 3 | 633.003 | 36.19 | 3.3 | | | | x |
| 104 | *151* | *165* | *K152* | TkLTEEEKNRLNFLK | K2(Lac) | 3 | 729.719 | 31.10 | 2.5 | 29.4 | | x | x |
| 105 | *151* | *165* | *K158* | TKLTEEEkNRLNFLK | K8(Lac) | 4 | 547.541 | 31.09 | 5.0 | 59.9 | x | x | x |
| 106 | *151* | *165* | *K158* | TKLTEEEkNRLNFLK | K8(Hex) | 4 | 507.027 | 31.13 | 3.4 | | | x | x |
| 107 | *151* | *165* | *K158, K165* | TKLTEEEkNRLNFLk | K8(Lac); K15(Lac) | 4 | 628.568 | 30.73 | 4.3 | 34.5 | | | x |
| 108 | *151* | *165* | *K165* | TKLTEEEKNRLNFLk | K15(Lac) | 4 | 547.540 | 30.68 | 4.6 | 40.9 | x | x | x |
| 109 | *151* | *169* | *K152* | TkLTEEEKNRLNFLKKISQ | K2(Lac) | 4 | 661.610 | 35.50 | 4.0 | | | x | x |
| 110 | *152* | *163* | *K158* | KLTEEEkNRLNF | K7(Lac) | 3 | 615.643 | 26.00 | 2.9 | | | | x |
| 111 | *153* | *162* | *K158* | LTEEEkNRLN | K6(Lac) | 3 | 523.922 | 14.31 | 2.6 | 13.4 | | | x |
| 112 | *153* | *163* | *K158* | LTEEEkNRLNF | K6(Lac) | 3 | 572.944 | 28.00 | 3.0 | 28.4 | | | x |

| Nr. | *Start pos.* | *End pos.* | *Mod. Site* | Annotated Sequence | Modifications | z | *m/z* | $t_R$ (min) | XCorr | -10lgP | RM | UHT | IF |
|---|---|---|---|---|---|---|---|---|---|---|---|---|---|
| 113 | *153* | *165* | *K158* | LTEEEkNRLNFLK | K6(Lac) | 3 | 653.336 | 31.23 | 1.9 | 9.8 | | | x |
| 114 | *153* | *165* | *K158, K165* | LTEEEkNRLNFLk | K6(Lac); K13(Lac) | 4 | 571.282 | 30.55 | 4.3 | | | | x |
| 115 | *154* | *163* | *K158* | TEEEkNRLNF | K5(Lac) | 3 | 535.251 | 23.49 | 2.6 | | | | x |
| 116 | *154* | *164* | *K158* | TEEEkNRLNFL | K5(Lac) | 3 | 572.945 | 34.52 | 2.6 | 25.8 | | x | x |
| 117 | *154* | *165* | *K158* | TEEEkNRLNFLK | K5(Lac) | 4 | 461.984 | 26.98 | 4.5 | 34.3 | | | x |
| 118 | *155* | *163* | *K158* | EEEkNRLNF | K4(Lac) | 3 | 501.567 | 22.91 | 2.6 | 20.3 | | | x |
| 119 | *155* | *164* | *K158* | EEEkNRLNFL | K4(Lac) | 3 | 539.262 | 34.46 | 2.9 | 31.3 | | x | x |
| 120 | *170* | *179* | *K173* | RYQkFALPQY | K4(Lac) | 3 | 546.607 | 34.97 | 2.2 | 10.3 | | | x |
| 121 | *185* | *197* | *K188* | QHQkAMkPWIQPK | K4(Lac); K7(Lac) | 4 | 567.781 | 19.03 | 3.2 | 18.8 | | | x |
| 122* | *22* | *41* | *T38* | QEKNmAINPsKENLCStFCK | M5(Ox); S10(Phospho); T17(TOx) | 4 | 595.511 | 34.35 | 4.1 | | x | x | |
| 123 | *22* | *41* | *T38* | QEKNmAINPSKENLCStFCK | M5(Ox); T17(TOx) | 4 | 575.519 | 32.24 | 4.9 | | x | x | |
| 124* | *22* | *41* | *T38* | QEKNMAINPsKENLCStFCK | S10(Phospho); T17(TOx) | 4 | 591.513 | 37.73 | 4.8 | 8.6 | x | x | |
| 125 | *22* | *41* | *T38* | QEKNMAINPSKENLCStFCK | T17(TOx) | 4 | 571.520 | 35.47 | 5.2 | 51.1 | x | x | |
| 126* | *22* | *45* | *T38* | QEKNmAINPsKENLCStFCKEVVR | M5(Ox); S10(Phospho); T17(TOx) | 4 | 716.332 | 40.23 | 4.9 | | x | x | |
| 127 | *22* | *45* | *T38* | QEKNmAINPSKENLCStFCKEVVR | M5(Ox); T17(TOx) | 5 | 557.273 | 37.97 | 4.9 | | x | x | x |
| 128* | *22* | *45* | *T38* | QEKNMAINPsKENLCStFCKEVVR | S10(Phospho); T17(TOx) | 4 | 712.334 | 42.84 | 4.7 | 13.8 | x | x | |
| 129 | *22* | *45* | *T38* | QEKNMAINPSKENLCStFCKEVVR | T17(TOx) | 4 (5) | 692.341 | 40.20 | 4.7 | 45.1 | x | x | x |
| 130* | *25* | *41* | *T38* | NmAINPsKENLCStFCK | M2(Ox); S7(Phospho); T14(TOx) | 3 | 665.283 | 37.32 | 3.0 | | x | x | x |
| 131 | *25* | *41* | *T38* | NmAINPSKENLCStFCK | M2(Ox); T14(TOx) | 3 | 638.625 | 34.94 | 3.5 | | x | x | x |
| 132* | *25* | *41* | *T38* | NMAINPsKENLCStFCK | S7(Phospho); T14(TOx) | 3 | 659.949 | 41.81 | 2.5 | | x | x | x |
| 133 | *25* | *41* | *T38* | NMAINPSKENLCStFCK | T14(TOx) | 3 | 633.294 | 39.11 | 2.9 | 24.6 | x | x | x |
| 134* | *25* | *45* | *T38* | NmAINPsKENLCStFCKEVVR | M2(Ox); S7(Phospho); T14(TOx) | 4 | 620.032 | 43.07 | 4.8 | | x | x | x |
| 135 | *25* | *45* | *T38* | NmAINPSKENLCStFCKEVVR | M2(Ox); T14(TOx) | 4 | 600.041 | 40.14 | 4.3 | | x | x | x |
| 136* | *25* | *45* | *T38* | NMAINPsKENLCStFCKEVVR | S7(Phospho); T14(TOx) | 4 | 616.034 | 46.37 | 4.4 | | x | x | x |
| 137 | *25* | *45* | *T38* | NMAINPSKENLCStFCKEVVR | T14(TOx) | 4 | 596.043 | 43.35 | 4.8 | 45.1 | x | x | x |
| **Beta-casein (P02666)** | | | | | | | | | | | | | |
| 138* | *29* | *40* | *K29* | kIEKFQsEEQQQ | K1(Lac); S7(Phospho) | 3 | 642.614 | 17.96 | 2.1 | 11.4 | | | x |
| 139 | *29* | *48* | *K29* | kIEKFQSEEQQQTEDELQDK | K1(Lac) | 4 | 701.828 | 25.70 | 3.2 | 34.3 | | x | x |
| 140* | *29* | *48* | *K29* | kIEKFQsEEQQQTEDELQDK | K1(Lac); S7(Phospho) | 4 | 721.820 | 28.68 | 4.5 | | | x | x |
| 141 | *29* | *48* | *K32* | KIEkFQSEEQQQTEDELQDK | K4(Lac) | 4 | 701.829 | 26.21 | 4.5 | 52.7 | x | x | x |

| Nr. | *Start pos.* | *End pos.* | *Mod. Site* | Annotated Sequence | Modifications | z | *m/z* | $t_R$ (min) | XCorr | -10lgP | RM | UHT | IF |
|---|---|---|---|---|---|---|---|---|---|---|---|---|---|
| 142* | *29* | *48* | *K32* | KIEkFQsEEQQQTEDELQDK | K4(Lac); S7(Phospho) | 4 | 721.820 | 28.48 | 4.4 | 42.7 | x | x | x |
| **Butyrophilin subfamily 1 member A1 (P18892)** | | | | | | | | | | | | | |
| 143 | *153* | *177* | *P160* | THRGEEFpSMSESRNPDEEGLFTVR | P8(Ox) | 5 | 585.472 | 34.45 | 5.0 | 32.8 | x | x | x |
| **Fibroblast growth factor-binding protein 1 (Q9MZ06)** | | | | | | | | | | | | | |
| 144 | *6* | *27* | *K22* | NRRGSKASADESLALGkPGKEPR | K17(Lac) | 5 (6) | 550.488 | 17.22 | 3.8 | 13.2 | | x | x |
| 145 | *8* | *27* | *K25* | RGSKASADESLALGKPGkEPR | K18(Lac) | 5 | 496.459 | 18.43 | 5.8 | 24.0 | | x | x |
| 146 | *9* | *27* | *K22* | GSKASADESLALGkPGKEPR | K14(Lac) | 5 (4) | 465.239 | 20.15 | 4.7 | 39.3 | | x | x |
| 147 | *9* | *27* | *K25* | GSKASADESLALGKPGkEPR | K17(Lac) | 4 | 581.298 | 20.10 | 3.6 | | | x | x |
| **Glycosylation-dependent cell adhesion molecule 1 (P80195)** | | | | | | | | | | | | | |
| 148 | *1* | *10* | *K4* | ILNkPEDETH | K4(Lac) | 3 | 507.238 | 14.06 | 1.9 | 9.2 | | | x |
| 149 | *1* | *11* | *K4* | ILNkPEDETHL | K4(Lac) | 3 | 544.933 | 24.44 | 2.3 | 34.2 | | | x |
| 150 | *1* | *12* | *K4* | ILNkPEDETHLE | K4(Lac) | 3 | 587.948 | 23.42 | 3.0 | 32.3 | | | x |
| 151 | *1* | *16* | *K4* | ILNkPEDETHLEAQPT | K4(Lac) | 3 | 720.350 | 25.40 | 2.7 | 27.0 | | | x |
| 152 | *1* | *24* | *K4* | ILNkPEDETHLEAQPTDASAQFIR | K4(Lac) | 4 | 762.630 | 35.75 | 5.4 | 59.0 | x | x | x |
| 153 | *1* | *26* | *K4* | ILNkPEDETHLEAQPTDASAQFIRNL | K4(Lac) | 4 | 819.408 | 46.22 | 3.9 | 33.3 | | | x |
| 154 | *2* | *13* | *K4* | LNkPEDETHLE | K3(Lac) | 3 | 550.253 | 19.98 | 2.4 | 30.5 | | | x |
| 155 | *54* | *67* | *K64* | SSRQPQSQNPkLPL | K11(Hex) | 3 | 581.308 | 27.54 | 2.0 | 16.1 | | | x |
| 156 | *54* | *70* | *K64* | SSRQPQSQNPkLPLSIL | K11(Hex) | 3 | 685.710 | 44.63 | 3.8 | 10.1 | | | x |
| 157 | *54* | *71* | *K71* | SSRQPQSQNPKLPLSILk | K18(Lac) | 4 | 587.071 | 36.31 | 4.5 | 41.3 | | | x |
| 158 | *54* | *73* | *K71* | SSRQPQSQNPKLPLSILkEK | K18(Lac) | 5 (4) | 521.285 | 33.40 | 3.7 | 10.0 | | x | x |
| 159 | *54* | *73* | *K71, K73* | SSRQPQSQNPKLPLSILkEk | K18(Lac); K20(Lac) | 5 | 586.107 | 32.73 | 4.8 | | | | x |
| 160 | *54* | *73* | *K73* | SSRQPQSQNPKLPLSILKEk | K20(Lac) | 5 (4) | 521.285 | 33.30 | 4.9 | 31.7 | | x | x |
| 161 | *54* | *75* | *K64, K73* | SSRQPQSQNPkLPLSILkEKHL | K11(Lac); K18(Lac) | 5 | 636.134 | 35.60 | 3.1 | | | | x |
| 162 | *54* | *75* | *K71* | SSRQPQSQNPKLPLSILkEKHL | K18(Lac) | 5 | 571.314 | 35.94 | 6.1 | 47.2 | | | x |
| 163 | *54* | *75* | *K73* | SSRQPQSQNPKLPLSILKEkHL | K20(Lac) | 5 (4) | 571.315 | 35.40 | 6.4 | 37.2 | | | x |
| 164 | *109* | *121* | *K116, K120* | RNLENTVkETIkY | K8(Lac); K12(Lac) | 3 | 752.702 | 32.21 | 3.0 | 22.6 | | | x |
| 165 | *109* | *121* | *K120* | RNLENTVKETIkY | K12(Lac) | 3 | 644.665 | 32.31 | 3.8 | 42.7 | | | x |
| 166 | *111* | *121* | *K116, K120* | LENTVkETIkY | K6(Lac); K10(Lac) | 3 | 662.653 | 26.84 | 2.0 | 17.9 | | | x |
| 167 | *111* | *121* | *K120* | LENTVKETIkY | K10(Lac) | 3 | 554.618 | 27.24 | 2.8 | | | | x |
| 168 | *112* | *121* | *K116* | ENTVkETIKY | K5(Lac) | 3 | 516.923 | 22.96 | 3.1 | 35.0 | | | x |

| Nr. | *Start pos.* | *End pos.* | *Mod. Site* | Annotated Sequence | Modifications | z | *m/z* | $t_R$ (min) | XCorr | -10lgP | RM | UHT | IF |
|---|---|---|---|---|---|---|---|---|---|---|---|---|---|
| 169 | *112* | *121* | *K116, K120* | ENTVkETIkY | K5(Lac); K9(Lac) | 3 | 624.958 | 22.73 | 2.1 | 24.9 | | | x |
| 170 | *123* | *135* | *K134* | KSLFSHAFEVVkT | K12(Lac) | 3 | 606.313 | 38.11 | 2.6 | | | | x |
| 171 | *126* | *135* | *K134* | FSHAFEVVkT | K9(Lac) | 3 | 496.909 | 29.14 | 2.8 | 41.3 | | | x |
| 172 | *126* | *135* | *K134* | FSHAFEVVkT | K9(Hex) | 3 | 442.891 | 29.73 | 2.4 | 43.6 | | | x |
| 173 | *127* | *135* | *K134* | SHAFEVVkT | K8(Lac) | 3 | 447.886 | 21.75 | 2.1 | | | | x |
| **Lactoperoxidase (P80025)** | | | | | | | | | | | | | |
| 174 | *68* | *76* | *K74* | VWEESLkRL | K7(Lac) | 3 | 495.255 | 34.81 | 2.1 | 27.2 | | x | x |
| 175 | *69* | *76* | *K74* | WEESLkRL | K6(Lac) | 3 | 462.233 | 30.59 | 2.1 | 13.1 | | | x |

*Phosphorylation sites rely on software tools without further confirmation of their location.

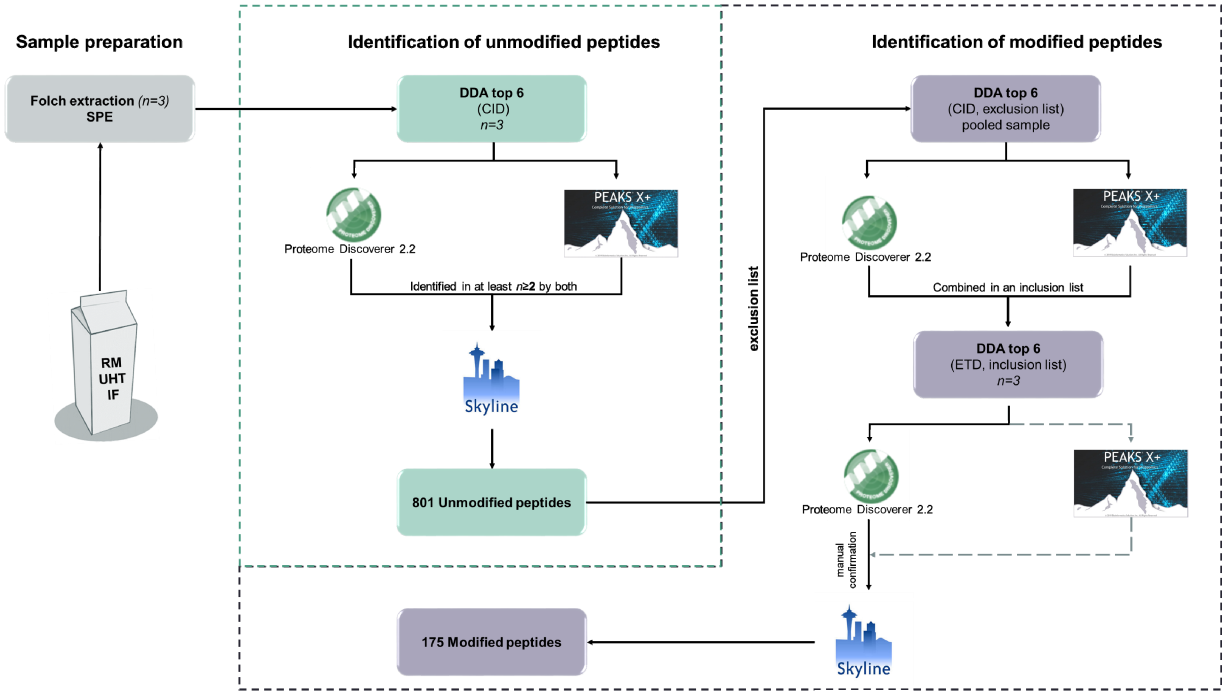


**Figure S1:** Strategy applied for the identification of unmodified and modified endogenous peptides in RM, UHT milk, and IF relying on software tools Proteome Discoverer 2.2 and PEAKS 10.5 in combination with a spectra library generated within Skyline.

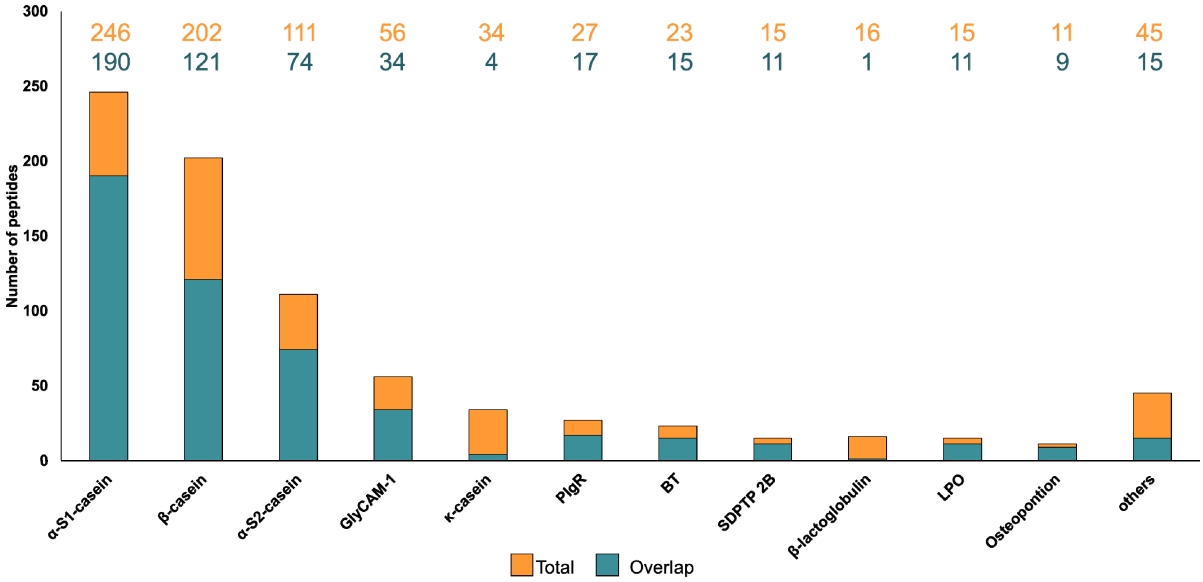


**Figure S2:** Total number of peptides identified for different proteins (orange) and the number of overlapping peptides (blue) present in RM, UHT milk, and IF. Abbreviations: GlyCAM-1 - glycosylation-dependent cell adhesion molecule 1, PigR - polymeric immunoglobulin receptor, BT - butyrophilin subfamily 1 member A1, SDPTP 2B - sodium-dependent phosphate transport protein 2B, and LPO – lactoperoxidase.

| Position | 1 | 2 | 3 | 4 | 5 | 6 | 7 | 8 | 9 | 10 | 11 | 12 | 13 | 14 | 15 | 16 | 17 | 18 | 19 | 20 | 21 | 22 | 23 | 24 | 25 | 26 | 27 | 28 | 29 | 30 | Position |
|---|---|---|---|---|---|---|---|---|---|---|---|---|---|---|---|---|---|---|---|---|---|---|---|---|---|---|---|---|---|---|---|
| AA | R | P | K | H | P | I | K | H | Q | G | L | P | Q | E | V | L | N | E | N | L | L | R | F | F | V | A | P | F | P | E | AA |
| **Total** | **2** | **2** | **2** | **6** | **6** | **6** | **8** | **10** | **14** | **21** | **25** | **25** | **27** | **31** | **33** | **36** | **37** | **35** | **34** | **33** | **31** | **24** | **22** | **18** | **22** | **30** | **33** | **36** | **36** | **36** | **Total** |
| RM | 2 | 2 | 2 | 6 | 6 | 6 | 7 | 9 | 10 | 15 | 19 | 19 | 20 | 22 | 24 | 27 | 28 | 27 | 27 | 26 | 24 | 19 | 17 | 17 | 22 | 30 | 33 | 36 | 36 | 36 | RM |
| UHT | 2 | 2 | 2 | 6 | 6 | 6 | 7 | 9 | 10 | 15 | 19 | 19 | 20 | 22 | 24 | 27 | 28 | 27 | 27 | 26 | 24 | 19 | 17 | 17 | 22 | 30 | 33 | 36 | 36 | 36 | UHT |
| IF | 2 | 2 | 2 | 5 | 5 | 5 | 7 | 9 | 13 | 20 | 23 | 23 | 25 | 29 | 31 | 33 | 34 | 32 | 31 | 30 | 29 | 22 | 19 | 16 | 20 | 27 | 30 | 33 | 33 | 33 | IF |

| Position | 31 | 32 | 33 | 34 | 35 | 36 | 37 | 38 | 39 | 40 | 41 | 42 | 43 | 44 | 45 | 46 | 47 | 48 | 49 | 50 | 51 | 52 | 53 | 54 | 55 | 56 | 57 | 58 | 59 | 60 | Position |
|---|---|---|---|---|---|---|---|---|---|---|---|---|---|---|---|---|---|---|---|---|---|---|---|---|---|---|---|---|---|---|---|
| AA | V | F | G | K | E | K | V | N | E | L | S | K | D | I | G | S | E | S | T | E | D | Q | A | M | E | D | I | K | Q | M | AA |
| **Total** | **36** | **36** | **34** | **30** | **27** | **23** | **20** | **15** | **15** | **13** | **12** | **8** | **10** | **11** | **11** | **11** | **11** | **11** | **11** | **11** | **11** | **11** | **11** | **10** | **10** | **10** | **10** | **10** | **0** | **0** | **Total** |
| RM | 36 | 36 | 34 | 30 | 27 | 23 | 20 | 15 | 15 | 13 | 12 | 8 | 10 | 11 | 11 | 11 | 11 | 11 | 11 | 11 | 11 | 11 | 11 | 10 | 10 | 10 | 10 | 10 | | | RM |
| UHT | 36 | 36 | 34 | 30 | 27 | 23 | 20 | 15 | 15 | 13 | 12 | 8 | 10 | 11 | 11 | 11 | 11 | 11 | 11 | 11 | 11 | 11 | 11 | 10 | 10 | 10 | 10 | 10 | | | UHT |
| IF | 33 | 33 | 31 | 29 | 26 | 23 | 20 | 15 | 15 | 13 | 12 | 8 | 9 | 9 | 9 | 9 | 9 | 9 | 9 | 9 | 9 | 9 | 9 | 8 | 8 | 8 | 8 | 8 | | | IF |

| Position | 61 | 62 | 63 | 64 | 65 | 66 | 67 | 68 | 69 | 70 | 71 | 72 | 73 | 74 | 75 | 76 | 77 | 78 | 79 | 80 | 81 | 82 | 83 | 84 | 85 | 86 | 87 | 88 | 89 | 90 | Position |
|---|---|---|---|---|---|---|---|---|---|---|---|---|---|---|---|---|---|---|---|---|---|---|---|---|---|---|---|---|---|---|---|
| AA | E | A | E | S | I | S | S | S | E | E | I | V | P | N | S | V | E | Q | K | H | I | Q | K | E | D | V | P | S | E | R | AA |
| **Total** | **0** | **0** | **0** | **0** | **0** | **0** | **0** | **0** | **1** | **1** | **1** | **1** | **1** | **1** | **1** | **1** | **1** | **1** | **1** | **15** | **20** | **21** | **23** | **24** | **24** | **27** | **27** | **27** | **27** | **26** | **Total** |
| RM | | | | | | | | | | | | | | | | | | | | 15 | 20 | 21 | 23 | 24 | 24 | 27 | 27 | 27 | 27 | 26 | RM |
| UHT | | | | | | | | | | | | | | | | | | | | 13 | 18 | 19 | 21 | 22 | 22 | 25 | 25 | 25 | 25 | 24 | UHT |
| IF | | | | | | | | | 1 | 1 | 1 | 1 | 1 | 1 | 1 | 1 | 1 | 1 | 1 | 15 | 20 | 20 | 21 | 22 | 22 | 24 | 24 | 24 | 24 | 23 | IF |

| Position | 91 | 92 | 93 | 94 | 95 | 96 | 97 | 98 | 99 | 100 | 101 | 102 | 103 | 104 | 105 | 106 | 107 | 108 | 109 | 110 | 111 | 112 | 113 | 114 | 115 | 116 | 117 | 118 | 119 | 120 | Position |
|---|---|---|---|---|---|---|---|---|---|---|---|---|---|---|---|---|---|---|---|---|---|---|---|---|---|---|---|---|---|---|---|
| AA | Y | L | G | Y | L | E | Q | L | L | R | L | K | K | Y | K | V | P | Q | L | E | I | V | P | N | S | A | E | E | R | L | AA |
| **Total** | **28** | **26** | **28** | **27** | **28** | **27** | **26** | **24** | **22** | **18** | **14** | **10** | **12** | **18** | **25** | **37** | **37** | **42** | **45** | **47** | **48** | **49** | **49** | **50** | **50** | **50** | **50** | **50** | **50** | **36** | **Total** |
| RM | 28 | 26 | 28 | 27 | 28 | 27 | 26 | 24 | 22 | 18 | 14 | 10 | 12 | 17 | 22 | 34 | 34 | 39 | 42 | 44 | 45 | 46 | 46 | 47 | 47 | 47 | 47 | 47 | 47 | 33 | RM |
| UHT | 26 | 24 | 26 | 25 | 26 | 26 | 26 | 24 | 22 | 18 | 14 | 10 | 12 | 17 | 22 | 34 | 34 | 39 | 42 | 44 | 45 | 46 | 46 | 47 | 47 | 47 | 47 | 47 | 47 | 33 | UHT |
| IF | 23 | 20 | 20 | 20 | 21 | 20 | 19 | 17 | 16 | 14 | 10 | 8 | 11 | 18 | 25 | 37 | 37 | 42 | 44 | 46 | 47 | 48 | 48 | 48 | 48 | 48 | 48 | 48 | 48 | 34 | IF |

| Position | 121 | 122 | 123 | 124 | 125 | 126 | 127 | 128 | 129 | 130 | 131 | 132 | 133 | 134 | 135 | 136 | 137 | 138 | 139 | 140 | 141 | 142 | 143 | 144 | 145 | 146 | 147 | 148 | 149 | 150 | Position |
|---|---|---|---|---|---|---|---|---|---|---|---|---|---|---|---|---|---|---|---|---|---|---|---|---|---|---|---|---|---|---|---|
| AA | H | S | M | K | E | G | I | H | A | Q | Q | K | E | P | M | I | G | V | N | Q | E | L | A | Y | F | Y | P | E | L | F | AA |
| **Total** | **36** | **33** | **29** | **8** | **4** | **4** | **4** | **4** | **4** | **4** | **5** | **5** | **4** | **4** | **4** | **4** | **4** | **4** | **4** | **4** | **4** | **4** | **4** | **4** | **2** | **2** | **2** | **2** | **2** | **2** | **Total** |
| RM | 33 | 31 | 27 | 8 | 4 | 4 | 4 | 4 | 4 | 4 | 4 | 3 | 2 | 2 | 2 | 2 | 2 | 2 | 2 | 2 | 2 | 2 | 2 | 2 | 2 | 2 | 2 | 2 | 2 | 2 | RM |
| UHT | 33 | 31 | 27 | 8 | 4 | 4 | 4 | 4 | 4 | 4 | 4 | 3 | 2 | 2 | 2 | 2 | 2 | 2 | 2 | 2 | 2 | 2 | 2 | 2 | 2 | 2 | 2 | 2 | 2 | 2 | UHT |
| IF | 34 | 31 | 27 | 8 | 3 | 3 | 3 | 3 | 3 | 3 | 4 | 4 | 3 | 3 | 3 | 3 | 3 | 3 | 3 | 3 | 3 | 3 | 3 | 3 | 1 | 1 | 1 | 1 | 1 | 1 | IF |

| Position | 151 | 152 | 153 | 154 | 155 | 156 | 157 | 158 | 159 | 160 | 161 | 162 | 163 | 164 | 165 | 166 | 167 | 168 | 169 | 170 | 171 | 172 | 173 | 174 | 175 | 176 | 177 | 178 | 179 | 180 | Position |
|---|---|---|---|---|---|---|---|---|---|---|---|---|---|---|---|---|---|---|---|---|---|---|---|---|---|---|---|---|---|---|---|
| AA | R | Q | F | Y | Q | L | D | A | Y | P | S | G | A | W | Y | Y | V | P | L | G | T | Q | Y | T | D | A | P | S | F | S | AA |
| **Total** | **1** | **1** | **1** | **1** | **0** | **0** | **0** | **0** | **0** | **0** | **0** | **0** | **0** | **0** | **0** | **2** | **2** | **2** | **2** | **2** | **3** | **6** | **6** | **9** | **9** | **13** | **15** | **17** | **19** | **26** | **Total** |
| RM | 1 | 1 | 1 | 1 | | | | | | | | | | | | 2 | 2 | 2 | 2 | 2 | 2 | 5 | 5 | 8 | 8 | 11 | 11 | 13 | 14 | 21 | RM |
| UHT | 1 | 1 | 1 | 1 | | | | | | | | | | | | 2 | 2 | 2 | 2 | 2 | 3 | 6 | 6 | 9 | 9 | 13 | 15 | 17 | 19 | 26 | UHT |
| IF | 1 | 1 | 1 | 1 | | | | | | | | | | | | 2 | 2 | 2 | 2 | 2 | 2 | 5 | 5 | 8 | 8 | 11 | 11 | 13 | 14 | 20 | IF |

| Position | 181 | 182 | 183 | 184 | 185 | 186 | 187 | 188 | 189 | 190 | 191 | 192 | 193 | 194 | 195 | 196 | 197 | 198 | 199 | Position |
|---|---|---|---|---|---|---|---|---|---|---|---|---|---|---|---|---|---|---|---|---|
| AA | D | I | P | N | P | I | G | S | E | N | S | E | K | T | T | M | P | L | W | AA |
| **Total** | **30** | **36** | **38** | **42** | **48** | **51** | **51** | **53** | **55** | **57** | **58** | **59** | **60** | **57** | **55** | **55** | **55** | **43** | **41** | **Total** |
| RM | 25 | 29 | 31 | 35 | 39 | 42 | 42 | 44 | 44 | 44 | 46 | 46 | 46 | 44 | 42 | 42 | 42 | 30 | 29 | RM |
| UHT | 30 | 36 | 38 | 41 | 47 | 50 | 50 | 52 | 54 | 56 | 57 | 58 | 59 | 56 | 54 | 54 | 54 | 43 | 41 | UHT |
| IF | 24 | 28 | 30 | 34 | 38 | 41 | 41 | 43 | 45 | 45 | 47 | 47 | 47 | 46 | 44 | 44 | 44 | 32 | 31 | IF |

**Figure S3:** Protein sequence of $\alpha_{S1}$-casein without the signal peptide. The numbers below the sequence indicate how many peptides containing this specific residue were detected over all samples (total) and in RM (sequence coverage 83.9 %), UHT milk (sequence coverage 83.9 %), and IF (sequence coverage 89.4 %). AA denotes amino acid.

| Position | 1 | 2 | 3 | 4 | 5 | 6 | 7 | 8 | 9 | 10 | 11 | 12 | 13 | 14 | 15 | 16 | 17 | 18 | 19 | 20 | 21 | 22 | 23 | 24 | 25 | 26 | 27 | 28 | 29 | 30 | Position |
|---|---|---|---|---|---|---|---|---|---|---|---|---|---|---|---|---|---|---|---|---|---|---|---|---|---|---|---|---|---|---|---|
| AA | K | N | T | M | E | H | V | S | S | S | E | E | S | I | I | S | Q | E | T | Y | K | Q | E | K | N | M | A | I | N | P | AA |
| Total | 9 | 9 | 9 | 9 | 9 | 9 | 9 | 9 | 10 | 10 | 11 | 11 | 12 | 15 | 15 | 15 | 15 | 15 | 15 | 15 | 13 | 6 | 5 | 4 | 0 | 0 | 0 | 0 | 0 | 0 | Total |
| RM | 9 | 9 | 9 | 9 | 9 | 9 | 9 | 9 | 10 | 10 | 10 | 10 | 10 | 13 | 13 | 13 | 13 | 13 | 13 | 13 | 11 | 6 | 5 | 4 | | | | | | | RM |
| UHT | 9 | 9 | 9 | 9 | 9 | 9 | 9 | 9 | 10 | 10 | 10 | 10 | 10 | 13 | 13 | 13 | 13 | 13 | 13 | 13 | 11 | 6 | 5 | 4 | | | | | | | UHT |
| IF | 9 | 9 | 9 | 9 | 9 | 9 | 9 | 9 | 10 | 10 | 11 | 11 | 12 | 15 | 15 | 15 | 15 | 15 | 15 | 15 | 13 | 6 | 5 | 4 | | | | | | | IF |

| Position | 31 | 32 | 33 | 34 | 35 | 36 | 37 | 38 | 39 | 40 | 41 | 42 | 43 | 44 | 45 | 46 | 47 | 48 | 49 | 50 | 51 | 52 | 53 | 54 | 55 | 56 | 57 | 58 | 59 | 60 | Position |
|---|---|---|---|---|---|---|---|---|---|---|---|---|---|---|---|---|---|---|---|---|---|---|---|---|---|---|---|---|---|---|---|
| AA | S | K | E | N | L | C | S | T | F | C | K | E | V | V | R | N | A | N | E | E | E | Y | S | I | G | S | S | S | E | E | AA |
| Total | 0 | 0 | 0 | 0 | 0 | 0 | 0 | 0 | 0 | 0 | 0 | 0 | 0 | 0 | 0 | 0 | 0 | 0 | 0 | 0 | 0 | 0 | 0 | 0 | 0 | 0 | 0 | 0 | 0 | 0 | Total |
| RM | | | | | | | | | | | | | | | | | | | | | | | | | | | | | | | RM |
| UHT | | | | | | | | | | | | | | | | | | | | | | | | | | | | | | | UHT |
| IF | | | | | | | | | | | | | | | | | | | | | | | | | | | | | | | IF |

| Position | 61 | 62 | 63 | 64 | 65 | 66 | 67 | 68 | 69 | 70 | 71 | 72 | 73 | 74 | 75 | 76 | 77 | 78 | 79 | 80 | 81 | 82 | 83 | 84 | 85 | 86 | 87 | 88 | 89 | 90 | Position |
|---|---|---|---|---|---|---|---|---|---|---|---|---|---|---|---|---|---|---|---|---|---|---|---|---|---|---|---|---|---|---|---|
| AA | S | A | E | V | A | T | E | E | V | K | I | T | V | D | D | K | H | Y | Q | K | A | L | N | E | I | N | Q | F | Y | Q | AA |
| Total | 0 | 0 | 0 | 0 | 0 | 0 | 0 | 0 | 0 | 0 | 2 | 2 | 2 | 2 | 2 | 2 | 2 | 2 | 1 | 0 | 4 | 4 | 4 | 4 | 4 | 4 | 4 | 5 | 6 | 6 | Total |
| RM | | | | | | | | | | | 2 | 2 | 2 | 2 | 2 | 2 | 2 | 2 | 1 | | | | | | | | | | | | RM |
| UHT | | | | | | | | | | | 2 | 2 | 2 | 2 | 2 | 2 | 2 | 2 | 1 | | | | | | | | | | | | UHT |
| IF | | | | | | | | | | | 2 | 2 | 2 | 2 | 2 | 2 | 2 | 2 | 1 | | 4 | 4 | 4 | 4 | 4 | 4 | 4 | 5 | 6 | 6 | IF |

| Position | 91 | 92 | 93 | 94 | 95 | 96 | 97 | 98 | 99 | 100 | 101 | 102 | 103 | 104 | 105 | 106 | 107 | 108 | 109 | 110 | 111 | 112 | 113 | 114 | 115 | 116 | 117 | 118 | 119 | 120 | Position |
|---|---|---|---|---|---|---|---|---|---|---|---|---|---|---|---|---|---|---|---|---|---|---|---|---|---|---|---|---|---|---|---|
| AA | K | F | P | Q | Y | L | Q | Y | L | Y | Q | G | P | I | V | L | N | P | W | D | Q | V | K | R | N | A | V | P | I | T | AA |
| Total | 6 | 6 | 6 | 6 | 6 | 4 | 4 | 5 | 4 | 8 | 10 | 12 | 12 | 12 | 13 | 13 | 13 | 13 | 13 | 13 | 13 | 13 | 11 | 5 | 7 | 5 | 5 | 5 | 5 | 5 | Total |
| RM | | | | | | | | 1 | 3 | 5 | 7 | 9 | 9 | 9 | 10 | 10 | 10 | 10 | 10 | 10 | 10 | 10 | 9 | 3 | 4 | 4 | 4 | 4 | 4 | 4 | RM |
| UHT | | | | | | | | 1 | 3 | 5 | 7 | 8 | 8 | 8 | 9 | 9 | 9 | 9 | 9 | 9 | 9 | 9 | 9 | 3 | 5 | 5 | 5 | 5 | 5 | 5 | UHT |
| IF | 6 | 6 | 6 | 6 | 6 | 4 | 4 | 4 | 3 | 7 | 9 | 10 | 10 | 10 | 11 | 11 | 11 | 11 | 11 | 11 | 11 | 11 | 10 | 5 | 7 | 5 | 5 | 5 | 5 | 5 | IF |

| Position | 121 | 122 | 123 | 124 | 125 | 126 | 127 | 128 | 129 | 130 | 131 | 132 | 133 | 134 | 135 | 136 | 137 | 138 | 139 | 140 | 141 | 142 | 143 | 144 | 145 | 146 | 147 | 148 | 149 | 150 | Position |
|---|---|---|---|---|---|---|---|---|---|---|---|---|---|---|---|---|---|---|---|---|---|---|---|---|---|---|---|---|---|---|---|
| AA | P | T | L | N | R | E | Q | L | S | T | S | E | E | N | S | K | K | T | V | D | M | E | S | T | E | V | F | T | K | K | AA |
| Total | 5 | 5 | 9 | 9 | 9 | 9 | 9 | 9 | 8 | 8 | 8 | 8 | 8 | 8 | 9 | 6 | 9 | 12 | 16 | 18 | 21 | 21 | 21 | 21 | 21 | 21 | 21 | 21 | 21 | 8 | Total |
| RM | 4 | 4 | 8 | 8 | 8 | 8 | 8 | 8 | 8 | 8 | 8 | 8 | 8 | 8 | 9 | 6 | 9 | 12 | 16 | 18 | 20 | 20 | 20 | 20 | 20 | 20 | 20 | 20 | 20 | 8 | RM |
| UHT | 5 | 5 | 9 | 9 | 9 | 9 | 9 | 9 | 8 | 8 | 8 | 8 | 8 | 8 | 9 | 6 | 9 | 12 | 16 | 18 | 20 | 20 | 20 | 20 | 20 | 20 | 20 | 20 | 20 | 8 | UHT |
| IF | 5 | 5 | 9 | 9 | 9 | 9 | 9 | 9 | 8 | 8 | 8 | 8 | 8 | 8 | 9 | 6 | 9 | 11 | 13 | 14 | 17 | 17 | 17 | 17 | 17 | 17 | 17 | 17 | 17 | 5 | IF |

| Position | 151 | 152 | 153 | 154 | 155 | 156 | 157 | 158 | 159 | 160 | 161 | 162 | 163 | 164 | 165 | 166 | 167 | 168 | 169 | 170 | 171 | 172 | 173 | 174 | 175 | 176 | 177 | 178 | 179 | 180 | Position |
|---|---|---|---|---|---|---|---|---|---|---|---|---|---|---|---|---|---|---|---|---|---|---|---|---|---|---|---|---|---|---|---|
| AA | T | K | L | T | E | E | E | K | N | R | L | N | F | L | K | K | I | S | Q | R | Y | Q | K | F | A | L | P | Q | Y | L | AA |
| Total | 7 | 10 | 13 | 16 | 17 | 17 | 17 | 17 | 17 | 17 | 17 | 16 | 15 | 11 | 6 | 1 | 1 | 1 | 1 | 1 | 3 | 5 | 5 | 7 | 8 | 10 | 10 | 10 | 10 | 7 | Total |
| RM | 7 | 10 | 13 | 16 | 17 | 17 | 17 | 17 | 17 | 17 | 17 | 15 | 14 | 10 | 5 | | | | | | 1 | 2 | 2 | 4 | 5 | 7 | 7 | 7 | 7 | 7 | RM |
| UHT | 7 | 10 | 13 | 16 | 17 | 17 | 17 | 17 | 17 | 17 | 17 | 15 | 14 | 10 | 5 | | | | | | 1 | 2 | 2 | 4 | 5 | 7 | 7 | 7 | 7 | 7 | UHT |
| IF | 7 | 9 | 12 | 15 | 16 | 16 | 16 | 16 | 16 | 16 | 16 | 15 | 14 | 10 | 5 | 1 | 1 | 1 | 1 | 1 | 3 | 5 | 5 | 6 | 6 | 7 | 7 | 7 | 7 | 4 | IF |

| Position | 181 | 182 | 183 | 184 | 185 | 186 | 187 | 188 | 189 | 190 | 191 | 192 | 193 | 194 | 195 | 196 | 197 | 198 | 199 | 200 | 201 | 202 | 203 | 204 | 205 | 206 | 207 | Position |
|---|---|---|---|---|---|---|---|---|---|---|---|---|---|---|---|---|---|---|---|---|---|---|---|---|---|---|---|---|
| AA | K | T | V | Y | Q | H | Q | K | A | M | K | P | W | I | Q | P | K | T | K | V | I | P | Y | V | R | Y | L | AA |
| Total | 5 | 4 | 4 | 3 | 3 | 2 | 2 | 1 | 6 | 6 | 7 | 7 | 7 | 7 | 7 | 7 | 5 | 8 | 8 | 10 | 11 | 11 | 11 | 10 | 9 | 7 | 4 | Total |
| RM | 5 | 4 | 4 | 3 | 3 | 2 | 2 | 1 | 4 | 4 | 5 | 5 | 5 | 5 | 5 | 5 | 3 | 7 | 7 | 9 | 10 | 10 | 10 | 10 | 9 | 7 | 4 | RM |
| UHT | 5 | 4 | 4 | 3 | 3 | 2 | 2 | 1 | 4 | 4 | 5 | 5 | 5 | 5 | 5 | 5 | 3 | 7 | 7 | 9 | 10 | 10 | 10 | 10 | 9 | 7 | 4 | UHT |
| IF | 2 | 1 | 1 | 1 | 1 | | | | 4 | 4 | 4 | 4 | 4 | 4 | 4 | 4 | 3 | 6 | 6 | 8 | 9 | 9 | 9 | 8 | 7 | 6 | 4 | IF |

**Figure S4**: Protein sequence of $\alpha_{S2}$-casein without the signal peptide. The numbers below the sequence indicate how many peptides containing this specific residue were detected over all samples (total) and in RM (sequence coverage 66.7 %), UHT milk (sequence coverage 66.7 %), and IF (sequence coverage 75.8 %). AA denotes amino acid.

| Position | 1 | 2 | 3 | 4 | 5 | 6 | 7 | 8 | 9 | 10 | 11 | 12 | 13 | 14 | 15 | 16 | 17 | 18 | 19 | 20 | 21 | 22 | 23 | 24 | 25 | 26 | 27 | 28 | 29 | 30 | Position |
|---|---|---|---|---|---|---|---|---|---|---|---|---|---|---|---|---|---|---|---|---|---|---|---|---|---|---|---|---|---|---|---|
| **AA** | **I** | **L** | **N** | **K** | **P** | **E** | **D** | **E** | **T** | **H** | **L** | **E** | **A** | **Q** | **P** | **T** | **D** | **A** | **S** | **A** | **Q** | **F** | **I** | **R** | **N** | **L** | **Q** | **I** | **S** | **N** | **AA** |
| **Total** | **15** | **18** | **18** | **18** | **18** | **18** | **18** | **18** | **18** | **18** | **18** | **18** | **18** | **18** | **18** | **18** | **17** | **16** | **15** | **15** | **14** | **13** | **9** | **8** | **5** | **4** | **2** | **1** | **1** | **1** | **Total** |
| RM | 14 | 17 | 17 | 17 | 17 | 17 | 17 | 17 | 17 | 17 | 17 | 17 | 15 | 15 | 15 | 15 | 14 | 13 | 12 | 12 | 11 | 10 | 8 | 7 | 4 | 3 | 2 | 1 | 1 | 1 | RM |
| UHT | 15 | 18 | 18 | 18 | 18 | 18 | 18 | 18 | 18 | 18 | 18 | 17 | 15 | 15 | 15 | 15 | 14 | 13 | 12 | 12 | 11 | 10 | 8 | 7 | 4 | 3 | 2 | 1 | 1 | 1 | UHT |
| IF | 15 | 17 | 17 | 17 | 17 | 17 | 17 | 17 | 17 | 17 | 17 | 17 | 17 | 17 | 17 | 17 | 16 | 15 | 14 | 14 | 13 | 12 | 8 | 7 | 5 | 4 | 2 | 1 | 1 | 1 | IF |

| Position | 31 | 32 | 33 | 34 | 35 | 36 | 37 | 38 | 39 | 40 | 41 | 42 | 43 | 44 | 45 | 46 | 47 | 48 | 49 | 50 | 51 | 52 | 53 | 54 | 55 | 56 | 57 | 58 | 59 | 60 | Position |
|---|---|---|---|---|---|---|---|---|---|---|---|---|---|---|---|---|---|---|---|---|---|---|---|---|---|---|---|---|---|---|---|
| **AA** | **E** | **D** | **L** | **S** | **K** | **E** | **P** | **S** | **I** | **S** | **R** | **E** | **D** | **L** | **I** | **S** | **K** | **E** | **Q** | **I** | **V** | **I** | **R** | **S** | **S** | **R** | **Q** | **P** | **Q** | **S** | **AA** |
| **Total** | **1** | **1** | **1** | **1** | **1** | **1** | **1** | **1** | **1** | **1** | **1** | **1** | **1** | **2** | **2** | **2** | **2** | **2** | **2** | **2** | **2** | **2** | **2** | **8** | **11** | **11** | **16** | **16** | **16** | **19** | **Total** |
| RM | 1 | 1 | 1 | 1 | 1 | 1 | 1 | 1 | 1 | 1 | 1 | 1 | 1 | 2 | 2 | 2 | 2 | 2 | 2 | 2 | 2 | 2 | 2 | 8 | 11 | 11 | 16 | 16 | 16 | 16 | RM |
| UHT | 1 | 1 | 1 | 1 | 1 | 1 | 1 | 1 | 1 | 1 | 1 | 1 | 1 | 2 | 2 | 2 | 2 | 2 | 2 | 2 | 2 | 2 | 2 | 8 | 11 | 11 | 16 | 16 | 16 | 17 | UHT |
| IF | 1 | 1 | 1 | 1 | 1 | 1 | 1 | 1 | 1 | 1 | 1 | 1 | 1 | 2 | 2 | 2 | 2 | 2 | 2 | 2 | 2 | 2 | 2 | 8 | 11 | 11 | 14 | 14 | 14 | 17 | IF |

| Position | 61 | 62 | 63 | 64 | 65 | 66 | 67 | 68 | 69 | 70 | 71 | 72 | 73 | 74 | 75 | 76 | 77 | 78 | 79 | 80 | 81 | 82 | 83 | 84 | 85 | 86 | 87 | 88 | 89 | 90 | Position |
|---|---|---|---|---|---|---|---|---|---|---|---|---|---|---|---|---|---|---|---|---|---|---|---|---|---|---|---|---|---|---|---|
| **AA** | **Q** | **N** | **P** | **K** | **L** | **P** | **L** | **S** | **I** | **L** | **K** | **E** | **K** | **H** | **L** | **R** | **N** | **A** | **T** | **L** | **G** | **S** | **E** | **E** | **T** | **T** | **E** | **H** | **T** | **P** | **AA** |
| **Total** | **19** | **20** | **21** | **21** | **24** | **24** | **23** | **21** | **17** | **17** | **11** | **8** | **7** | **4** | **3** | **0** | **0** | **0** | **0** | **0** | **0** | **0** | **0** | **0** | **0** | **0** | **0** | **0** | **0** | **0** | **Total** |
| RM | 16 | 16 | 16 | 16 | 19 | 19 | 18 | 17 | 14 | 14 | 11 | 8 | 7 | 4 | 3 | | | | | | | | | | | | | | | | RM |
| UHT | 17 | 17 | 17 | 17 | 20 | 20 | 19 | 17 | 14 | 14 | 11 | 8 | 7 | 4 | 3 | | | | | | | | | | | | | | | | UHT |
| IF | 17 | 18 | 19 | 19 | 20 | 20 | 19 | 17 | 13 | 13 | 7 | 5 | 4 | 2 | 2 | | | | | | | | | | | | | | | | IF |

| Position | 91 | 92 | 93 | 94 | 95 | 96 | 97 | 98 | 99 | 100 | 101 | 102 | 103 | 104 | 105 | 106 | 107 | 108 | 109 | 110 | 111 | 112 | 113 | 114 | 115 | 116 | 117 | 118 | 119 | 120 | Position |
|---|---|---|---|---|---|---|---|---|---|---|---|---|---|---|---|---|---|---|---|---|---|---|---|---|---|---|---|---|---|---|---|
| **AA** | **S** | **D** | **A** | **S** | **T** | **T** | **E** | **G** | **K** | **L** | **M** | **E** | **L** | **G** | **H** | **K** | **I** | **M** | **R** | **N** | **L** | **E** | **N** | **T** | **V** | **K** | **E** | **T** | **I** | **K** | **AA** |
| **Total** | **0** | **0** | **0** | **0** | **0** | **0** | **0** | **0** | **0** | **0** | **0** | **0** | **0** | **0** | **0** | **0** | **0** | **0** | **1** | **1** | **2** | **3** | **4** | **4** | **4** | **4** | **4** | **4** | **4** | **4** | **Total** |
| RM | | | | | | | | | | | | | | | | | | | | | | | | | | | | | | | RM |
| UHT | | | | | | | | | | | | | | | | | | | 1 | 1 | 1 | 1 | 1 | 1 | 1 | 1 | 1 | 1 | 1 | 1 | UHT |
| IF | | | | | | | | | | | | | | | | | | | 1 | 1 | 2 | 3 | 4 | 4 | 4 | 4 | 4 | 4 | 4 | 4 | IF |

| Position | 121 | 122 | 123 | 124 | 125 | 126 | 127 | 128 | 129 | 130 | 131 | 132 | 133 | 134 | 135 | Position |
|---|---|---|---|---|---|---|---|---|---|---|---|---|---|---|---|---|
| **AA** | **Y** | **L** | **K** | **S** | **L** | **F** | **S** | **H** | **A** | **F** | **E** | **V** | **V** | **K** | **T** | **AA** |
| **Total** | **4** | **1** | **2** | **3** | **3** | **4** | **5** | **6** | **6** | **6** | **6** | **6** | **6** | **6** | **6** | **Total** |
| RM | | | | | | | 1 | 2 | 2 | 2 | 2 | 2 | 2 | 2 | 2 | RM |
| UHT | 1 | | | 1 | 1 | 1 | 2 | 3 | 3 | 3 | 3 | 3 | 3 | 3 | 3 | UHT |
| IF | 4 | 1 | 2 | 3 | 3 | 4 | 5 | 6 | 6 | 6 | 6 | 6 | 6 | 6 | 6 | IF |

**Figure S5**: Protein sequence of GlyCAM-1 without the signal peptide. The numbers below the sequence indicate how many peptides containing this specific residue were detected over all samples (total) and in RM (sequence coverage 62.2 %), UHT milk (sequence coverage 74.1 %), and IF (sequence coverage 75.6 %). AA denotes amino acid.

| Position | 1 | 2 | 3 | 4 | 5 | 6 | 7 | 8 | 9 | 10 | 11 | 12 | 13 | 14 | 15 | 16 | 17 | 18 | 19 | 20 | 21 | 22 | 23 | 24 | 25 | 26 | 27 | 28 | 29 | 30 | Position |
|---|---|---|---|---|---|---|---|---|---|---|---|---|---|---|---|---|---|---|---|---|---|---|---|---|---|---|---|---|---|---|---|
| **AA** | Q | E | Q | N | Q | E | Q | P | I | R | C | E | K | D | E | R | F | F | S | D | K | I | A | K | Y | I | P | I | Q | Y | **AA** |
| **Total** | **0** | **0** | **0** | **0** | **0** | **0** | **0** | **0** | **0** | **0** | **0** | **0** | **0** | **1** | **1** | **1** | **1** | **1** | **1** | **1** | **1** | **1** | **1** | **1** | **0** | **0** | **0** | **0** | **0** | **0** | **Total** |
| RM | | | | | | | | | | | | | | 1 | 1 | 1 | 1 | 1 | 1 | 1 | 1 | 1 | 1 | 1 | | | | | | | RM |
| UHT | | | | | | | | | | | | | | 1 | 1 | 1 | 1 | 1 | 1 | 1 | 1 | 1 | 1 | 1 | | | | | | | UHT |
| IF | | | | | | | | | | | | | | 1 | 1 | 1 | 1 | 1 | 1 | 1 | 1 | 1 | 1 | 1 | | | | | | | IF |

| Position | 31 | 32 | 33 | 34 | 35 | 36 | 37 | 38 | 39 | 40 | 41 | 42 | 43 | 44 | 45 | 46 | 47 | 48 | 49 | 50 | 51 | 52 | 53 | 54 | 55 | 56 | 57 | 58 | 59 | 60 | Position |
|---|---|---|---|---|---|---|---|---|---|---|---|---|---|---|---|---|---|---|---|---|---|---|---|---|---|---|---|---|---|---|---|
| **AA** | V | L | S | R | Y | P | S | Y | G | L | N | Y | Y | Q | Q | K | P | V | A | L | I | N | N | Q | F | L | P | Y | P | Y | **AA** |
| **Total** | **4** | **4** | **7** | **7** | **7** | **7** | **7** | **7** | **6** | **6** | **5** | **5** | **7** | **5** | **5** | **5** | **5** | **5** | **6** | **6** | **4** | **5** | **5** | **3** | **3** | **3** | **3** | **3** | **3** | **3** | **Total** |
| RM | | | | | | | | | | | | | | | | | | | 1 | 1 | 1 | 1 | 1 | 1 | 1 | 1 | 1 | 1 | 1 | 1 | RM |
| UHT | | | | | | | | | | | | | | | | | | | 1 | 1 | 1 | 1 | 1 | 1 | 1 | 1 | 1 | 1 | 1 | 1 | UHT |
| IF | 4 | 4 | 7 | 7 | 7 | 7 | 7 | 7 | 6 | 6 | 5 | 5 | 7 | 5 | 5 | 5 | 5 | 5 | 5 | 5 | 3 | 4 | 4 | 2 | 2 | 2 | 2 | 2 | 2 | 2 | IF |

| Position | 61 | 62 | 63 | 64 | 65 | 66 | 67 | 68 | 69 | 70 | 71 | 72 | 73 | 74 | 75 | 76 | 77 | 78 | 79 | 80 | 81 | 82 | 83 | 84 | 85 | 86 | 87 | 88 | 89 | 90 | Position |
|---|---|---|---|---|---|---|---|---|---|---|---|---|---|---|---|---|---|---|---|---|---|---|---|---|---|---|---|---|---|---|---|
| **AA** | Y | A | K | P | A | A | V | R | S | P | A | Q | I | L | Q | W | Q | V | L | S | N | T | V | P | A | K | S | C | Q | A | **AA** |
| **Total** | **3** | **3** | **3** | **3** | **3** | **2** | **2** | **2** | **3** | **3** | **3** | **3** | **3** | **3** | **2** | **2** | **1** | **1** | **1** | **0** | **0** | **0** | **0** | **0** | **0** | **0** | **0** | **0** | **0** | **0** | **Total** |
| RM | 1 | 1 | 1 | 1 | 1 | | | | 1 | 1 | 1 | 1 | 1 | 1 | 1 | 1 | 1 | 1 | 1 | | | | | | | | | | | | RM |
| UHT | 1 | 1 | 1 | 1 | 1 | | | | | | | | | | | | | | | | | | | | | | | | | | UHT |
| IF | 2 | 2 | 2 | 2 | 2 | 2 | 2 | 2 | 2 | 2 | 2 | 2 | 2 | 2 | 1 | 1 | | | | | | | | | | | | | | | IF |

| Position | 91 | 92 | 93 | 94 | 95 | 96 | 97 | 98 | 99 | 100 | 101 | 102 | 103 | 104 | 105 | 106 | 107 | 108 | 109 | 110 | 111 | 112 | 113 | 114 | 115 | 116 | 117 | 118 | 119 | 120 | Position |
|---|---|---|---|---|---|---|---|---|---|---|---|---|---|---|---|---|---|---|---|---|---|---|---|---|---|---|---|---|---|---|---|
| **AA** | Q | P | T | T | M | A | R | H | P | H | P | H | L | S | F | M | A | I | P | P | K | K | N | Q | D | K | T | E | I | P | **AA** |
| **Total** | **0** | **0** | **0** | **0** | **0** | **0** | **0** | **0** | **0** | **0** | **0** | **0** | **0** | **0** | **0** | **3** | **3** | **3** | **3** | **3** | **3** | **3** | **3** | **4** | **4** | **4** | **4** | **4** | **4** | **4** | **Total** |
| RM | | | | | | | | | | | | | | | | | | | | | | | | | | | | | | | RM |
| UHT | | | | | | | | | | | | | | | | | | | | | | | | | | | | | | | UHT |
| IF | | | | | | | | | | | | | | | | 3 | 3 | 3 | 3 | 3 | 3 | 3 | 3 | 4 | 4 | 4 | 4 | 4 | 4 | 4 | IF |

| Position | 121 | 122 | 123 | 124 | 125 | 126 | 127 | 128 | 129 | 130 | 131 | 132 | 133 | 134 | 135 | 136 | 137 | 138 | 139 | 140 | 141 | 142 | 143 | 144 | 145 | 146 | 147 | 148 | 149 | 150 | Position |
|---|---|---|---|---|---|---|---|---|---|---|---|---|---|---|---|---|---|---|---|---|---|---|---|---|---|---|---|---|---|---|---|
| **AA** | T | I | N | T | I | A | S | G | E | P | T | S | T | P | T | T | E | A | V | E | S | T | V | A | T | L | E | D | S | P | **AA** |
| **Total** | **4** | **4** | **4** | **3** | **3** | **3** | **3** | **3** | **3** | **3** | **3** | **3** | **3** | **3** | **3** | **3** | **3** | **1** | **1** | **1** | **1** | **1** | **1** | **1** | **1** | **1** | **1** | **2** | **4** | **5** | **Total** |
| RM | | | | | | | | | | | | | | | | | | | | | | | | | | | | | 2 | 3 | RM |
| UHT | | | | | | | | | | | | | | | | | | | | | | | | | | | | 1 | 3 | 4 | UHT |
| IF | 4 | 4 | 4 | 3 | 3 | 3 | 3 | 3 | 3 | 3 | 3 | 3 | 3 | 3 | 3 | 3 | 3 | 1 | 1 | 1 | 1 | 1 | 1 | 1 | 1 | 1 | 1 | 2 | 4 | 5 | IF |

| Position | 151 | 152 | 153 | 154 | 155 | 156 | 157 | 158 | 159 | 160 | 161 | 162 | 163 | 164 | 165 | 166 | 167 | 168 | 169 | Position |
|---|---|---|---|---|---|---|---|---|---|---|---|---|---|---|---|---|---|---|---|---|
| **AA** | E | V | I | E | S | P | P | E | I | N | T | V | Q | V | T | S | T | A | V | **AA** |
| **Total** | **6** | **9** | **9** | **9** | **10** | **10** | **10** | **10** | **11** | **11** | **11** | **10** | **10** | **10** | **10** | **10** | **10** | **10** | **10** | **Total** |
| RM | 3 | 4 | 4 | 4 | 4 | 4 | 4 | 4 | 4 | 4 | 4 | 3 | 3 | 3 | 3 | 3 | 3 | 3 | 3 | RM |
| UHT | 5 | 6 | 6 | 6 | 7 | 7 | 7 | 7 | 7 | 7 | 8 | 8 | 8 | 8 | 8 | 8 | 8 | 8 | 8 | UHT |
| IF | 6 | 9 | 9 | 9 | 10 | 10 | 10 | 10 | 11 | 11 | 11 | 10 | 10 | 10 | 10 | 10 | 10 | 10 | 10 | IF |

**Figure S6**: Protein sequence of κ-casein without the signal peptide. The numbers below the sequence indicate how many peptides containing this specific residue were detected over all samples (total) and in RM (sequence coverage 36.1 %), UHT milk (sequence coverage 29.6 %), and IF (sequence coverage 71.6 %). AA denotes amino acid.

| Position | 1 | 2 | 3 | 4 | 5 | 6 | 7 | 8 | 9 | 10 | 11 | 12 | 13 | 14 | 15 | 16 | 17 | 18 | 19 | 20 | 21 | 22 | 23 | 24 | 25 | 26 | 27 | 28 | 29 | 30 | Position |
|---|---|---|---|---|---|---|---|---|---|---|---|---|---|---|---|---|---|---|---|---|---|---|---|---|---|---|---|---|---|---|---|
| AA | R | P | K | H | P | I | K | H | Q | G | L | P | Q | E | V | L | N | E | N | L | L | R | F | F | V | A | P | F | P | E | AA |
| Total (unmod) | 2 | 2 | 2 | 6 | 6 | 6 | 8 | 10 | 14 | 21 | 25 | 25 | 27 | 31 | 33 | 36 | 37 | 35 | 34 | 33 | 31 | 24 | 22 | 18 | 22 | 30 | 33 | 36 | 36 | 36 | Total (unmod) |
| Total (mod) | 10 | 10 | 10 | 10 | 10 | 10 | 10 | 10 | 10 | 10 | 10 | 10 | 10 | 9 | 9 | 9 | 8 | 8 | 8 | 8 | 7 | 6 | 6 | 11 | 17 | 20 | 20 | 21 | 21 | 21 | Total (mod) |
| RM | 4 | 4 | 4 | 4 | 4 | 4 | 4 | 4 | 4 | 4 | 4 | 4 | 4 | 4 | 4 | 4 | 4 | 4 | 4 | 4 | 4 | 3 | 1 | | | | | | | | RM |
| UHT | 5 | 5 | 5 | 5 | 5 | 5 | 5 | 5 | 5 | 5 | 5 | 5 | 5 | 5 | 5 | 5 | 5 | 5 | 5 | 5 | 4 | 3 | 2 | 1 | 2 | 3 | 3 | 3 | 3 | 3 | UHT |
| IF | 10 | 10 | 10 | 10 | 10 | 10 | 10 | 10 | 10 | 10 | 10 | 10 | 10 | 9 | 9 | 9 | 8 | 8 | 8 | 8 | 7 | 6 | 6 | 11 | 17 | 20 | 20 | 21 | 21 | 21 | IF |

| Position | 31 | 32 | 33 | 34 | 35 | 36 | 37 | 38 | 39 | 40 | 41 | 42 | 43 | 44 | 45 | 46 | 47 | 48 | 49 | 50 | 51 | 52 | 53 | 54 | 55 | 56 | 57 | 58 | 59 | 60 | Position |
|---|---|---|---|---|---|---|---|---|---|---|---|---|---|---|---|---|---|---|---|---|---|---|---|---|---|---|---|---|---|---|---|
| AA | V | F | G | K | E | K | V | N | E | L | S | K | D | I | G | S | E | S | T | E | D | Q | A | M | E | D | I | K | Q | M | AA |
| Total (unmod) | 36 | 36 | 34 | 30 | 27 | 23 | 20 | 15 | 15 | 13 | 12 | 8 | 10 | 11 | 11 | 11 | 11 | 11 | 11 | 11 | 11 | 11 | 11 | 10 | 10 | 10 | 10 | 10 | 0 | 0 | Total (unmod) |
| Total (mod) | 21 | 21 | 21 | 21 | 21 | 21 | 16 | 11 | 10 | 9 | 8 | 7 | 0 | 0 | 0 | 0 | 0 | 0 | 0 | 0 | 0 | 0 | 0 | 0 | 0 | 0 | 0 | 0 | 0 | 0 | Total (mod) |
| RM | | | | | | | | | | | | | | | | | | | | | | | | | | | | | | | RM |
| UHT | 3 | 3 | 3 | 3 | 3 | 3 | | | | | | | | | | | | | | | | | | | | | | | | | UHT |
| IF | 21 | 21 | 21 | 21 | 21 | 21 | 16 | 11 | 10 | 9 | 8 | 7 | | | | | | | | | | | | | | | | | | | IF |

| Position | 61 | 62 | 63 | 64 | 65 | 66 | 67 | 68 | 69 | 70 | 71 | 72 | 73 | 74 | 75 | 76 | 77 | 78 | 79 | 80 | 81 | 82 | 83 | 84 | 85 | 86 | 87 | 88 | 89 | 90 | Position |
|---|---|---|---|---|---|---|---|---|---|---|---|---|---|---|---|---|---|---|---|---|---|---|---|---|---|---|---|---|---|---|---|
| AA | E | A | E | S | I | S | S | S | E | E | I | V | P | N | S | V | E | Q | K | H | I | Q | K | E | D | V | P | S | E | R | AA |
| Total (unmod) | 0 | 0 | 0 | 0 | 0 | 0 | 0 | 0 | 1 | 1 | 1 | 1 | 1 | 1 | 1 | 1 | 1 | 1 | 1 | 15 | 20 | 21 | 23 | 24 | 24 | 27 | 27 | 27 | 27 | 26 | Total (unmod) |
| Total (mod) | 0 | 0 | 0 | 0 | 0 | 0 | 0 | 0 | 0 | 0 | 0 | 0 | 0 | 0 | 0 | 0 | 0 | 0 | 0 | 17 | 20 | 20 | 20 | 20 | 20 | 20 | 20 | 20 | 20 | 20 | Total (mod) |
| RM | | | | | | | | | | | | | | | | | | | | 3 | 3 | 3 | 3 | 3 | 3 | 3 | 3 | 3 | 3 | 3 | RM |
| UHT | | | | | | | | | | | | | | | | | | | | 9 | 11 | 11 | 11 | 11 | 11 | 11 | 11 | 11 | 11 | 11 | UHT |
| IF | | | | | | | | | | | | | | | | | | | | 17 | 20 | 20 | 20 | 20 | 20 | 20 | 20 | 20 | 20 | 20 | IF |

| Position | 91 | 92 | 93 | 94 | 95 | 96 | 97 | 98 | 99 | 100 | 101 | 102 | 103 | 104 | 105 | 106 | 107 | 108 | 109 | 110 | 111 | 112 | 113 | 114 | 115 | 116 | 117 | 118 | 119 | 120 | Position |
|---|---|---|---|---|---|---|---|---|---|---|---|---|---|---|---|---|---|---|---|---|---|---|---|---|---|---|---|---|---|---|---|
| AA | Y | L | G | Y | L | E | Q | L | L | R | L | K | K | Y | K | V | P | Q | L | E | I | V | P | N | S | A | E | E | R | L | AA |
| Total (unmod) | 28 | 26 | 28 | 27 | 28 | 27 | 26 | 24 | 22 | 18 | 14 | 10 | 12 | 18 | 25 | 37 | 37 | 42 | 45 | 47 | 48 | 49 | 49 | 50 | 50 | 50 | 50 | 50 | 50 | 36 | Total (unmod) |
| Total (mod) | 17 | 15 | 14 | 13 | 12 | 12 | 11 | 11 | 11 | 10 | 7 | 6 | 12 | 13 | 13 | 13 | 13 | 13 | 13 | 13 | 13 | 13 | 13 | 13 | 13 | 13 | 13 | 13 | 13 | 13 | Total (mod) |
| RM | 2 | 2 | 2 | 2 | 2 | 2 | 2 | 2 | 2 | 2 | 2 | 1 | 3 | 3 | 3 | 3 | 3 | 3 | 3 | 3 | 3 | 3 | 3 | 3 | 3 | 3 | 3 | 3 | 3 | 3 | RM |
| UHT | 9 | 7 | 7 | 6 | 6 | 6 | 6 | 6 | 6 | 6 | 4 | 3 | 9 | 9 | 8 | 8 | 8 | 8 | 8 | 8 | 8 | 8 | 8 | 8 | 8 | 8 | 8 | 8 | 8 | 8 | UHT |
| IF | 17 | 15 | 14 | 13 | 12 | 12 | 11 | 11 | 11 | 10 | 7 | 6 | 12 | 13 | 13 | 13 | 13 | 13 | 13 | 13 | 13 | 13 | 13 | 13 | 13 | 13 | 13 | 13 | 13 | 13 | IF |

| Position | 121 | 122 | 123 | 124 | 125 | 126 | 127 | 128 | 129 | 130 | 131 | 132 | 133 | 134 | 135 | 136 | 137 | 138 | 139 | 140 | 141 | 142 | 143 | 144 | 145 | 146 | 147 | 148 | 149 | 150 | Position |
|---|---|---|---|---|---|---|---|---|---|---|---|---|---|---|---|---|---|---|---|---|---|---|---|---|---|---|---|---|---|---|---|
| AA | H | S | M | K | E | G | I | H | A | Q | Q | K | E | P | M | I | G | V | N | Q | E | L | A | Y | F | Y | P | E | L | F | AA |
| Total (unmod) | 36 | 33 | 29 | 8 | 4 | 4 | 4 | 4 | 4 | 4 | 5 | 5 | 4 | 4 | 4 | 4 | 4 | 4 | 4 | 4 | 4 | 4 | 4 | 4 | 2 | 2 | 2 | 2 | 2 | 2 | Total (unmod) |
| Total (mod) | 13 | 13 | 13 | 3 | 0 | 0 | 0 | 0 | 0 | 0 | 0 | 0 | 0 | 0 | 0 | 0 | 0 | 0 | 0 | 0 | 0 | 0 | 0 | 0 | 0 | 0 | 0 | 0 | 0 | 0 | Total (mod) |
| RM | 3 | 3 | 3 | | | | | | | | | | | | | | | | | | | | | | | | | | | | RM |
| UHT | 8 | 8 | 8 | 2 | | | | | | | | | | | | | | | | | | | | | | | | | | | UHT |
| IF | 13 | 13 | 13 | 3 | | | | | | | | | | | | | | | | | | | | | | | | | | | IF |

| Position | 151 | 152 | 153 | 154 | 155 | 156 | 157 | 158 | 159 | 160 | 161 | 162 | 163 | 164 | 165 | 166 | 167 | 168 | 169 | 170 | 171 | 172 | 173 | 174 | 175 | 176 | 177 | 178 | 179 | 180 | Position |
|---|---|---|---|---|---|---|---|---|---|---|---|---|---|---|---|---|---|---|---|---|---|---|---|---|---|---|---|---|---|---|---|
| AA | R | Q | F | Y | Q | L | D | A | Y | P | S | G | A | W | Y | Y | V | P | L | G | T | Q | Y | T | D | A | P | S | F | S | AA |
| Total (unmod) | 1 | 1 | 1 | 1 | 0 | 0 | 0 | 0 | 0 | 0 | 0 | 0 | 0 | 0 | 0 | 2 | 2 | 2 | 2 | 2 | 3 | 6 | 6 | 9 | 9 | 13 | 15 | 17 | 19 | 26 | Total (unmod) |
| Total (mod) | 0 | 0 | 0 | 0 | 0 | 0 | 0 | 0 | 0 | 0 | 0 | 0 | 0 | 0 | 0 | 0 | 0 | 0 | 0 | 0 | 0 | 0 | 0 | 0 | 0 | 0 | 0 | 0 | 0 | 0 | Total (mod) |
| RM | | | | | | | | | | | | | | | | | | | | | | | | | | | | | | | RM |
| UHT | | | | | | | | | | | | | | | | | | | | | | | | | | | | | | | UHT |
| IF | | | | | | | | | | | | | | | | | | | | | | | | | | | | | | | IF |

| Position | 181 | 182 | 183 | 184 | 185 | 186 | 187 | 188 | 189 | 190 | 191 | 192 | 193 | 194 | 195 | 196 | 197 | 198 | 199 | Position |
|---|---|---|---|---|---|---|---|---|---|---|---|---|---|---|---|---|---|---|---|---|
| AA | D | I | P | N | P | I | G | S | E | N | S | E | K | T | T | M | P | L | W | AA |
| Total (unmod) | 30 | 36 | 38 | 42 | 48 | 51 | 51 | 53 | 55 | 57 | 58 | 59 | 60 | 57 | 55 | 55 | 55 | 43 | 41 | Total (unmod) |
| Total (mod) | 0 | 0 | 0 | 0 | 0 | 0 | 0 | 0 | 0 | 0 | 0 | 0 | 0 | 0 | 0 | 0 | 0 | 0 | 0 | Total (mod) |
| RM | | | | | | | | | | | | | | | | | | | | RM |
| UHT | | | | | | | | | | | | | | | | | | | | UHT |
| IF | | | | | | | | | | | | | | | | | | | | IF |

**Figure S7**: Protein sequence of $\alpha_{S1}$-casein without the signal peptide. The numbers below the sequence indicate how many peptides containing this specific residue were detected in all samples as unmodified (unmod) and/or modified (mod) in RM, UHT milk, and IF. Lysine residues of the protein are framed with a dotted line, and modification sites are fully framed.

| Position | 1 | 2 | 3 | 4 | 5 | 6 | 7 | 8 | 9 | 10 | 11 | 12 | 13 | 14 | 15 | 16 | 17 | 18 | 19 | 20 | 21 | 22 | 23 | 24 | 25 | 26 | 27 | 28 | 29 | 30 | Position |
|---|---|---|---|---|---|---|---|---|---|---|---|---|---|---|---|---|---|---|---|---|---|---|---|---|---|---|---|---|---|---|---|
| AA | I | L | N | K | P | E | D | E | T | H | L | E | A | Q | P | T | D | A | S | A | Q | F | I | R | N | L | Q | I | S | N | AA |
| Total (unmod) | 15 | 18 | 18 | 18 | 18 | 18 | 18 | 18 | 18 | 18 | 18 | 18 | 18 | 18 | 18 | 18 | 17 | 16 | 15 | 15 | 14 | 13 | 9 | 8 | 5 | 4 | 2 | 1 | 1 | 1 | Total (unmod) |
| Total (mod) | 6 | 7 | 7 | 7 | 7 | 7 | 7 | 7 | 7 | 7 | 6 | 5 | 4 | 3 | 3 | 3 | 2 | 2 | 2 | 2 | 2 | 2 | 2 | 2 | 1 | 1 | 0 | 0 | 0 | 0 | Total (mod) |
| RM | 1 | 1 | 1 | 1 | 1 | 1 | 1 | 1 | 1 | 1 | 1 | 1 | 1 | 1 | 1 | 1 | 1 | 1 | 1 | 1 | 1 | 1 | 1 | 1 | | | | | | | RM |
| UHT | 1 | 1 | 1 | 1 | 1 | 1 | 1 | 1 | 1 | 1 | 1 | 1 | 1 | 1 | 1 | 1 | 1 | 1 | 1 | 1 | 1 | 1 | 1 | 1 | | | | | | | UHT |
| IF | 6 | 7 | 7 | 7 | 7 | 7 | 7 | 7 | 7 | 7 | 6 | 5 | 4 | 3 | 3 | 3 | 2 | 2 | 2 | 2 | 2 | 2 | 2 | 2 | 1 | 1 | | | | | IF |

| Position | 31 | 32 | 33 | 34 | 35 | 36 | 37 | 38 | 39 | 40 | 41 | 42 | 43 | 44 | 45 | 46 | 47 | 48 | 49 | 50 | 51 | 52 | 53 | 54 | 55 | 56 | 57 | 58 | 59 | 60 | Position |
|---|---|---|---|---|---|---|---|---|---|---|---|---|---|---|---|---|---|---|---|---|---|---|---|---|---|---|---|---|---|---|---|
| AA | E | D | L | S | K | E | P | S | I | S | R | E | D | L | I | S | K | E | Q | I | V | I | R | S | S | R | Q | P | Q | S | AA |
| Total (unmod) | 1 | 1 | 1 | 1 | 1 | 1 | 1 | 1 | 1 | 1 | 1 | 1 | 1 | 2 | 2 | 2 | 2 | 2 | 2 | 2 | 2 | 2 | 2 | 8 | 11 | 11 | 16 | 16 | 16 | 19 | Total (unmod) |
| Total (mod) | 0 | 0 | 0 | 0 | 0 | 0 | 0 | 0 | 0 | 0 | 0 | 0 | 0 | 0 | 0 | 0 | 0 | 0 | 0 | 0 | 0 | 0 | 0 | 9 | 9 | 9 | 9 | 9 | 9 | 9 | Total (mod) |
| RM | | | | | | | | | | | | | | | | | | | | | | | | | | | | | | | RM |
| UHT | | | | | | | | | | | | | | | | | | | | | | | | 2 | 2 | 2 | 2 | 2 | 2 | 2 | UHT |
| IF | | | | | | | | | | | | | | | | | | | | | | | | 9 | 9 | 9 | 9 | 9 | 9 | 9 | IF |

| Position | 61 | 62 | 63 | 64 | 65 | 66 | 67 | 68 | 69 | 70 | 71 | 72 | 73 | 74 | 75 | 76 | 77 | 78 | 79 | 80 | 81 | 82 | 83 | 84 | 85 | 86 | 87 | 88 | 89 | 90 | Position |
|---|---|---|---|---|---|---|---|---|---|---|---|---|---|---|---|---|---|---|---|---|---|---|---|---|---|---|---|---|---|---|---|
| AA | Q | N | P | K | L | P | L | S | I | L | K | E | K | H | L | R | N | A | T | L | G | S | E | E | T | T | E | H | T | P | AA |
| Total (unmod) | 19 | 20 | 21 | 21 | 24 | 24 | 23 | 21 | 17 | 17 | 11 | 8 | 7 | 4 | 3 | 0 | 0 | 0 | 0 | 0 | 0 | 0 | 0 | 0 | 0 | 0 | 0 | 0 | 0 | 0 | Total (unmod) |
| Total (mod) | 9 | 9 | 9 | 9 | 9 | 9 | 9 | 8 | 8 | 8 | 7 | 6 | 6 | 2 | 2 | 0 | 0 | 0 | 0 | 0 | 0 | 0 | 0 | 0 | 0 | 0 | 0 | 0 | 0 | 0 | Total (mod) |
| RM | | | | | | | | | | | | | | | | | | | | | | | | | | | | | | | RM |
| UHT | 2 | 2 | 2 | 2 | 2 | 2 | 2 | 2 | 2 | 2 | 2 | 2 | 2 | | | | | | | | | | | | | | | | | | UHT |
| IF | 9 | 9 | 9 | 9 | 9 | 9 | 9 | 8 | 8 | 8 | 7 | 6 | 6 | 2 | 2 | | | | | | | | | | | | | | | | IF |

| Position | 91 | 92 | 93 | 94 | 95 | 96 | 97 | 98 | 99 | 100 | 101 | 102 | 103 | 104 | 105 | 106 | 107 | 108 | 109 | 110 | 111 | 112 | 113 | 114 | 115 | 116 | 117 | 118 | 119 | 120 | Position |
|---|---|---|---|---|---|---|---|---|---|---|---|---|---|---|---|---|---|---|---|---|---|---|---|---|---|---|---|---|---|---|---|
| AA | S | D | A | S | T | T | E | G | K | L | M | E | L | G | H | K | I | M | R | N | L | E | N | T | V | K | E | T | I | K | AA |
| Total (unmod) | 0 | 0 | 0 | 0 | 0 | 0 | 0 | 0 | 0 | 0 | 0 | 0 | 0 | 0 | 0 | 0 | 0 | 0 | 1 | 1 | 2 | 3 | 4 | 4 | 4 | 4 | 4 | 4 | 4 | 4 | Total (unmod) |
| Total (mod) | 0 | 0 | 0 | 0 | 0 | 0 | 0 | 0 | 0 | 0 | 0 | 0 | 0 | 0 | 0 | 0 | 0 | 0 | 2 | 2 | 4 | 6 | 6 | 6 | 6 | 6 | 6 | 6 | 6 | 6 | Total (mod) |
| RM | | | | | | | | | | | | | | | | | | | | | | | | | | | | | | | RM |
| UHT | | | | | | | | | | | | | | | | | | | | | | | | | | | | | | | UHT |
| IF | | | | | | | | | | | | | | | | | | | 2 | 2 | 4 | 6 | 6 | 6 | 6 | 6 | 6 | 6 | 6 | 6 | IF |

| Position | 121 | 122 | 123 | 124 | 125 | 126 | 127 | 128 | 129 | 130 | 131 | 132 | 133 | 134 | 135 | Position |
|---|---|---|---|---|---|---|---|---|---|---|---|---|---|---|---|---|
| AA | Y | L | K | S | L | F | S | H | A | F | E | V | V | K | T | AA |
| Total (unmod) | 4 | 1 | 2 | 3 | 3 | 4 | 5 | 6 | 6 | 6 | 6 | 6 | 6 | 6 | 6 | Total (unmod) |
| Total (mod) | 6 | 0 | 1 | 1 | 1 | 3 | 4 | 4 | 4 | 4 | 4 | 4 | 4 | 4 | 4 | Total (mod) |
| RM | | | | | | | | | | | | | | | | RM |
| UHT | | | | | | | | | | | | | | | | UHT |
| IF | 6 | | 1 | 1 | 1 | 3 | 4 | 4 | 4 | 4 | 4 | 4 | 4 | 4 | 4 | IF |

**Figure S8**: Protein sequence of GlyCAM-1 without the signal peptide. The numbers below the sequence indicate how many peptides containing this specific residue were detected in all samples as unmodified (unmod) and/or modified (mod) in RM, UHT milk, and IF. Lysine residues of the protein are framed with a dotted line, and modification sites are fully framed.
